# Organotin-Catalyzed Synthesis, Characterization, Anticancer and Antioxidant Activities, and Molecular Modeling Studies of Nimesulide Ureas as Potential MetAP (Type II) Inhibitors

**DOI:** 10.1021/acsomega.6c00645

**Published:** 2026-08-27

**Authors:** Yağmur Biliz, Özgür Yılmaz, Elif Kuloğlu, İpek Bedir, Egemen Çakırlı, Şevval Arslan, Dilek Telci, Özge Çevik, Kemal Yelekçi, Gani Koza, Ş. Güniz Küçükgüzel

**Affiliations:** † Department of Chemical Engineering, Faculty of Engineering and Natural Sciences, Istanbul Health and Technology University, Istanbul 34275, Turkiye; ‡ TUBITAK Marmara Research Center, Gebze, Kocaeli 41470, Turkiye; § Department of Pharmaceutical Chemistry, Faculty of Pharmacy, Istanbul University, Istanbul 34116, Turkiye; ∥ Department of Chemistry, Faculty of Art and Science, 37523 Bursa Uludağ University, Bursa 16059, Turkiye; ⊥ Department of Genetics and Bioengineering, 52998 Yeditepe University, Istanbul 34755, Turkiye; # Department of Biochemistry, School of Medicine, 52943 Aydın Adnan Menderes University, Aydın 09010, Turkiye; ¶ Department of Molecular Biology and Genetics, Faculty of Engineering and Natural Sciences, 52974 Kadir Has University, Istanbul 34083, Turkiye; ∇ Department of Pharmaceutical Chemistry, Faculty of Pharmacy, 519324 Fenerbahçe University, Atasehir, Istanbul 34758, Turkiye

## Abstract

In this study, a new series of nimesulide-derived ureas (**3a–j**) was designed and synthesized to investigate structural modifications of nimesulide and to evaluate their biological activity. The compounds were obtained in high yields (68–88%) under organotin-catalyzed conditions, and their structures were unequivocally established using comprehensive spectroscopic techniques, including ^1^H NMR, ^13^C NMR, FTIR, and HRMS. Following structural confirmation, the antioxidant properties of the synthesized derivatives were evaluated using the 2,2-diphenyl-1-picrylhydrazyl (DPPH) radical scavenging assay, where compounds **3e**, **3f**, and **3i** displayed notable radical-quenching activity. The nimesulide derivative **3i** exhibited notable cytotoxicity across multiple breast cancer models, including the triple-negative MDA-MB-231 and 4T1 cell lines, as well as the luminal MCF-7 subtype. Consistent with its enhanced potency, **3i** triggered a robust shift from cell viability to programmed cell death, characterized predominantly by a dramatic accumulation of late-apoptotic populations following 72 h of treatment. This apoptotic signature was especially pronounced in Triple-Negative Breast Cancer (TNBC) cells, in which viable cell fractions were nearly eliminated. These findings suggest that structural modification of the nimesulide derivative can substantially improve its in vitro anticancer efficacy. Taken together, the observed cytotoxic and apoptosis-inducing effects suggest that compound **3i** may represent a potential NSAID-based anticancer candidate. In line with its pronounced cytotoxic and pro-apoptotic profile, compound **3i** also demonstrated strong inhibition of the MetAP2 enzyme, further reinforcing its potential as a multifunctional nimesulide-based anticancer agent. To obtain insight into the binding pose and binding energy of all synthesized compounds (3a−j) were docked into the active site of the MetAP2 enzyme. The computational inhibition constant values were correlated with the experimental values. To test the dynamic behavior of MetAP2-inhibitor complexes, a molecular dynamics (MD) simulation was also carried out for a 200 ns duration. MD revealed that the drugs bind to the active site of the MetAP2 enzyme, as indicated by stable RMSD and RMSF plots. In conclusion, *in silico* results and in vitro studies suggest that the nimesulide derivatives may be novel, potential NSAID-based anticancer drug candidates for treating breast, prostate, gastric, and glioblastoma.

## Graphical abstract

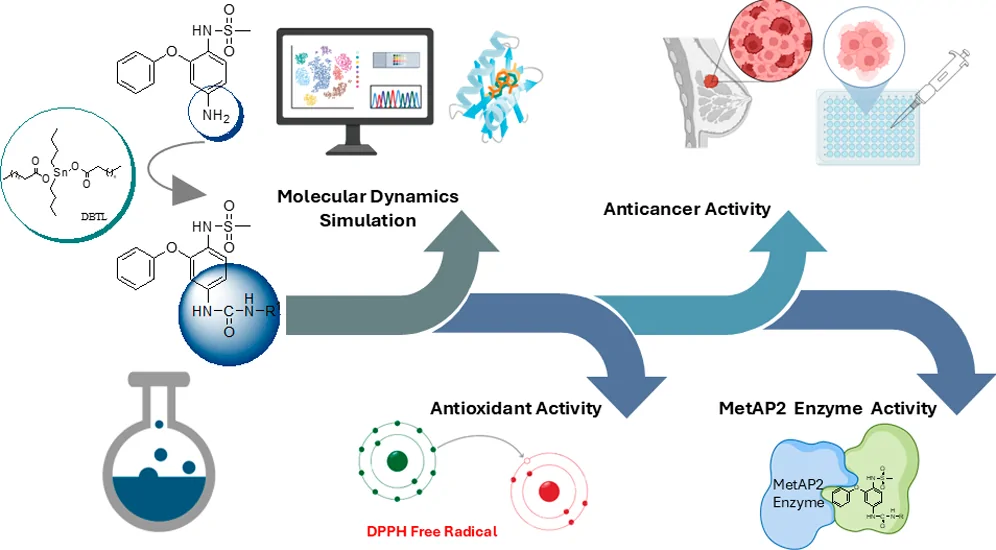

## 1 Introduction

Nonsteroidal anti-inflammatory drugs (NSAID) represent a major class of therapeutic agents extensively employed in the management of pain, pyrexia, and various inflammatory disorders.^1^ Accumulating clinical evidence suggests that the use of nonsteroidal anti-inflammatory drugs (NSAIDs) is correlated with a reduced incidence of breast, lung, prostate, and colorectal cancers.^2^ Within the class of nonsteroidal anti-inflammatory drugs (NSAIDs), nimesulide has emerged as a compound of significant pharmacological interest owing to its pronounced antiproliferative potential against diverse cancer cell types.^3^ Chronic exposure to nonsteroidal anti-inflammatory drugs (NSAIDs) is well-documented to induce gastrointestinal side effects. However, accumulating pharmacological evidence suggests that nimesulide displays a lower rate of gastrointestinal complications relative to conventional NSAIDs.^4^ Reactive oxygen species (ROS) play a complex role in cancer biology, contributing to both tumor development and cell death depending on their intracellular levels. On the other hand, persistent inflammation induced by elevated oxidative stress has been associated with the development of various diseases, including cancer, diabetes, neurological disorders, and cardiovascular diseases. There is a well-established relationship between cancer and the excessive production of free radicals, highlighting the potential relevance of antioxidants in cancer research. Nevertheless, although antioxidant compounds can modulate cellular redox balance, their direct contribution to anticancer mechanisms remains complex and has not yet been fully elucidated. In this context, nimesulide has been reported to exhibit antioxidant activity, which has been attributed in some studies to its sulfonanilide moiety.^2,5^ Despite being considered one of the most gastrointestinally tolerable NSAIDs, the major adverse reaction associated with nimesulide is hepatotoxicity.^6^ In the liver, nimesulide is metabolized via the cytochrome P450 enzymatic system; however, the mechanism underlying its hepatotoxicity has not been fully elucidated, and it has been suggested that multiple metabolic pathways, including reactive metabolites formed during nitro-reduction processes, may contribute to this effect.^7,8^ These adverse metabolic processes indicate that the nimesulide scaffold is amenable to structural modification. Indeed, the literature reports that nimesulide-derived urea and thiourea analogues exhibit significant antiproliferative activities across multiple cancer cell models. For instance, the synthesized nimesulide–acylthiourea hybrids demonstrated that structural modification can markedly enhance the pharmacological potential of nimesulide, as evidenced by their pronounced enzyme inhibition profiles and broad spectrum of biological activities.^9–11^ Similarly, aryl-urea and nitroaryl-urea derivatives have exhibited pronounced antiproliferative properties across various cancer cell lines; notably, compounds bearing a nitroaryl-urea core were reported to display higher cytotoxic activity than cisplatin in RK33 laryngeal carcinoma and TE671 rhabdomyosarcoma cells.^12^ A stronger connection between the chemical structure of the nimesilude scaffold and observed biological activity was reported. All these well-established studies encourage us to synthesize some novel nimesulide urea derivatives and test them against the MetAP2 enzyme. Nimesulide analogues: From anti-inflammatory to antitumor agents.^13–15^ Moreover, the literature demonstrates that aryl-urea derivatives incorporating urea or urea-like linker groups enhance biological activity and provide critical hydrogen-bonding interactions that facilitate binding to cellular targets. Consistently, the study by Klinkhammer and colleagues reported that ortho-nitro aryl derivatives containing a urea linker exhibit markedly higher biological potency compared to their structural analogues.^16^ Taken together, these findings indicate that the incorporation of urea functionalities into the nimesulide scaffold, particularly in conjunction with the sulfonanilide moiety, may contribute to antiproliferative activity and the enzyme inhibition profile.

In this context, molecular regulators of cancer cell proliferation have gained increasing attention, with methionine aminopeptidase 2 (MetAP2) recognized as a key enzyme in this process. Despite this relevance, reports on the potential inhibitory effects of nimesulide or its urea derivatives on MetAP2 remain scarce. Accordingly, we performed molecular modeling and binding studies to explore the interactions between the synthesized nimesulide-urea derivatives and MetAP2. Compounds showing the most favorable binding poses and the highest cytotoxic activities were subsequently selected for experimental evaluation of their MetAP2 inhibitory properties.

Our recently published study evaluated a distinct series of nimesulide derivatives across multiple cancer cell lines and revealed particularly promising activity against breast cancer cells.^15^ Based on these findings, the present study was specifically focused on breast cancer, with MetAP2 considered as a potential molecular target. While both studies explore the anticancer potential of nimesulide-derived compounds, they involve different compound series and address different research objectives, with the present study specifically investigating the potential role of MetAP2 in the observed anticancer activity.

In this work, a series of novel nimesulide-derived urea compounds (**3a–j**) were developed and evaluated for their biological activity (Figure 1). Their biological activities were then assessed through cytotoxicity assays on selected cancer cell lines, complemented by DPPH-based antioxidant evaluations and apoptosis studies. Subsequently, molecular modeling was performed to explore the interactions of the compounds with MetAP2, and the derivatives exhibiting the most favorable binding profiles were further examined for their MetAP2 inhibitory activity.

**Figure 1:**
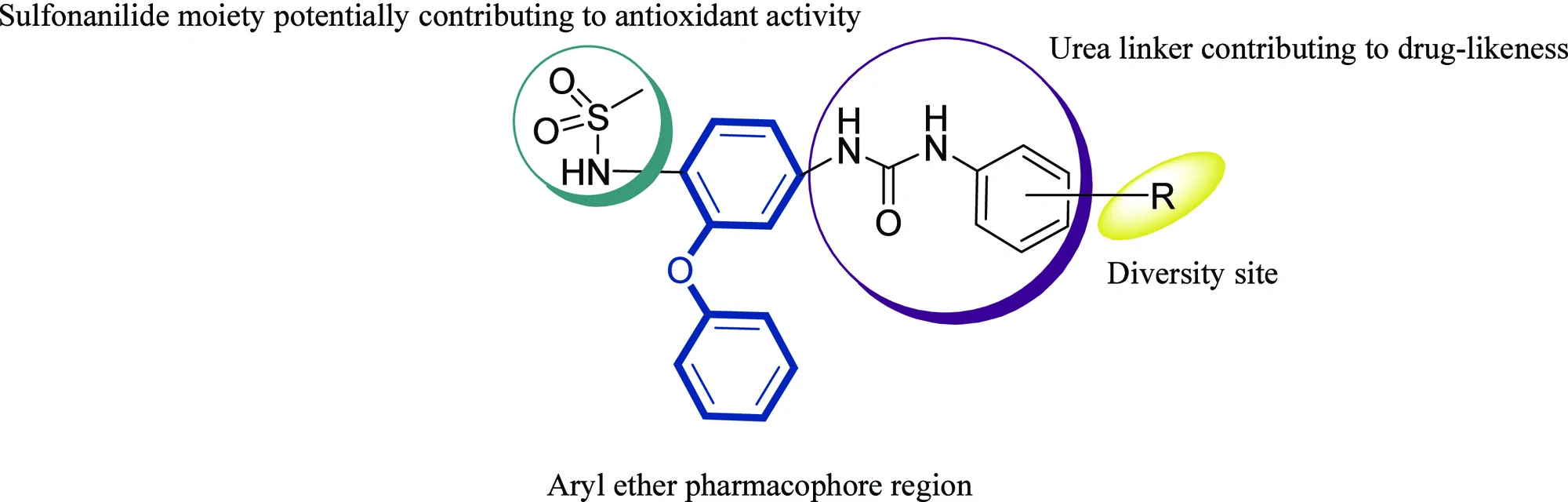
Design strategy for the novel synthesized target compounds (**3a**–**j**).

## 2 Results and Discussion

### 2.1 Chemistry

In the presence of various coupling agents, a series of novel nimesulide-derived ureas (**3a–j**)^15,17^ were synthesized via the reaction of reduced nimesulide with aromatic isocyanates, affording the target compounds in yields ranging from 68% to 88%, as outlined in Scheme 1.

**Scheme 1:**
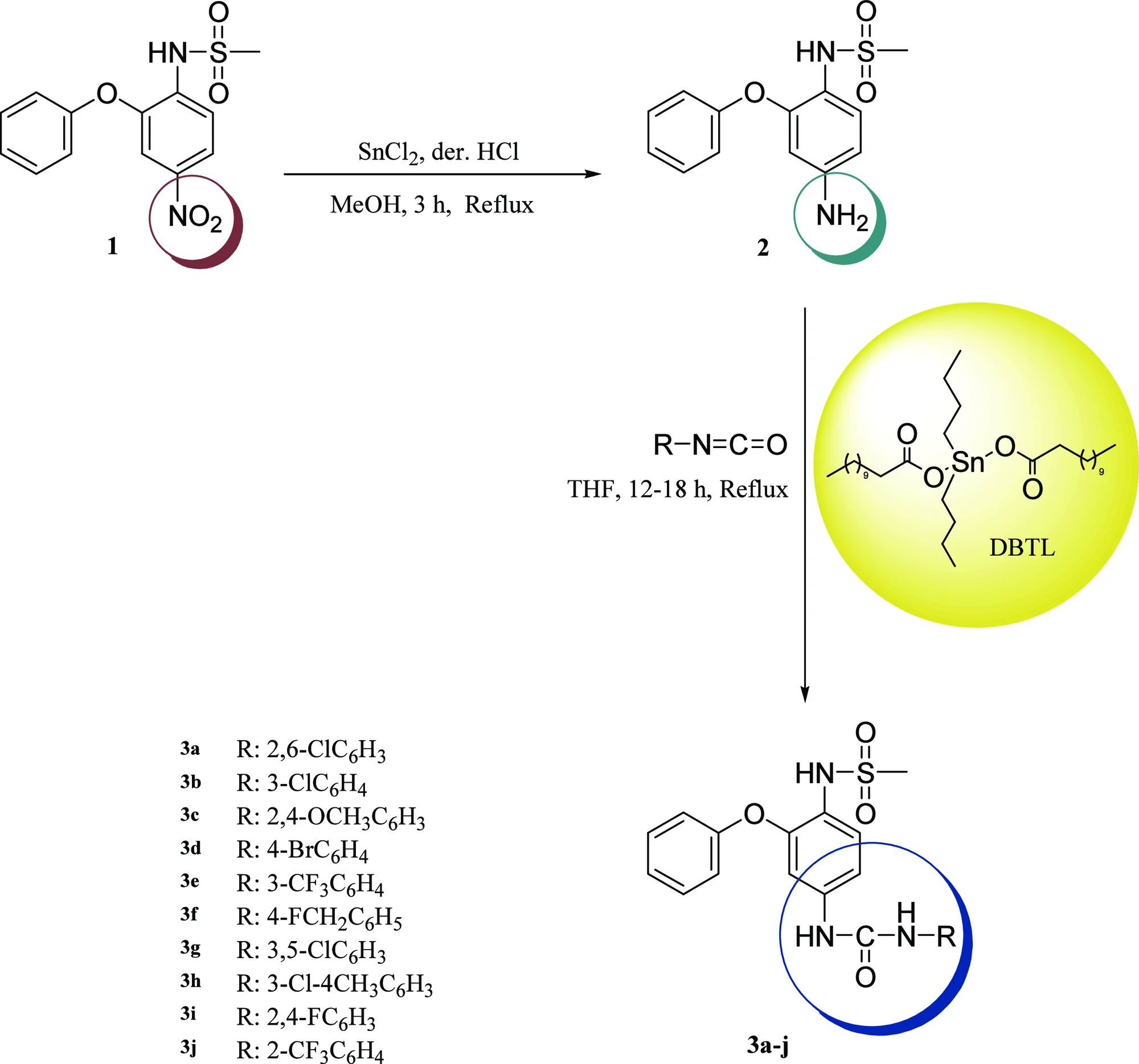
General Synthetic Route to the Novel Nimesulide-Derived Urea Derivatives (**3a**–**j**)

Nimesulide was hydrogenated in the presence of tin­(II) chloride (SnCl_2_), yielding the reduced derivative (**2**).^15–17^ The obtained reduced form of nimesulide contains an aromatic primary amine group whose nucleophilicity is considerably decreased due to delocalization of the nitrogen lone pair into the aromatic π-system. This electronic delocalization markedly diminishes its reactivity, resulting in substantially lower product yields when the reaction is carried out without a catalyst Optimization of the reaction conditions was carried out using compound **3j** as a model substrate under various catalytic systems, including DBTL (dibutyltin dilaurate), HBTU (*O*-(benzotriazol-1-yl)-*N*,*N*,*N*′,*N*′-tetramethyluronium hexafluorophosphate)/DIPEA (*N*,*N*-diisopropylethylamine), HCTU (*O*-(6-chloro-1*H*-benzotriazol-1-yl)-*N*,*N*,*N*′,*N*′-tetramethyluronium hexafluorophosphate)/DIPEA, DIC (*N*,*N*′-diisopropylcarbodiimide)/NHS (*N*-hydroxysuccinimide), DIC/OXYMA (ethyl 2-cyano-2-(hydroxyimino)­acetate), and DCC (*N*,*N*′-dicyclohexylcarbodiimide)/OXYMA. Among these systems, DBTL demonstrated the highest catalytic efficiency, providing the desired product in 78% yield under the optimized conditions (molar ratio of compound **2**: 1.0 equiv in all experiments, see Table 1 footnote).

**Table 1:**
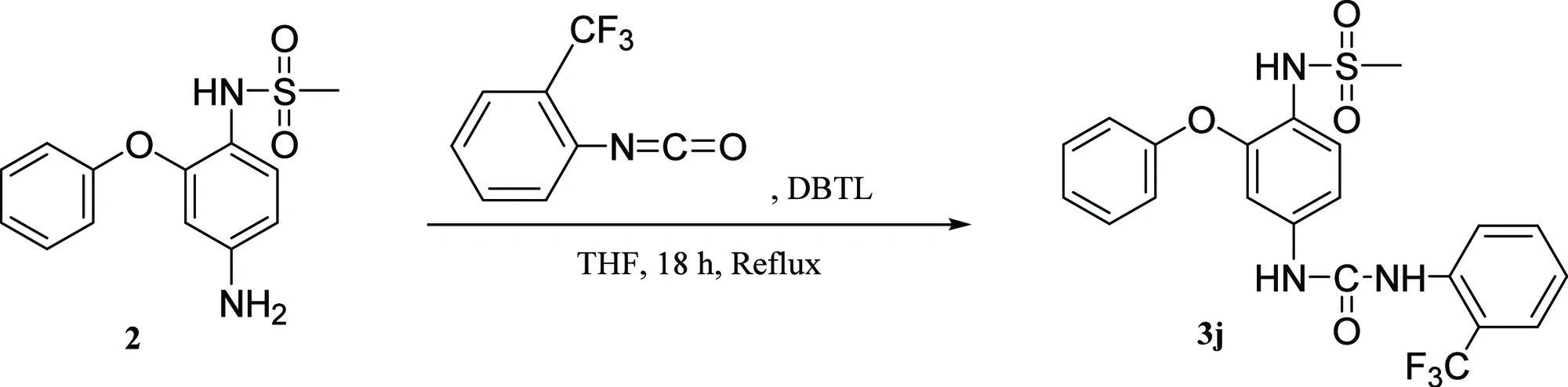
Optimization of Reaction Conditions for the Synthesis of Nimesulide-Derived Urea Derivatives^a^

entry	isocyanate (equiv)	catalyst (equiv)	catalyst	solvent	*t* (°C)	time (h)	yield (%)
1	1.1	1.0	DBTL	THF	66	18	51
2	1.1	1.5	DBTL	THF	66	18	70
3	1.5	1.5	DBTL	THF	66	18	78
4	1.1	2.0/4.0	HBTU/DIPEA	THF	66	18	25
5	1.1	4.0/8.0	HBTU/DIPEA	THF	66	18	30
6	1.1	4.0/8.0	HCTU/DIPEA	THF	66	18	29
7	1.1	2.0/4.0	DIC/NHS	THF	66	12	30
8	1.1	4.0/8.0	DIC/NHS	THF	66	12	59
9	1.1	4.0/8.0	DIC/OXYMA	THF	66	12	63
10	1.1	4.0/8.0	DCC/NHS	THF	66	12	66
11	1.1	4.0/8.0	DCC/OXYMA	THF	66	12	69

a Molar ratio of compound **2**: 1.0.

Structural elucidation of the synthesized nimesulide-derived ureas (**3a**–**j**) was achieved through a combination of IR, ^1^H NMR, ^13^C NMR, and HR-MS spectroscopic techniques (Figures S5–S44, Supporting Information).

The structural characterization of reduced nimesulide (**2**) was confirmed through comprehensive spectroscopic analyses, including FT-IR, ^1^H NMR, ^13^C NMR, and HR-MS. In the FT-IR spectrum, two absorption bands were observed at approximately 3397 and 3330 cm^–1^, which are attributed to the **N–H** stretching vibrations of the amine functionality. The ^1^H NMR spectrum displayed a singlet at δ 5.26 ppm corresponding to the aromatic primary amine (Ar–N **H**_2_) protons. The aliphatic and aromatic carbon resonances appeared at their expected chemical shift values, confirming the proposed structure. Moreover, the MS spectrum of compound **2** showed molecular ion peaks consistent with the calculated molecular mass, further verifying the identity of the reduced product.

Upon examination of the FT-IR spectra, strong absorption peaks corresponding to the **C=O** stretching vibrations of the urea linkage were detected in the range of 1635–1661 cm^–1^. In all target compounds, weak absorption bands corresponding to the **N–H** stretching vibrations were detected between 3410 and 3251 cm^–1^. The characteristic peaks corresponding to the aromatic ring vibrations were observed in the ranges of 3096–3051 and 1661–1500 cm^–1^.

Examination of the ^1^H NMR spectra of nimesulide-derived ureas (**3a**–**j**) revealed singlet resonances corresponding to the S–C **H**_3_ protons in the δ 2.91–3.01 ppm range. Singlet resonances attributable to Ar–N **H**–S and Ar–N **H**–CO protons were detected in the ^1^H NMR spectra, positioned at δ 8.78–9.18 ppm and δ 9.17–9.43 ppm, respectively. The CO–N **H**–R protons in the synthesized nimesulide-based ureas were detected as singlet resonances, exhibiting substituent-dependent variations in their chemical shift values. In the benzyl-substituted compound (**3f**), this proton (CO–N **H**–R) appeared at δ 6.62 ppm, whereas in the aromatic-substituted derivatives (**3a**, **3b**, **3c**, **3d**, **3e**, **3g**, **3h**, **3i**, and **3j**), the corresponding resonances were observed downfield at δ 7.96–8.96 ppm. Examination of the ^1^H NMR spectra revealed that the aromatic protons resonated as characteristic doublets, triplets, and multiplets spanning the δ 6.43–8.00 ppm range, consistent with the expected substitution pattern of the aromatic ring.

Resonances assigned to the S–**C** H_3_ carbon atoms were detected at δ 37.61–43.41 ppm. Signals corresponding to the NH–**C** =O carbon atoms appeared in the region of δ 155.83–164.44 ppm, whereas those of the aromatic carbons were observed within δ 98.10–160.36 ppm.

In the HR-MS spectra, the molecular ion peaks of compounds **3a–j** were found to be in complete agreement with their theoretical molecular weights, confirming their molecular compositions.

The catalytic role of DBTL in the reaction between alcohols and isocyanates has been extensively investigated through studies on the reaction mechanism and kinetics.^11,18–24^ In this context, the proposed mechanism for the reaction of reduced nimesulide with substituted isocyanates in the presence of DBTL begins with the coordination of DBTL to the carbonyl oxygen of the isocyanate, acting as a Lewis acid. This coordination enhances the electrophilicity of the carbonyl carbon of the isocyanate. Subsequently, the primary amine group of reduced nimesulide performs a nucleophilic attack on the activated carbonyl carbon of the isocyanate. The resulting intermediate leads to the formation of the urea bond following a proton transfer step. In the final stage, DBTL dissociates from the reaction medium and re-enters the catalytic cycle (Figure 2).

**Figure 2:**
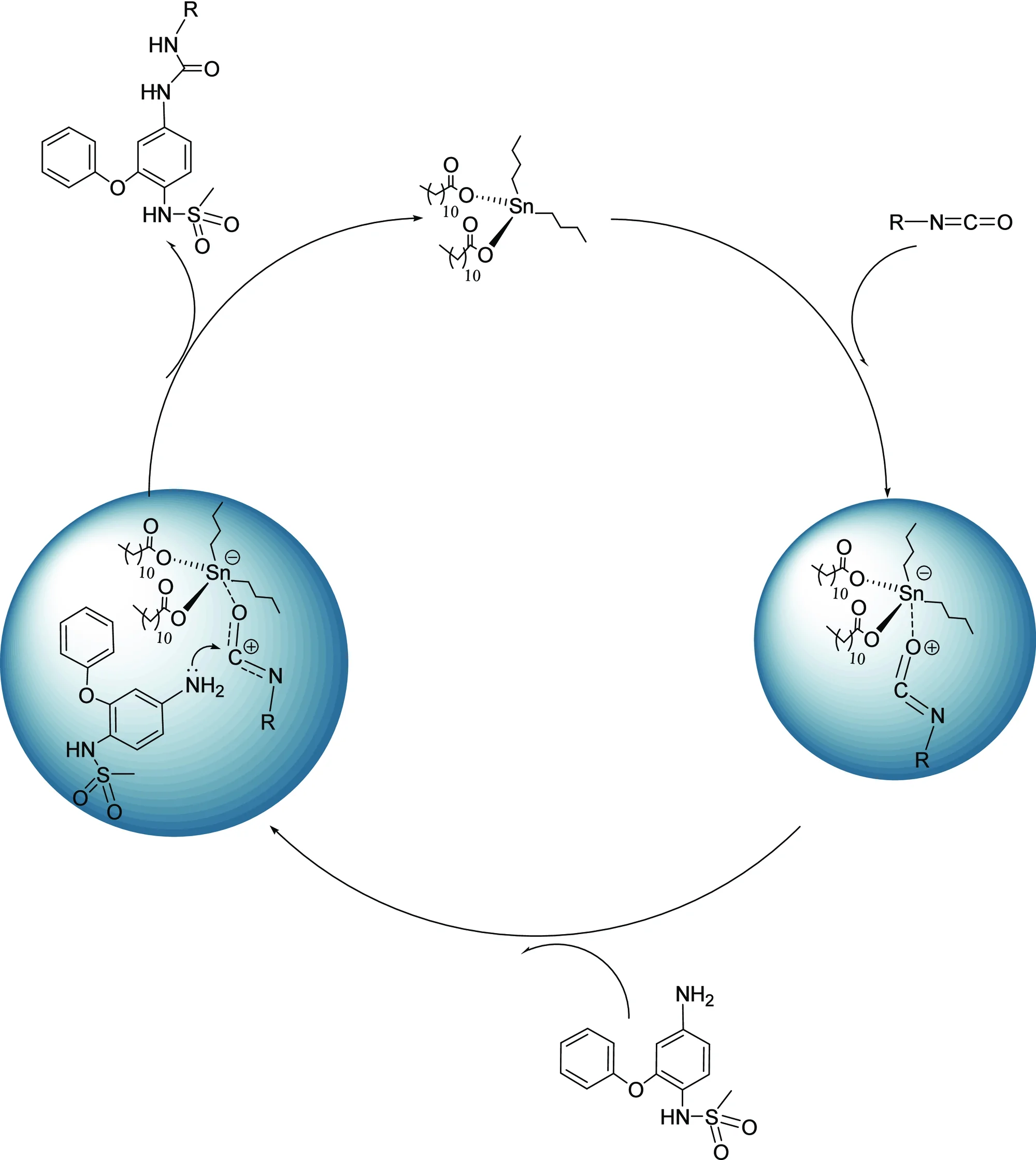
Proposed mechanism for nimesulide-based urea synthesis under organotin catalysis.

### 2.2 Antioxidant Activity

Free radicals are highly reactive and unstable molecular species generated through normal metabolic pathways in living systems, primarily due to the presence of unpaired electrons. Their excessive accumulation can initiate oxidative stress, leading to cellular and molecular damage. Antioxidants, on the other hand, are bioactive molecules capable of neutralizing or preventing the harmful effects induced by these reactive oxygen species. Several reports have demonstrated the antioxidant potential of nimesulide and its derivatives.^25^ In the present work, the antioxidant properties of the newly synthesized nimesulide-based ureas (**3a–j**) were investigated and compared with well-known antioxidant standards ascorbic acid, quercetin, butylated hydroxytoluene (BHT), and butylated hydroxyanisole (BHA) using the DPPH radical scavenging method. DPPH (1,1-diphenyl-2-picrylhydrazyl) is a stable nitrogen-centered free radical characterized by a deep violet color, showing a strong absorption maximum at 517 nm in methanol (see Table 2 footnote).^2,15^ Upon interaction with electron- or hydrogen-donating antioxidant compounds, the radical is reduced, resulting in a gradual color change from purple to yellow.

**Table 2:** DPPH Scavenging Activities of Novel Nimesulide Ureas (**3a**–**j**)­^a^

		DPPH (μM/mL)		
entry	compounds and standards	250	500	1000
1	**3a**	46.83 ± 0.91	48.75 ± 7.72	51.08 ± 2.20
2	**3b**	31.20 ± 3.23	39.88 ± 3.08	42.70 ± 9.94
3	**3c**	51.36 ± 4.56	53.12 ± 4.79	71.07 ± 2.80
4	**3d**	41.55 ± 8.33	53.38 ± 3.70	68.39 ± 5.58
5	**3e**	**80.48** **±** **2.07**	**83.18** **±** **3.96**	**86.85** **±** **1.05**
6	**3f**	**74.35** **±** **0.68**	**86.45** **±** **0.68**	**90.28** **±** **7.55**
7	**3g**	38.89 ± 2.41	52.97 ± 3.07	71.01 ± 4.30
8	**3h**	27.28 ± 6.24	43.85 ± 2.19	48.15 ± 2.17
9	**3i**	**38.28** **±** **1.22**	**56.35** **±** **4.75**	**83.38** **±** **0.73**
10	**3j**	25.11 ± 1.23	30.19 ± 2.44	42.80 ± 4.14
11	**BHT**	95.10 ± 1.52	96.22 ± 2.63	97.13 ± 3.00
12	**BHA**	94.39 ± 0.52	96.87 ± 0.69	97.51 ± 0.50
13	**Quercetin**	91.41 ± 0.36	91.66 ± 0.27	91.80 ± 0.67
14	**Ascorbic acid**	97.51 ± 0.43	98.01 ± 1.74	98.76 ± 0.45

a The reference antioxidant standard values (BHT, BHA, ascorbic acid, and quercetin) are identical to those reported in refs 2 and 15 , as they were determined under identical experimental conditions using the same analytical procedure. All data related to the synthesized compounds are original to the present study.

To evaluate the scavenging activity, methanolic solutions of the synthesized compounds and reference antioxidants were prepared at three concentrations (250, 500, and 1000 μM/mL). All measurements were performed in triplicate to ensure reproducibility (Table 2). Among the tested derivatives, compounds **3e**, **3f**, and **3i** exhibited radical scavenging activities comparable to those of the reference antioxidants at a concentration of 1000 μM/mL.

According to the mechanism proposed in this study, the NH groups in the urea derivative (**3a**–**j**) interact with the DPPH radical by donating a hydrogen atom. As a result of this process, the DPPH radical is reduced to its DPPH-H form, while a nitrogen-centered radical is formed in the corresponding compound. The resulting radical species is stabilized through resonance effects within the molecule. In this context, resonance interaction between the radical center and the carbonyl group contributes to the structural stability. Additionally, in systems containing an aromatic ring, delocalization of the radical center over the π-electron system further enhances stabilization. Therefore, the observed different structures represent the resonance forms of the radical species, and the antioxidant activity is fundamentally based on this delocalization and stabilization mechanism (Figure 3).^26,27^

**Figure 3:**
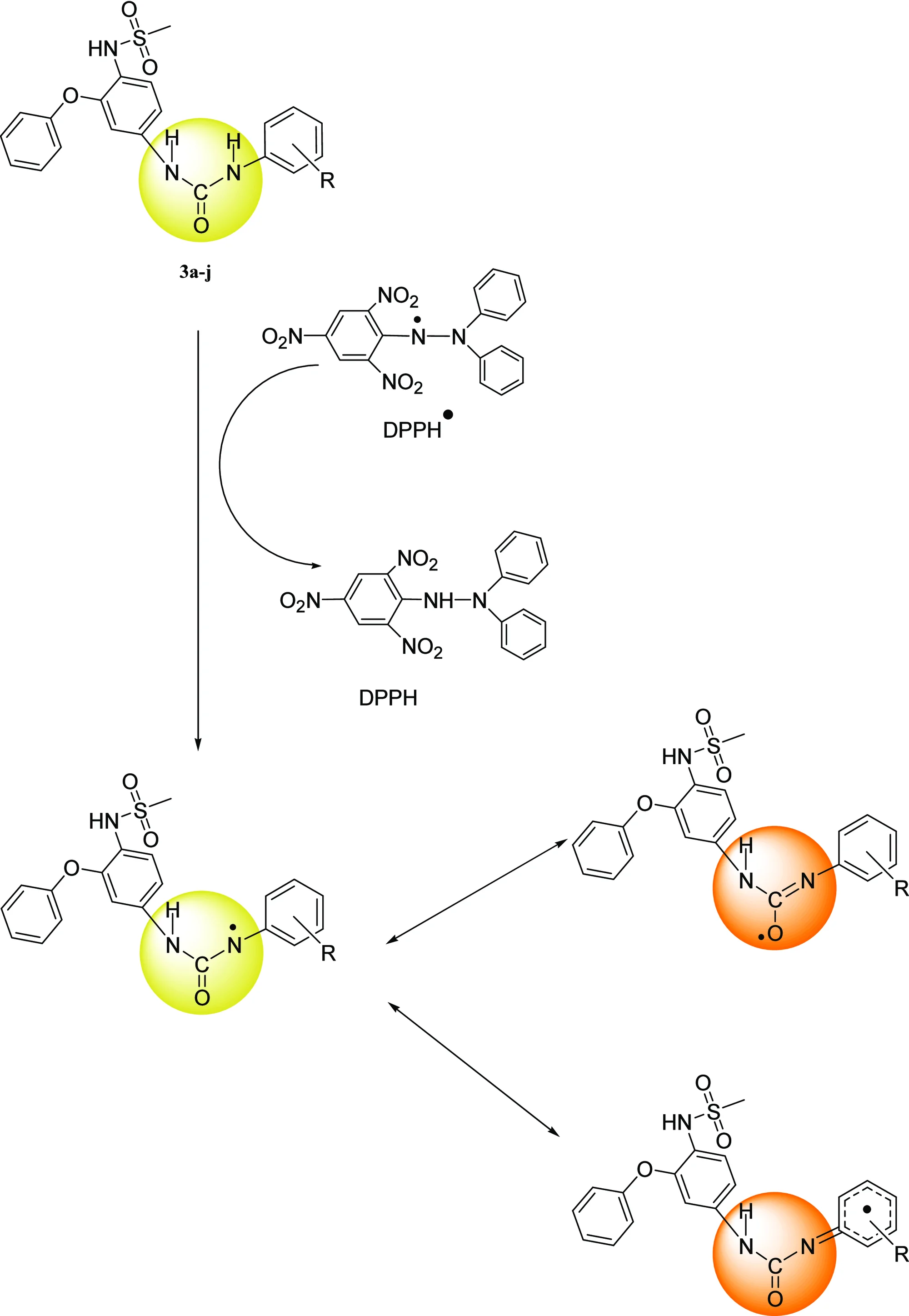
Proposed DPPH radical scavenging mechanism of nimesulide urea derivatives.

### 2.3 Cytotoxicity Assay

The cytotoxic effect of nimesulide derivatives **3a**–**j** on MDA-MB-231 and HEK293 cells was determined using a cell proliferation WST-1 assay. **3e** completely abolished HEK293 cell viability, whereas **3b**, **3h**, **3g**, **3d**, and **3j** markedly reduced cell viability to 3.23 ± 2.94%, 2.44 ± 0.85%, 2.26 ± 1.72%, 7.67 ± 5.61% and 23.96 ± 6.54%, respectively. In contrast, **3a**, **3c**, **3i**, and **3f** maintained viability rates of 89.24 ± 8.60%, 110.06 ± 3.36%, 99.79 ± 5.07% and 90.47 ± 4.22%, respectively, and showed no significant cytotoxicity (Figure 4). According to these findings, the nimesulide derivatives **3a**, **3c**, **3i**, and **3f**, which exhibited low toxicity toward HEK293 cells, were selected for further evaluation on the triple-negative breast cancer cell line MDA-MB-231. Although HEK293 cells are transformed embryonic kidney cells and may not fully represent normal human tissue, they were used in this study as a noncancerous reference cell line to allow comparison of cytotoxic effects between cancerous and noncancerous cells.

**Figure 4:**
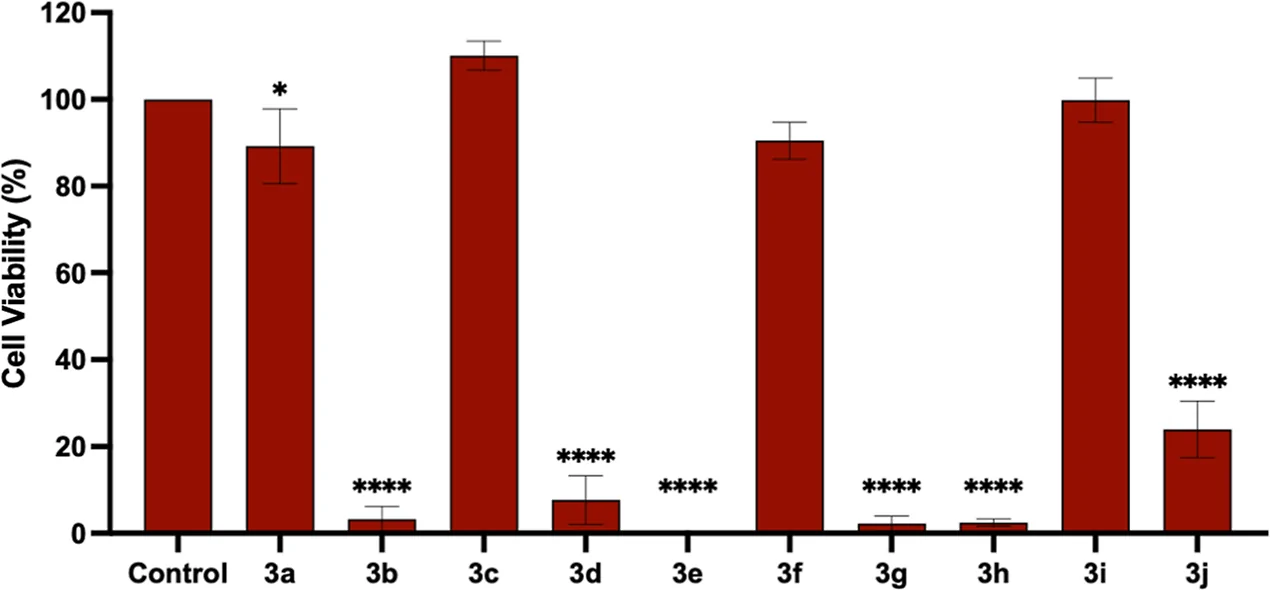
Cell viability of HEK293 cells following treatment with nimesulide derivatives **3a–j**, as determined by the WST-1 assay. HEK293 cells were treated with the indicated nimesulide derivatives for 72 h, and cell viability was measured using the WST-1 assay. Values represent mean ± SD from three independent experiments performed in triplicate. (*P ≤ 0.05, ****P ≤ 0.0001).

In MDA-MB-231 cells, treatment with **3a** and **3f** resulted in viability rates of 42.15 ± 10.80% and 47.24 ± 1.32%, respectively. Compound **3c** exhibited a viability rate of 87.35 ± 7.21%, indicating limited toxicity against this triple-negative breast cancer cell line. Conversely, **3i** demonstrated the most pronounced cytotoxic effect, reducing cell viability to 1.79 ± 0.74% (Figure 5). Based on the obtained dose–responses for **3a**, **3c**, **3f**, and **3i**, IC_50_ values were calculated to determine their half-maximal inhibitory concentrations (Table 3). Taken together, compound **3i** was selected for subsequent analyses due to its strong antiproliferative activity across the tested breast cancer cell lines, particularly in MDA-MB-231 and MCF-7 cells.

**Figure 5:**
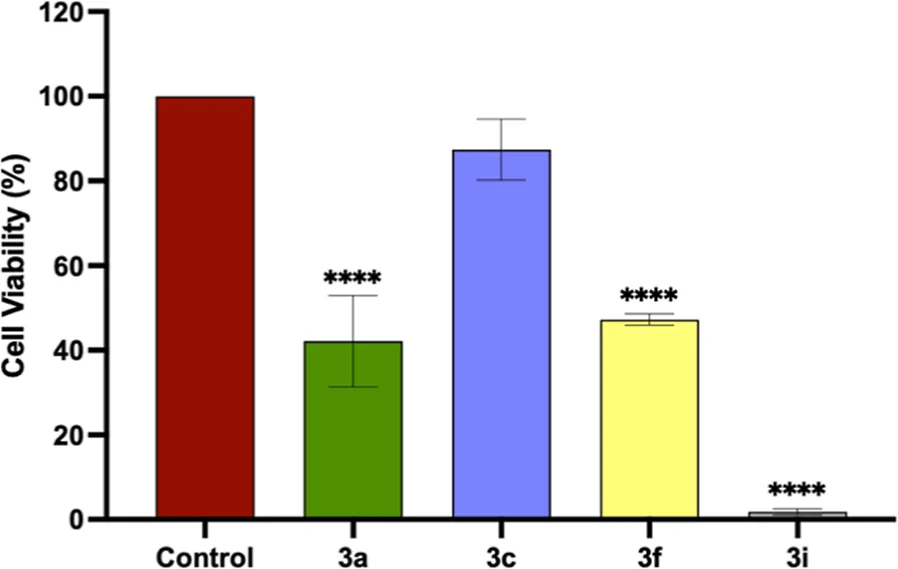
Cell viability of MDA-MB-231 cells following treatment with selected nimesulide derivatives **3a**, **3c**, **3f** and **3i**, as determined by the WST-1 assay. MDA-MB-231 cells were treated with **3a**, **3c**, **3i**, and **3f** for 72 h, and cell viability was assessed using the WST-1 assay. Values represent mean ± SD from three independent experiments performed in triplicate. (****P ≤ 0.0001).

**Table 3:** IC_50_ Values (μM) of Novel Nimesulide Derivatives

comp. no	MDA-MB231	4T1	MCF-7
**3a**	33.6 ± 3.0	-	-
**3c**	69.9 ± 10.1	-	-
**3f**	35.8 ± 3.5	-	-
**3i**	1.4 ± 0.1	16.3 ± 1.6	1.0 ± 0.1

To evaluate its efficacy in another triple-negative breast cancer model, we examined the cytotoxic activity of compound **3i** in the mouse-derived 4T1 cell line (Figure 6). Compound **3i** exhibited marked cytotoxicity, reducing cell viability to 2.41 ± 0.89% after 72 h. To determine whether its anticancer effects extend beyond triple-negative breast cancer, we next tested **3i** in the estrogen receptor–positive breast cancer cell line MCF-7. Cell viability was reduced to 29.08 ± 3.11% after 72 h of treatment, as assessed by the WST-1 proliferation assay, indicating that also exerted a substantial antiproliferative effect in this cell line. The findings of this study indicate that **3i** exerts variable degrees of antiproliferative activity across breast cancer subtypes, with a notably cytotoxic effect observed against triple-negative breast cancer cell lines.

**Figure 6:**
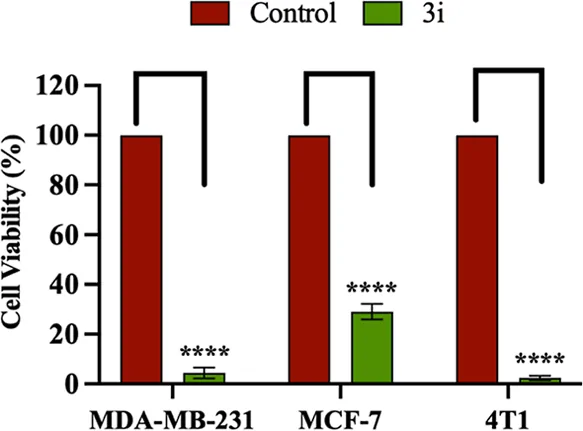
Cell viability of MCF-7 and 4T1 breast cancer cell lines following treatment with nimesulide derivative **3i**, as determined by the WST-1 assay. MCF-7 and 4T1 cells were treated with **3i** for 72 h, and cell viability was assessed using the WST-1 assay. Values represent mean ± SD from three independent experiments performed in triplicate. (****P ≤ 0.0001).

Building on the synthesis of these derivatives, the study aimed to characterize their anticancer properties through comprehensive evaluation of cytotoxicity distinct breast cancer subtypes. In line with the objective, treatment of TNBC cell lines (MDA-MB-231 and 4T1) and luminal-A MCF-7 cells with the nimesulide derivative **3i** resulted in a pronounced, dose-dependent decrease in cell viability, demonstrating substantially greatest potency compared to the other derivatives. This enhanced antiproliferative profile of **3i** can be rationalized by examining its distinct substitution pattern. In this analogue, the para-nitro group of the nimesulide scaffold has been replaced by a urea side chain bearing a 2,4-difluorophenyl substituent. Although the theoretical design of this substitution aimed to remove the nitroaromatic moiety conventionally associated with metabolic activation the definitive impact of this structural modification on mitigating idiosyncratic toxicity necessitates further empirical validation through comprehensive in vivo safety evaluations and detailed metabolic profiling in subsequent investigations. Within the scope of the current in vitro findings, this strategic substitution is primarily considered to optimize the electronic and lipophilic characteristics of the sulfonanilide core while introducing an additional, directionally constrained hydrogen-bonding motif. These changes are considered to potentially influence interactions with relevant molecular targets.^28–30^ As a result, the increased antiproliferative and pro-apoptotic activities of **3i**, when compared to both the parent scaffold and other derivatives, can be attributed to this specific 2,4-difluorophenyl–urea modification. Nimesulide, itself has been reported to be only mildly cytotoxic in various carcinoma models, with an IC_50_ of 75 μM after 96 h in BxPC-3 pancreatic adenocarcinoma cells,^31^ and IC_50_ values of approximately 100–150 μM in breast cancer cell lines^32^ whereas nimesulide derivative **3i** exhibited markedly enhanced potency with IC_50_ values of 1.0 ± 0.1 μM in MCF-7 cells, 1.4 ± 0.1 μM in MDA-MB-231 cells, and 16.3 ± 1.6 μM in 4T1 cells. In other studies, an IC_50_ value of 136 μM for nimesulide at 72 h has been reported in epidermoid carcinoma KB cells derived from oral squamous cell carcinoma (OSCC)^33^ and also higher IC_50_ values have been reported in BJ normal fibroblasts (590 μM), in SCC-15 (427 μM),^34^ and A431 (265.6 μM) squamous carcinoma cells at 72 h,^35–37^ these findings demonstrate that derivative **3i** achieves 50% growth inhibition at low micromolar concentrations and at earlier time points compared with nimesulide, underscoring its substantially greater promising anticancer potency.

The antitumor activity of compound **3i** was evaluated in vitro through apoptosis assays. MDA-MB-231, MCF-7, and 4T1 cells were treated with **3i** at its maximum tested concentration (40 μg/mL) or with 1:1000 diluted DMSO (vehicle control) for 72 h. Cell death was quantified using the Muse Annexin V & Dead Cell Assay on a Guava Muse Cell Analyzer (Merck Millipore, Germany) (Figure 7). In MDA-MB-231 cells, **3i** treatment significantly reduced the proportion of live cells from 94.18 ± 7.43% to 8.68 ± 3.27% (*p* < 0.0001), while necrotic cells increased from 2.37 ± 2.58% to 8.09 ± 0.51% (*p* > 0.05). In response to 72 h-**3i** treatment, early apoptosis rose modestly from 1.17 ± 0.34% to 2.77 ± 2.43%, whereas late apoptosis increased dramatically from 2.46 ± 1.06% to 80.70 ± 5.46% (*p* < 0.0001). In MCF-7 cells, 72 h of treatment with compound **3i** significantly reduced cell viability from 72.26 ± 0.78% to 24.86 ± 5.72%. Necrosis increased modestly from 7.64 ± 5.17% to 12.82 ± 10.64%, while early apoptosis rose from 1.00 ± 0.41% to 3.01 ± 2.01%. Notably, late apoptosis increased substantially in MCF-7 cells, from 19.34 ± 4.41% to 59.31 ± 13.98% following **3i** treatment. In 4T1 cells, live cell percentages significantly dropped from 81.86 ± 10.18% to 7.72 ± 10.18% as early apoptosis increased from 4.59 ± 0.60% to 12.85 ± 12.16%, and late apoptosis significantly inclined from 8.19 ± 3.46% to 76.73 ± 12.77% (*p* < 0.0001). While no significant change was reported for necrosis (5.36 ± 7.33% to 2.72 ± 0.38%) in response to **3i** treatment. Overall, **3i** treatment induced a pronounced shift toward late apoptosis across all tested cell lines, with the most striking effects observed in the triple-negative MDA-MB-231 and 4T1 cells. Although compound **3i** displayed low-micromolar activity in both MDA-MB-231 and MCF-7 cells, a higher IC_50_ value was observed in 4T1 cells. Notably, differences between cell lines became more evident when considering the magnitude of response at fixed concentrations and the apoptosis readouts, rather than IC_50_ values alone, which may reflect intrinsic biological differences between breast cancer subtypes and their apoptotic susceptibility.

**Figure 7:**
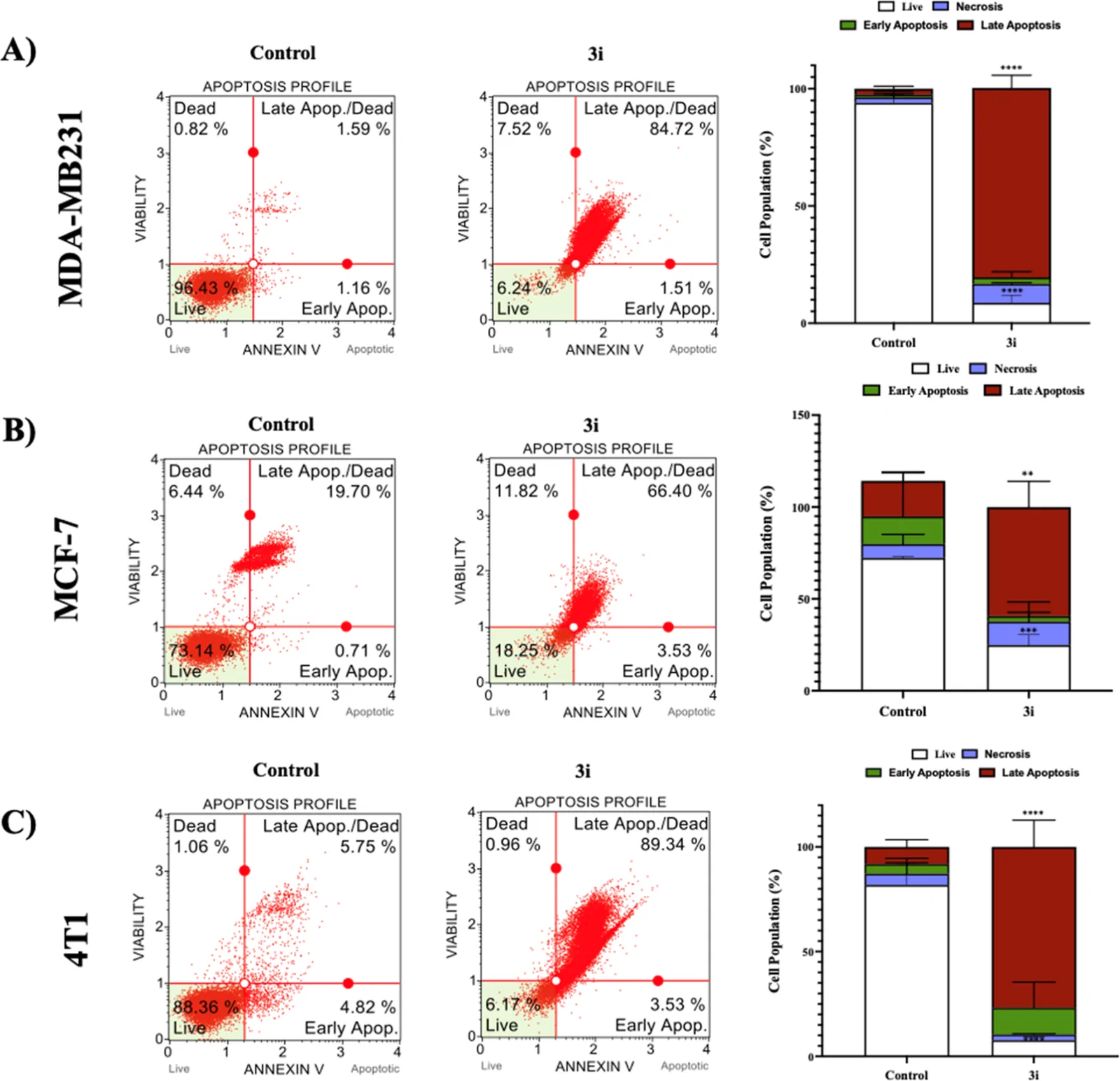
Effect of **3i** on breast cancer cell lines, MDA-MB231, MCF-7 and 4T1 following 72 h-treatment with **3i**. (A) MDA-MB231 cells, (B) MCF-7 cells and (C) 4T1 cells. Dot plots representing control and **3i** treated cells. Scatter plot represents the percentages of necrosis (upper left), late apoptosis (upper right), viable cells (lower left), and early apoptosis (lower right) populations of breast cancer cells. (****P ≤ 0.001).

Consistent with its cytotoxic activity, treatment of breast cancer cells with nimesulide derivative **3i** induced a marked apoptotic response. At 40 μg/mL, compound **3i** induced late apoptosis in approximately 75% of breast cancer cells under the present experimental conditions. Nimesulide has also been reported to induce apoptosis in several carcinoma models, including pancreatic, oral, and pharyngeal cancer cell lines.

Overall, these results show that chemical modification of nimesulide scaffold is associated with increased cytotoxic and pro-apoptotic activity and a shift toward late apoptotic cell death which is more pronounced in highly aggressive triple negative breast cancer models.

### 2.4 MetAP-2 Enzyme Activity

MetAP-2 enzyme inhibition screenings were performed for compounds that did and did not show toxicity in HEK293 cells. In this context, MetAP-2 enzyme inhibition was assessed based on the degradation of the fluorescent substrate l-methionine 7-amido-4-methylcoumarin (Met-AMC). The inhibition percentages of MetAP-2 at the 40 μg/mL concentrations of the tested compounds are presented in Table 4. Compounds **3a**, **3i**, and **3j** showed highest level of MetAP-2 inhibition values of 77.57 ± 0.37%, 78.73 ± 1.08%, and 58.70 ± 2.33%, respectively.

**Table 4:** METAP-2 Inhibition of Novel Nimesulide Derivates^a^

comp. no	MetAP-2 enzyme inhibition % (40 μg/mL)
**3a**	77.57 ± 0.37
**3b**	ND
**3c**	ND
**3d**	1.14 ± 0.44
**3e**	0.16 ± 0.12
**3f**	2.32 ± 0.11
**3g**	ND
**3h**	1.88 ± 1.07
**3i**	78.73 ± 1.08
**3j**	58.70 ± 2.33

a ND: not determined.

### 2.5 Molecular Dynamics Simulation

As shown in Table 5, the computational docking results revealed that compound **3a** is a promising candidate with favorable binding energy and inhibition constant. Compound **3a** exhibited a binding energy of −8.1 kcal/mol and a *K*_i_ value of 1.14 μM, indicating strong potential to effectively bind and inhibit the MetAP2 enzyme Table 5.

**Table 5:** Compound IDs, R Groups, 2D Structure Representations, Calculated Binding Free Energies (Δ*G*, kcal/mol), and Inhibition Constants (*K*_i_, μM) of the Compounds from **3a**–**j**

	MetAP2 protein	
comp. no	free energy of binding (kcal/mol)	inhibition constant (*K*_i_, μM)
**3a**	–8.1	1.14
**3b**	–7.6	2.66
**3c**	–8.1	1.14
**3d**	–8.1	1.14
**3e**	–8.7	0.416
**3f**	–8.6	0.491
**3g**	–8.3	0.815
**3h**	–8.3	0.815
**3i**	–8.2	0.965
**3j**	–8.5	0.581

**3a** was docked into the MetAP2 active site (Figure 8). The compound forms various interactions with the critical amino acid side chain in the catalytic cavity, including van der Waals interactions, conventional hydrogen bonds, carbon–hydrogen bonds, π-sulfur interactions, π–π stacking, π–π T-shaped interactions, alkyl interactions, and π-Alkyl interactions. Two crucial traditional hydrogen bonds form with the nitrogen atom in the amide bond and the residues ASN329 and GLU364. Additionally, one hydrogen bond occurs between the carbonyl moiety of the compound **3a** and HIS231 of the site chain of the active side. Two more hydrogen bonds form between the sulfonate of the compound **3a** and HIS231 and GLY222. Considering all these strong attractions, this compound is a very potent MetAP2 inhibitor, in agreement with the experimental results. Compound **3i** has a computational free energy of binding of Δ*G* = −8.2 kcal/mol and an inhibition constant of 0.97 μM.

**Figure 8:**
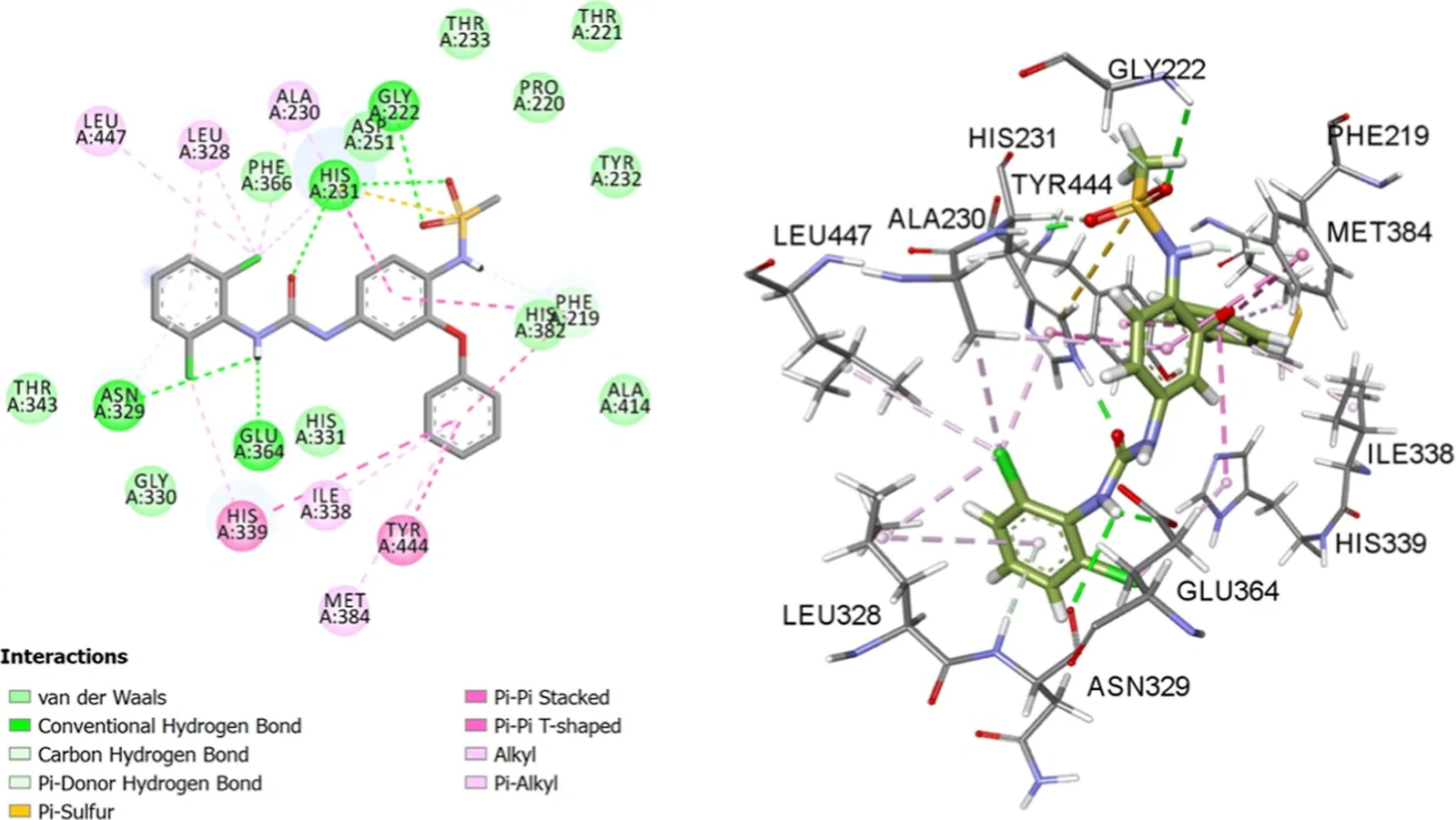
2D (The types of nonbonded interactions are indicated as respective colors in the 2D scheme) and 3D pictures of the docked compound **3a**.

Following this, RMSD and RMSF analysis of the molecular dynamics simulation studies show the consistent stability of these systems throughout the 200 ns simulation (Figure 9). RMSD and RMSF graphs were the deterministic hallmarks of interpreting the enzyme-ligand interactions and residue fluctuations. Stabilities of the protein and ligand complexes throughout the simulation were monitored by visualizing the trajectories of systems along a 200 ns simulation. The MetAP2–3a complex exhibited a smooth and stable path throughout the simulation, maintaining between 1.6 Å and 2.4 Å, as shown in Figure 9. The RMSF plot also indicated that the residues that fluctuate more are in the loop regions of the enzyme depicted in Figure 9.

**Figure 9:**
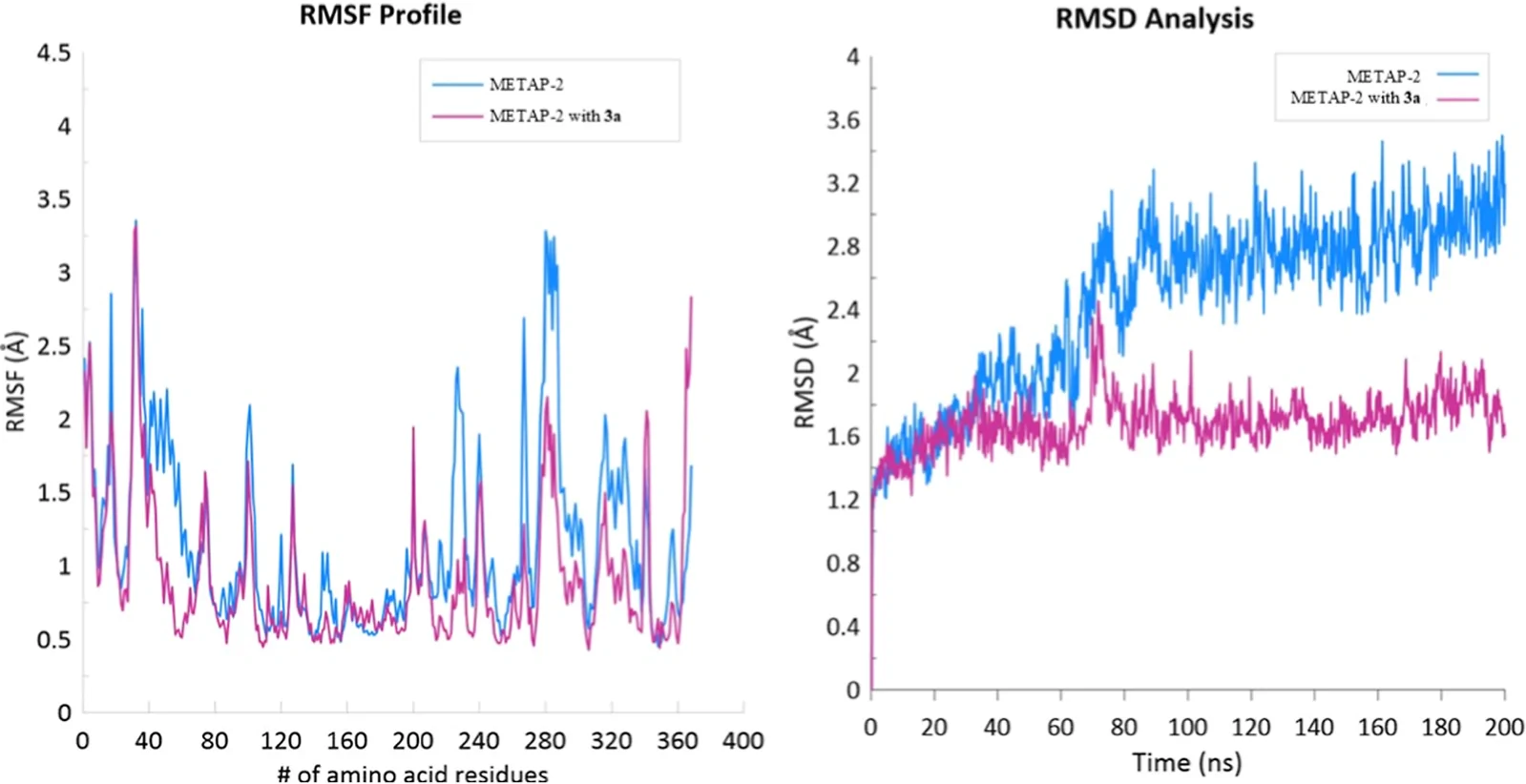
RMSD (up) and RMSF (down) profiles of the MetAP2-**3a** complex (red) and the MetAP2 alone (blue) after exposure to a 200 ns simulation.

The binding free energy (Δ*G*) derived from MM/GBSA analysis plays a pivotal role in assessing ligand performance. When interpreted alongside experimental inhibition measurements (IC_50_), these computational findings offer valuable insight into the potential of the compounds as effective enzyme inhibitors. Lower (more negative) Δ*G* values reflect stronger ligand–protein interactions, often indicating increased binding stability and enhanced inhibitory capability. Figure 10 presents the MM/GBSA-derived binding free energies for the 12 evaluated compounds (**3a–j**), reported as their corresponding average Δ*G* values.

**Figure 10:**
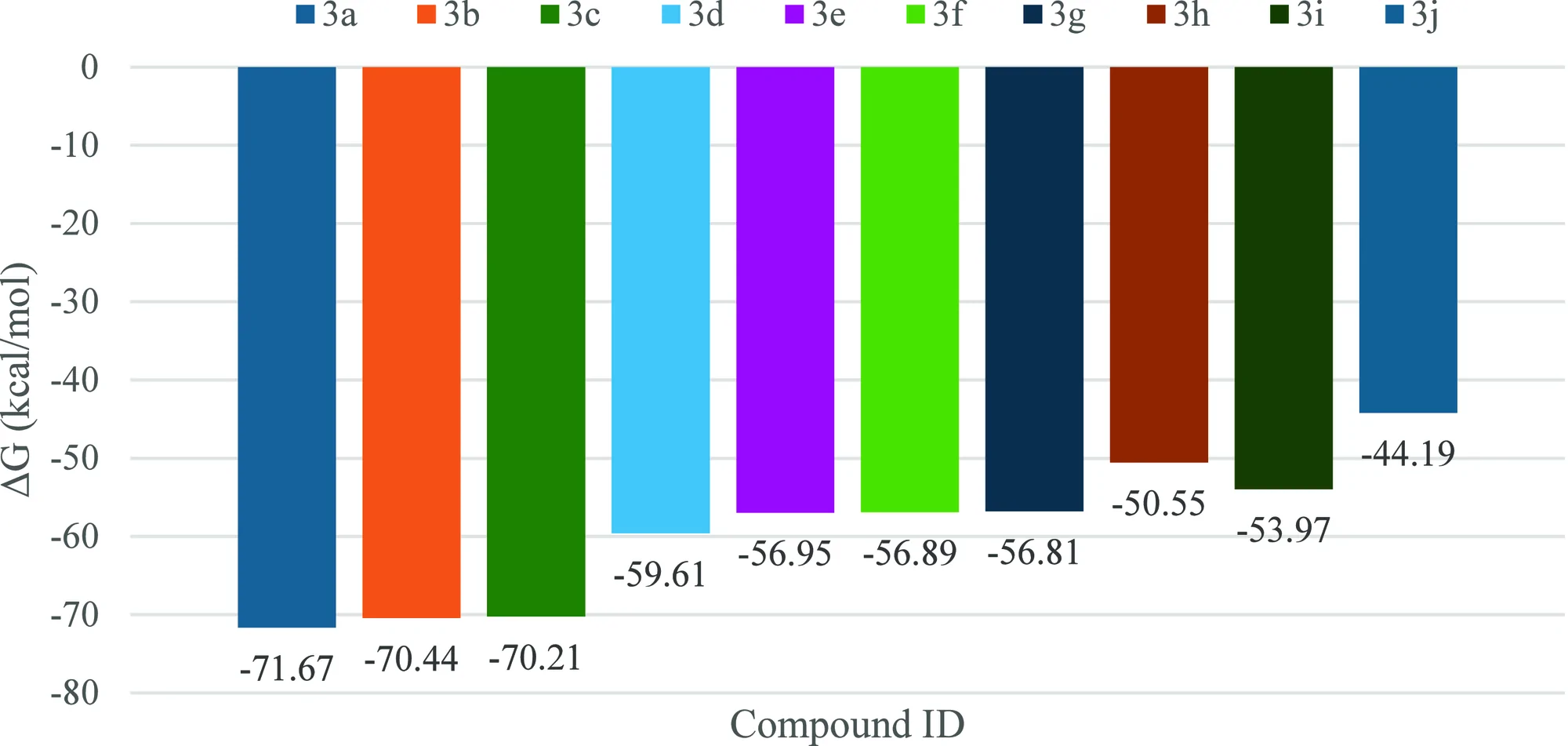
Mean MM/GBSA binding free energies for compounds **3a–j** were determined based on their interactions with the MetAP2 enzyme over the course of the 200 ns molecular dynamics simulations.

Advanced molecular dynamics evaluations demonstrated that ligands **3a** and **3i** promote a pronounced stabilization of the MetAP2 enzyme, as evidenced by their consistently lower RMSD and RMSF profiles throughout the simulation trajectory. This reduced structural fluctuation indicates a more rigid and durable ligand–enzyme complex, suggesting favorable binding persistence and conformational stability. These computational observations position **3a** and **3i** as promising MetAP2 inhibitors; however, comprehensive in vivo studies are required to substantiate their inhibitory efficacy and to further explore their therapeutic potential as anticancer candidates targeting MetAP2.^38–43^

## 3 Conclusion

Nonsteroidal anti-inflammatory drugs (NSAIDs) have been widely reported to exhibit antiproliferative effects against various cancer types. Among these agents, nimesulide has attracted attention due to its reported biological activity, including growth-inhibitory effects on cancer cells. A series of compounds (**3a–j**) were synthesized via organotin-catalyzed structural modification using various isocyanates, affording nimesulide derivatives. The structures of the synthesized compounds (**3a–j**) were confirmed by comprehensive spectroscopic analyses, including ^1^H NMR, ^13^C NMR, FTIR, and MS.

The antioxidant capacities of urea derivatives have been widely highlighted in previous studies. In this context, the newly synthesized nimesulide-based urea compounds (**3a–j**) were assessed using the DPPH (2,2-diphenyl-1-picrylhydrazyl) radical scavenging assay. The results demonstrated that compounds **3e**, **3f**, and **3i** exhibited markedly high antioxidant activity, comparable to the reference standards. Notably, all three of these derivatives contain a fluorine-substituted moiety, suggesting that the presence of fluorine may play a contributory role in enhancing radical scavenging efficiency.

Moreover, the nimesulide derivative **3i** demonstrated a markedly enhanced *in vitro* anticancer profile across multiple breast cancer cell lines, exhibiting cytotoxicity and a pronounced shift toward late-stage apoptosis. These findings indicate that rational structural modification of the nimesulide scaffold can substantially improve its anticancer efficacy. The robust activity of **3i**, particularly in aggressive triple-negative breast cancer models, highlights its promise as a lead candidate for further mechanistic studies. Taken together, the combined cytotoxic and apoptosis-promoting properties of compound **3i** is a promising NSAID-derived anticancer candidate. Consistent with its antiproliferative and pro-apoptotic properties, compound **3i** also exhibited inhibitory activity against the MetAP2 enzyme, supporting its consideration as a nimesulide-based candidate. Parallel computational studies, including molecular docking and molecular dynamics simulation results, further supported the experimental IC50 data for nimesulide derivatives. In summary, these findings provide a crucial basis for developing new strategies in cancer treatment.

## 4 Materials and Methods

### 4.1 Chemistry

All chemicals and solvents employed in the synthetic and in vitro procedures were procured from reputable commercial sources, including Merck, Sigma-Aldrich, Acros Organics, and Thermo Fisher Scientific, and were utilized as received without additional purification. Purification of the target compounds was achieved via column chromatography on silica gel (particle size range: 0.063–0.200 mm). The reaction progress was routinely examined through thin-layer chromatography (TLC) employing a hexane/ethyl acetate mixture as the mobile phase at 25 °C, and visualized under ultraviolet light (254 nm). Melting point determinations were carried out using a Büchi Stuart SMP20 digital apparatus. The ^1^H and ^13^C nuclear magnetic resonance (NMR) spectra were acquired on a Varian Premium Shielded spectrometer operating at 600 and 150 MHz, respectively, with DMSO-*d*_6_ serving as the solvent and tetramethylsilane (TMS) as the internal standard. Fourier-transform infrared (FT-IR) spectra were recorded on a PerkinElmer Spectrum BX instrument (Massachusetts, USA). High-resolution mass spectrometric (HR-MS) analyses were performed on an Agilent 6530 Q-TOF system equipped with an electrospray ionization (ESI) source.

#### 4.1.1 Preparation of *N*-(4-Amino-2-phenoxy-phenyl)-methanesulfonamide (**2**)^11,14,15^

Nimesulide (19.46 mmol) was dissolved in anhydrous methanol (150 mL) under a nitrogen atmosphere. Separately, a solution of SnCl_2_ (87.02 mmol) in concentrated hydrochloric acid (30 mL) was prepared and then slowly added dropwise to the nimesulide solution within approximately 10 min. The reaction mixture was heated under reflux for 3 h, ensuring continuous stirring throughout. After completion, the methanol was evaporated under reduced pressure using a rotary evaporator. The residue obtained was treated with ethyl acetate, and the organic layer was neutralized with 1 M NaOH followed by washing with distilled water to remove any remaining inorganic salts. The organic phase was then dried over anhydrous sodium sulfate (Na_2_SO_4_), filtered, and the solvent was removed under vacuum. Finally, the crude product was purified by recrystallization from ethanol, yielding the desired compound in a pure form.

##### 4.1.1.1 *N-(4-Amino-2-phenoxy-phenyl)-methanesulfonamide* (**2**)^15^

White powder, yield 68%, mp 166–168 °C, FT-IR υ_max_ (cm^–1^): 3397 and 3330 (N–H), 3015 (aromatic ring, =C–H), 2917 (alkyl, C–H), 1583 and 1506 (aryl, C=C), 1487, 1321 (C–S), 1211 and 1150 (C–N), 757. ^1^H NMR (600 MHz, DMSO-*d*_6_; δ, ppm): 2.86 (S–C **H**_3_, s, 3H), 5.26 (Ar–N **H**_2_, s, 2H), 6.02 (Ar–C **H**, d, *J* = 2.4 Hz, 1H), 6.27 (Ar–C **H**, dd, *J* = 8.5, 2.4 Hz, 1H), 6.97 (Ar–C **H**, d, *J* = 8.5 Hz, 1H), 7.04 (2xAr–C **H**, d, *J* = 7.8 Hz, 2H), 7.14 (Ar–C **H**, t, *J* = 7.4 Hz, 1H), 7.40 (2xAr–C **H**, m, 2H), 8.75 (Ar–N **H**–S, s, 1H). ^13^C NMR (126 MHz, DMSO-*d*_6_; δ, ppm): 40.00 (CH_3_), 102.78 (Ar–**C**), 108.81 (Ar–**C**), 114.78 (Ar–**C**), 119.29 (Ar–**C**), 123.53 (Ar–**C**), 129.89 (Ar–**C**), 130.50 (Ar–**C**), 149.11 (Ar–**C**), 153.14 (Ar–**C**), 156.19 (Ar–**C**). HRMS (EI^+^) (*m*/*z*): 279.08 [M + H]; calcd for C_13_H_14_N_2_SO_3_, 279.07 [M + H]^+^.

#### 4.1.2 General Procedure for the Preparation of Nimesulide Ureas (**3a**–**j**)^15^

A mixture of substituted isocyanates (1.5 mmol) and dibutyltin dilaurate (1.5 mmol) was added to a solution of compound **2** (1 mmol) in tetrahydrofuran (5 mL) under a nitrogen atmosphere. The reaction was maintained under reflux conditions with continuous stirring for 12–18 h. After completion of the reaction, the solvent was evaporated under reduced pressure, and the resulting residue was purified by column chromatography.

##### 4.1.2.1 *N*-[4-[3-(2,6-Dichloro-phenyl)-ureido]-2-phenoxy-phenyl]-methanesulfonamide (**3a**)

White powder, yield 76%, mp 203.5 °C, FT-IR υ_max_ (cm^–1^): 3251 (N–H), 3078 (aromatic ring, =C–H), 2930 (Alkyl, C–H), 1657 (C=O), 1589, 1559, and 1552 (aryl, C=C), 1487, 1321 (S=O), 1209 (C–O), 1144 (C–N), 773, 751. ^1^H NMR (600 MHz, DMSO-*d*_6_; δ, ppm): 2.93 (S–C **H**_3_, s, 3H), 7.07 (2x Ar–C **H**, d, *J* = 7.8 Hz, 2H), 7.12 (Ar–C **H**, dd, *J* = 8.8, 1.7 Hz 1H), 7.16 (2xAr–C **H**, dd, *J* = 9.5, 4.7 Hz, 2H), 7.25–7.33 (2xAr–C **H**, m, 2H), 7.40 (2xAr–C **H**, t, *J* = 7.9 Hz, 2H), 7.51 (2xAr–C **H**, t, *J* = 8.8 Hz, 2H), 8.17 (Ar–N **H**–CO, s, 1H), 9.17 (Ar–N **H**–CO ve Ar–N **H**–S, d, *J* = 14.6 Hz, 2H). ^13^C NMR (150 MHz, DMSO-*d*_6_; δ, ppm): 40.09 (CH_3_), 107.49 (Ar–**C**), 112.77 (Ar–**C**), 119.08 (Ar–**C**), 123.70 (Ar–**C**), 128.29 (Ar–**C**), 129.87 (Ar–**C**), 132.99 (Ar–**C**), 133.89 (Ar–**C**), 138.99 (Ar–**C**), 151.35 (Ar–**C**), 152.03 (Ar–**C**), 155.83 (NH–C=**O**). HRMS (EI^+^) (*m*/*z*): 466.04 [M + H]; calcd for C_20_H_17_N_3_SO_4_Cl_2_, 466.03 [M + H]^+^.

##### 4.1.2.2 *N*-[4-[3-(3-Chloro-phenyl)-ureido]-2-phenoxy-phenyl]-methanesulfonamide (**3b**)

White powder, yield 81%, mp 193.2 °C, FT-IR υ_max_ (cm^–1^): 3257 (N–H), 3089 (aromatic ring, =C–H), 2969 and 2928 (Alkyl, C–H), 1650 (C=O), 1591 and 1541 (aryl, C=C), 1489, 1325 (S=O), 1218 (C–O), 1146 (C–N), 770, 742. ^1^H NMR (600 MHz, DMSO-*d*_6_; δ, ppm): 2.91 (S–C **H**_3_, s, 3H), 6.96–6.98 (Ar–**H**, m, 1H), 7.07 (3x Ar–**H**, dd, *J* = 12.0, 5.5 Hz, 3H), 7.12 (Ar–**H**, t, *J* = 2.3 Hz, 1H), 7.14–7.19 (2x Ar–**H**, dd, *J* = 17.4, 8.9 Hz, 2H), 7.22–7.27 (2xAr–**H**, m, 2H), 7.40 (2xAr–**H**, t, *J* = 7.9 Hz, 2H), 7.58–7.59 (Ar–**H**, m, 1H), 8.76 (Ar–N **H**–CO, s, 1H), 8.87 (Ar–N **H**–S, s, 1H), 9.18 (Ar–N **H**–CO, s, 1H). ^13^C NMR (150 MHz, DMSO-*d*_6_; δ, ppm): 43.41 (CH_3_), 111.02 (Ar–**C**), 116.32 (Ar–**C**), 119.84 (Ar–**C**), 120.68 (Ar–**C**), 122.25 (Ar–**C**), 124.56 (Ar–**C**), 124.67 (Ar–**C**), 126.96 (Ar–**C**), 131.59 (Ar–**C**), 133.13 (Ar–**C**), 133.47 (Ar–**C**), 136.22 (Ar–**C**), 141.80 (Ar–**C**), 144.07 (Ar–**C**), 154.49 (Ar–**C**), 155.22 (Ar–**C**), 159.07 (NH–C=**O**). HRMS (EI^+^) (*m*/*z*): 432.08 [M + H]; calcd for C_20_H_18_N_3_SO_4_Cl, 432.07 [M + H]^+^.

##### 4.1.2.3 *N*-{4-[3-(2,4-Dimethoxy-phenyl)-ureido]-2-phenoxy phenyl}­methanesulfonamide (**3c**)

White powder, yield 86%, mp 198.7 °C, FT-IR υ_max_ (cm^–1^): 3272 (N–H), 3096 (aromatic ring, =C–H), 2958 and 2928 (alkyl, C–H), 1648 (C=O), 1556 and 1502 (aryl, C=C), 1487, 1316 (S=O), 1216 and 1203 (C–O), 1153 (C–N), 788, 770. ^1^H NMR (600 MHz, DMSO-*d*_6_; δ, ppm): 2.93 (S–C **H**_3_, s, 3H), 3.71 (OC **H**_3_, s, 3H), 3.83 (OC **H**_3_, s, 3H), 6.43 (Ar–C **H**, dd, *J* = 8.9, 2.7 Hz, 1H), 6.59 (Ar–C **H**, d, *J* = 2.7 Hz, 1H), 7.05–7.12 (4xAr–C **H**, m, 4H), 7.16–7.20 (Ar–C **H**, m, 1H), 7.27 (Ar–C **H**, d, *J* = 8.6 Hz, 1H), 7.41–7.45 (2xAr–C **H**, m, 2H), 7.82 (Ar–C **H**, d, *J* = 8.8 Hz, 1H), 8.09 (Ar–N **H**–CO, s, 1H), 9.15 (Ar–N **H**–S, s, 1H), 9.25 (Ar–N **H**–CO, s, 1H). ^13^C NMR (150 MHz, DMSO-*d*_6_; δ, ppm): 37.61 (CH_3_), 54.63 (O **C** H_3_), 55.19 (O **C** H_3_), 98.10 (Ar–**C**), 103.45 (Ar–**C**), 106.57 (Ar–**C**), 111.91 (Ar–**C**), 118.65 (Ar–**C**), 119.05 (Ar–**C**), 120.23 (Ar–**C**), 120.89 (Ar–**C**), 123.26 (Ar–**C**), 128.10 (Ar–**C**), 128.89 (Ar–**C**), 129.42­(Ar–**C**), 133.13 (Ar–**C**), 138.77 (Ar–**C**), 148.52 (Ar–**C**), 151.01 (Ar–**C**), 151.65 (Ar–**C**), 154.38 (Ar–**C**), 155.36 (Ar–**C**), 164.44 (NH–C=**O**). HRMS (EI^+^) (*m*/*z*): 458.14 [M + H]; calcd for C_22_H_23_N_3_SO_6_, 458.13 [M + H]^+^.

##### 4.1.2.4 *N*-[4-[3-(4-Bromo-phenyl)-ureido]-2-phenoxy-phenyl]-methanesulfonamide (**3d**)

White powder, yield 74%, mp 245.1 °C, FT-IR υ_max_ (cm^–1^): 3314 (N–H), 3054 (aromatic ring, =C–H), 2965 and 2928 (alkyl, C–H), 1635 (C=O), 1591, 1543, and 1506 (aryl, C=C), 1489, 1327 (S=O), 1209 (C–O), 1157 (C–N), 770, 753. ^1^H NMR (600 MHz, DMSO-*d*_6_; δ, ppm): 3.00 (S–C **H**_3_, s, 3H), 7.13–7.15 (3xAr–C **H**, m, 3H), 7.21–7.26 (2xAr–C **H**, m, 2H), 7.35 (Ar–C **H**, d, *J* = 8.7 Hz, 1H), 7.41–7.51 (6xAr–C **H**, m, 6H), 8.78 (Ar–N **H**–CO, d, *J* = 1.9 Hz 1H), 8.90 (Ar–N **H**–S, s, 1H), 9.24 (Ar–N **H**–CO, s, 1H). ^13^C NMR (150 MHz, DMSO-*d*_6_; δ, ppm): 40.78 (CH_3_), 108.29 (Ar–**C**), 113.59 (Ar–**C**), 113.81 (Ar–**C**), 119.68 (Ar–**C**), 120.69 (Ar–**C**), 121.81 (Ar–**C**), 124.35 (Ar–**C**), 128.99 (Ar–**C**), 130.50 (Ar–**C**), 131.95 (Ar–**C**), 139.33 (Ar–**C**), 151.94 (Ar–**C**), 152.62 (Ar–**C**). 156.43 (NH–C=**O**). HRMS (EI^+^) (*m*/*z*): 476.03 [M + H]; calcd for C_20_H_18_N_3_SO_4_Br, 476.02 [M + H]^+^.

##### 4.1.2.5 *N-[2-Phenoxy-4-[3-(3-trifluoromethyl-phenyl)-ureido]-phenyl]-methanesulfonamide* (**3e**)

White powder, yield 86%, mp 163.9 °C, FT-IR υ_max_ (cm^–1^): 3318 and 3259 (N–H), 3061 (aromatic ring, =C–H), 2930 (alkyl, C–H), 1650 (C=O), 1611 and 1550 (aryl, C=C), 1489, 1321 (S=O), 1218 (C–O), 1111 (C–N), 1065 (C–F), 792, 746. ^1^H NMR (600 MHz, DMSO-*d*_6_; δ, ppm): 2.94 (S–C **H**_3_, s, 3H), 7.08 (2x Ar–C **H**, d, *J* = 8.2 Hz, 2H), 7.13 (2x Ar–C **H**, m, 2H), 7.18 (Ar–C **H**, t, *J* = 7.4 Hz, 1H), 7.30 (2x Ar–C **H**, dd, *J* = 7.9, 4.3 Hz, 2H), 7.43 (2xAr–C **H**, t, *J* = 7.8 Hz, 2H), 7.48 (Ar–C **H**, t, *J* = 7.9 Hz, 1H), 7.53 (Ar–C **H**, d, *J* = 8.3 Hz, 1H), 7.90 (Ar–C **H**, s, 1H), 8.94 (Ar–N **H**–CO ve Ar–N **H**–S, d, *J* = 3.7 Hz, 2H), 9.21 (Ar–N **H**–CO, s, 1H). ^13^C NMR (150 MHz, DMSO-*d*_6_; δ, ppm): 40.35 (CH_3_), 108.12 (Ar–**C**), 113.41 (Ar–**C**), 114.17 (Ar–**C**), 118.23 (Ar–C **F**_**3**_), 119.11 (Ar–**C**), 119.32 (Ar–**C**), 121.63 (Ar–**C**), 121.99 (Ar–**C**), 123.85 (Ar–**C**), 128.47 (Ar–**C**), 129.91 (Ar–**C**), 130.04 (Ar–**C**), 138.66 (Ar–**C**), 140.33 (Ar–**C**), 151.33 (Ar–**C**), 152.28 (Ar–**C**), 156.04 (NH–C=**O**). HRMS (EI^+^) (*m*/*z*): 466.10929 [M + H]; calcd for C_21_H_18_N_3_SO_4_F_3_, 466.10 [M + H]^+^.

##### 4.1.2.6 *N*-[4-[3-(4-Fluoro-benzyl)-ureido]-2-phenoxy-phenyl]-methanesulfonamide (**3f**)

White powder, yield 68%, mp 168.4 °C, FT-IR υ_max_ (cm^–1^): 3410 and 3334 (N–H), 3074 (aromatic ring, =C–H), 2969 and 2928 (alkyl, C–H), 1661 (C=O), 1554 and 1506 (aryl, C=C), 1487, 1327 (S=O), 1207 (C–O), 1142 (C–N), 755. ^1^H NMR (600 MHz, DMSO-*d*_6_; δ, ppm): 2.97 (S–C **H**_3_, s, 3H), 4.27 (C **H**_2_, d, *J* = 5.9 Hz, 2H), 6.62 (Ar–CO–N **H**–CH_2_, t, *J* = 6.0 Hz 1H), 7.09–7.12 (3xAr–C **H**, m, 3H), 7.16–7.24 (4xAr–C **H**, m, 4H), 7.28 (Ar–C **H**, d, *J* = 8.7 Hz, 1H), 7.32–7.34 (2xAr–C **H**, m, 2H), 7.45–7.49 (2xAr–C **H**, m, 2H), 8.78 (Ar–N **H**–S, s, 1H), 9.17 (Ar–N **H**–CO, s, 1H). ^13^C NMR (150 MHz, DMSO-*d*_6_; δ, ppm): 40.70 (CH_3_), 42.38 (CH_2_), 107.80 (Ar–**C**), 113.05 (Ar–**C**), 115.53 (Ar–**C**), 115.54 (Ar–**C**), 119.59 (Ar–**C**), 120.99 (Ar–**C**), 124.21 (Ar–**C**), 129.11 (Ar–**C**), 129.42 (Ar–**C**), 129.50 (Ar–**C**), 130.45 **(**Ar–**C**), 136.95 **(**Ar–**C**), 140.37 **(**Ar–**C**), 152.00 **(**Ar–**C**), 155.36 **(**Ar–**C**), 156.54 **(**Ar–**C**), 160.36 **(**Ar–**C**), 162.76 **(**NH–C=**O**). HRMS (EI^+^) (*m*/*z*): 430.12 [M + H]; calcd for C_21_H_20_N_3_SO_4_F, 430.12 [M + H]^+^.

##### 4.1.2.7 *N*-[4-[3-(3,5-Dichloro-phenyl)-ureido]-2-phenoxy-phenyl]-methanesulfonamide (**3g**)

White powder, yield 82%, mp 209.7 °C, FT-IR υ_max_ (cm^–1^): 3325 (N–H), 3093 (aromatic ring, =C–H), 2982 and 2928 (alkyl, C–H), 1589 (C=O), 1537 and 1505 (aryl, C=C), 1487, 1316 (S=O), 1205 (C–O), 1158 and 1109 (C–N), 755, 692 (C–Cl). ^1^H NMR (600 MHz, DMSO-*d*_6_; δ, ppm): 2.95 (S–C **H**_3_, s, 3H), 7.07–7.21 (6x Ar–C **H**, m, 6H), 7.31 (Ar–C **H**, d, *J* = 8.8 Hz, 1H), 7.41–7.46 (4x Ar–C **H**, m, 4H), 8.96 (Ar–N **H**–CO, s, 1H), 9.03 (Ar–N **H**– S, s, 1H), 9.21 (Ar–N **H**–CO, s, 1H). ^13^C NMR (150 MHz, DMSO-*d*_6_; δ, ppm): 39.73 **(** CH_3_), 107.59 **(**Ar–**C**), 112.87 **(**Ar–**C**), 115.79 **(**Ar–**C**), 118.52 **(**Ar–**C**), 120.41 **(**Ar–**C**), 121.19 **(**Ar–**C**), 123.26 **(**Ar–**C**), 127.78 **(**Ar–**C**), 129.43 **(**Ar–**C**), 133.43 **(**Ar–**C**), 137.80 **(**Ar–**C**), 141.41 **(**Ar–**C**), 150.70 **(**Ar–**C**), 151.40 **(**Ar–**C**), 155.39 **(**NH–C=**O**). HRMS (EI^+^) (*m*/*z*): 466.04 [M + H]; calcd for C_20_H_17_N_3_SO_4_Cl_2_, 466.03 [M + H]^+^.

##### 4.1.2.8 *N*-[4-[3-(3-Chloro-4-methyl-phenyl)-ureido]-2-phenoxy-phenyl]-methanesulfonamide (**3h**)

White powder, yield 88%, mp 228.2 °C, FT-IR υ_max_ (cm^–1^): 3270 (N–H), 3091 (aromatic ring, =C–H), 2928 (alkyl, C–H), 1657 (C=O), 1609, 1550, and 1506 (aryl, C=C), 1489, 1323 (S=O), 1222 (C–O), 1142 (C–N), 751, 692 (C–Cl). ^1^H NMR (600 MHz, DMSO-*d*_6_; δ, ppm): 2.30 (Ar–C **H**_3_, s, 3H), 3.00 (S–C **H**_3_, s, 3H), 6.96–6.98 (Ar–**H**, m, 1H), 7.13–7.28 (7xAr–**H**, m, 7H), 7.35 (Ar–**H**, d, *J* = 8.6 Hz, 1H), 7.47–7.51 (2xAr–**H**, dd, *J* = 8.4, 7.5 Hz, 2H), 7.65 (Ar–**H**, d, *J* = 2.1 Hz, 1H), 8.73 (Ar–N **H**–CO, s, 1H), 8.91 (Ar–N **H**–S, s, 1H), 9.24 (Ar–N **H**–CO, s, 1H). ^13^C NMR (150 MHz, DMSO-*d*_6_; δ, ppm): 19.27 **(** CH_3_), 40.78 **(** CH_3_), 108.38 **(**Ar–**C**), 113.67 **(**Ar–**C**), 117.54 **(**Ar–**C**), 118.64 **(**Ar–**C**), 119.60 **(**Ar–**C**), 121.86 **(**Ar–**C**), 124.31 **(**Ar–**C**), 128.85 **(**Ar–**C**), 128.97 **(**Ar–**C**), 130.50 **(**Ar–**C**), 131.61 **(**Ar–**C**), 133.52 **(**Ar–**C**), 139.08 **(**Ar–**C**), 139.31 **(**Ar–**C**), 151.86 **(**Ar–**C**), 152.67 **(**Ar–**C**), 156.49 **(**NH–C=**O**). HRMS (EI^+^) (*m*/*z*): 446.09 [M + H]; calcd for C_21_H_20_N_3_SO_4_Cl, 446.09 [M + H]^+^.

##### 4.1.2.9 *N*-[4-[3-(2,4-Difluoro-phenyl)-ureido]-2-phenoxy-phenyl]-methanesulfonamide (**3i**)

White powder, yield 78%, mp 193.2 °C, FT-IR υ_max_ (cm^–1^): 3270 (N–H), 3093 (aromatic ring, =C–H), 2925 (alkyl, C–H), 1659 (C=O), 1550 and 1500 (aryl, C=C), 1487, 1325 (S=O), 1220 (C–O), 1142 (C–N), 749. ^1^H NMR (600 MHz, DMSO-*d*_6_; δ, ppm): 2.95 (S–C **H**_3_, s, 3H), 6.98–7.04 (Ar–C **H**, m, 1H), 7.06–7.11 (3xAr–C **H**, m, 3H), 7.14 (Ar–C **H**, d, *J* = 2.3 Hz, 1H), 7.17–7.21 (Ar–C **H**, m, 1H), 7.26–7.32 (2xAr–C **H**, m, 2H), 7.41–7.46 (2xAr–C **H**, m, 2H), 7.94–8.00 (Ar–C **H**, m, 1H), 8.39 (Ar–N **H**–CO, d, *J* = 1.9 Hz 1H), 9.13 (Ar–N **H**–S, s, 1H), 9.20 (Ar–N **H**–CO, s, 1H). ^13^C NMR (150 MHz, DMSO-*d*_6_; δ, ppm): 40.77 **(** CH_3_), 103.99 **(**Ar–**C**), 104.26 **(**Ar–**C**), 104.50 **(**Ar–**C**), 108.01 **(**Ar–**C**), 111.40 **(**Ar–**C**), 111.62 **(**Ar–**C**), 113.34 **(**Ar–**C**), 119.76 **(**Ar–**C**), 121.86 **(**Ar–**C**), 124.41 **(**Ar–**C**), 129.05 **(**Ar–**C**), 130.51 **(**Ar–**C**), 139.16 **(**Ar–**C**), 152.02 **(**Ar–**C**), 152.53 **(**Ar–**C**), 156.37 **(**NH–C=**O**). HRMS (EI^+^) (*m*/*z*): 434.10­[M + H]; calcd for C_20_H_17_N_3_SO_4_F_2_, 434.09 [M + H]^+^.

##### 4.1.2.10 *N*-[2-Phenoxy-4-[3-(2-trifluoromethyl-phenyl)-ureido]-phenyl]-methanesulfonamide (**3j**)

White powder, yield 78%, mp 171.2 °C, FT-IR υ_max_ (cm^–1^): 3264 (N–H), 3040 (aromatic ring, =C–H), 2923 (alkyl, C–H), 1639 (C=O), 1589 and 1552 (aryl, C=C), 1488, 1319 (S=O), 1205 (C–O), 1108 (C–N), 1034 (C–F), 760, 771. ^1^H NMR (600 MHz, DMSO-*d*_6_; δ, ppm): 2.91 (S–C **H**_3_, s, 3H), 7.08 (4x Ar–C **H**, dd, *J* = 12.0, 5.4 Hz, 4H), 7.15 (Ar–C **H**, t, *J* = 7.4 Hz, 1H), 7.23 (Ar–C **H**, t, *J* = 7.6 Hz, 1H), 7.27 (Ar–C **H**, t, *J* = 9.3 Hz, 1H), 7.40 (2xAr–C **H**, t, *J* = 7.9 Hz, 2H), 7.57 (Ar–C **H**, t, *J* = 7.8 Hz, 1H), 7.63 (Ar–C **H**, d, *J* = 7.8 Hz, 1H), 7.82 (Ar–C **H**, d, *J* = 8.3 Hz, 1H), 7.96 (Ar–N **H**–CO, s, 1H), 9.18 (Ar–N **H**–S, s, 1H), 9.43 (Ar–N **H**–CO, s, 1H). ^13^C NMR (150 MHz, DMSO-*d*_6_; δ, ppm): 40.27 **(** CH_3_), 107.45 **(**Ar–**C**), 112.81 **(**Ar–**C**), 115.16 **(**Ar–**C**), 116.72 **(**Ar–**C**), 119.39 **(**Ar–**C**), 121.41 **(**Ar–**C**), 123.84 **(**Ar–**C**), 123.98 **(**Ar–**C**), 125.72 **(**Ar–**C**), 128.56 **(**Ar–**C**), 130.06 **(**Ar–**C**), 132.91 **(**Ar–**C**), 136.01 **(**Ar–**C**), 138.72 **(**Ar–**C**), 151.61 **(**Ar–**C**), 152.20 **(**Ar–**C**), 155.84 **(**NH–C=**O**). HRMS (EI^+^) (*m*/*z*): 466.10131 [M + H]; calcd for C_21_H_18_N_3_SO_4_F_3_, 466.10 [M + H]^+^.

### 4.2 Antioxidant Assay

The free radical scavenging potential of the synthesized compounds against DPPH (2,2-diphenyl-1-picrylhydrazyl) was determined according to a modified version of previously described protocols.^2,44–46^ For each compound, a methanolic dilution series was prepared in the concentration range of 250–1000 μM/mL. Subsequently, 0.5 mL of each test solution was mixed with an equal volume (0.5 mL) of a 0.15 mM DPPH solution prepared in methanol. The reaction mixtures were kept in the dark at ambient temperature for 30 min to ensure complete radical scavenging. Absorbance readings were then recorded at 517 nm, with methanol serving as the reference. The DPPH radical scavenging efficiency was quantified using the following formula.

$$DPPH\;radical\;scavenging\;activity\;\left(\%\right)=\left(A_{0}-A_{1}/A_{0}\right)\times 100$$

where *A*_0_ and *A*_1_ are the absorbance of the control (without sample) and the test sample, respectively.

### 4.3 Antiproliferative and Cytotoxic Activity Evaluation

#### 4.3.1 Preparation of Nimesulide Derivatives

Nimesulide derivatives were suspended in dimethyl sulfoxide (DMSO) using the minimum volume required to achieve the highest possible soluble concentration. Stock solutions were prepared such that, after dilution with cell culture medium, the final DMSO concentration did not exceed 0.1% (v/v), corresponding to a 1:1000 dilution. This precaution was taken to avoid potential DMSO-related cytotoxicity and false-positive results. For cytotoxicity assays, all compounds were tested at a maximum final concentration of 40 μg/mL in the culture medium.

#### 4.3.2 WST-1 Cell Viability and Cytotoxicity Assay

The cytotoxic effects of nimesulide derivatives on triple-negative breast cancer cells MDA-MB-231 (5 × 10^3^ cells/well) and HEK293 (5 × 10^3^ cells/well) cells, used as a noncancerous control cell line, were evaluated using the WST-1 cell proliferation assay.^47^ Cells were seeded in 96-well plates in triplicate for each derivative and incubated overnight to allow cell attachment. Following day, cells were treated with vehicle (DMSO) or derivatives (40 μg/mL in the culture medium) for 72 h. At the end of the incubation period, 50 μL of 10% (v/v) WST-1 solution was added to each well and incubated for 1 h. Absorbance was then measured at 450 nm (reference wavelength: 630 nm) using a microplate spectrophotometer (BioTek ELx800, USA). The experiments were performed at least three times independently. Statistical analysis was performed using one-way ANOVA followed by Tukey’s post hoc test. A *p* value ≤0.05 was considered statistically significant.

#### 4.3.3 Cell Death Analysis by Annexin-V Assay

The relative percentages of apoptotic MDA-MB-231, MCF-7, and 4T1 cells in response to 3i nimesulide derivative treatment were determined using the Annexin V-FITC apoptosis detection assay, following the manufacturer’s protocol (Muse Annexin V & Dead Cell Kit, MCH100105). Briefly, 3 × 10^5^ cells/well were seeded in 6-well plates and treated with either 1:1000 diluted DMSO (vehicle control) or **3i** for 72 h. Following the incubation period, the culture medium from each condition was collected, and the wells were washed with 1× PBS. Adherent cells were detached using 0.25% trypsin–EDTA, combined with the previously collected medium, and centrifuged at 350*g* for 5 min. A total of 1 × 10^6^ cells from each sample were stained with the Muse Annexin V & Dead Cell Reagent and incubated for 20 min at room temperature in the dark. Subsequently, 20,000 events per sample were acquired and analyzed using the Guava Muse Cell Analyzer (Merck Millipore, Germany). Experiment was performed at least three times independently. Statistical analysis was performed using one-way ANOVA followed by Tukey’s post hoc test. A *p* value ≤0.05 was considered statistically significant.

### 4.4 MetAP-2 Enzyme Activity Assay

The changes in MetAP-2 enzyme activity induced by the synthesized compounds were determined using a fluorescence-based activity assay as previously described.^47–49^ MetAP-2 enzyme was purchased commercially (APREST74783 Sigma-Aldrich, PrEST Antigen MetAP2) and used at a concentration of 1 μg per reaction. The enzyme was kept in the cold chain until incubations were performed to prevent loss of activity. For the reactions, a buffer containing 50 nM MOPS, pH 7.0, and 50 μM CoCl_2_ was prepared, and 1 μg of MetAP-2 enzyme was added to the 100 μL reaction volume. The synthesized compounds were added to the mixture at their respective 40 μg/mL concentrations (final addition volume: 2 μL) and incubated at room temperature for 15 min. Subsequently, 400 μM l-methionine 7-amido-4-methylcoumarin trifluoroacetate (Met-AMC) was added as the fluorescent substrate to initiate the reaction. The mixtures were incubated at 37 °C for 90 min. Enzyme activity was quantified by measuring fluorescence intensity at 360 nm excitation and 460 nm emission using a fluorescence spectrometer (PerkinElmer, FL 6500).

### 4.5 Molecular Modeling Studies

#### 4.5.1 Protein Structure Setup

The X-ray crystal structure of the hMetAP2 enzyme (PDB ID: 5CLS, resolution 1.75 Å) complexed with a spiroepoxytriazole inhibitor was downloaded from the PDB (https://www.rcsb.org/). The default protein preparation protocol in the Desmond program was carried out, in which all water molecules, native ligands, and salt ions were removed, and hydrogen atoms were added to the enzyme at pH 7.4. Furthermore, a short minimization was applied to the MetAP2 enzyme to correct bond lengths and bond angles. The ChemBioDraw Ultra program was used to prepare ligands for molecular docking of all new compounds, which were saved as SDF files. Then, the default “Prepare Ligands” protocol in BIOVIA DS 4.6 is applied to protonate all ionizable groups.

#### 4.5.2 Docking Setup

All compounds were docked into the active site of the MetAP2 enzyme employing AutoDock 4.2 (https://autodock.scripps.edu). Docking input files were prepared using the Lamarckian genetic algorithm. The grid box dimensions were set to 60 × 60 × 60 Å, encompassing the entire active site of the MetAP2. For the other parameters, the defold setup was adapted for docking.

#### 4.5.3 Molecular Dynamics Simulation

Molecular dynamics simulations were performed to determine the stability of all enzyme-ligand complexes using the OPLS2005 force field in Desmond. The “protein preparation,” “system builder,” and “molecular dynamics” protocols were utilized to run the simulations. The SPC solvent model was used to solvate the system, with boundary conditions set to form an orthorhombic water box with a 10 Å edge length. Then the systems were neutralized by adding calculated amounts of Na^+^ and Cl^–^ ions at 0.15 M. First, the systems were relaxed using Desmond’s default parameters to avoid potential steric clashes. Second, the systems were simulated for 200 ns, yielding 200 trajectories and approximately 1000 frames. The temperature was set to 303.15 K using the *NPT* ensemble, while the pressure was maintained at 1.013 bar throughout the simulation.

## Supplementary Material



## Notes

Experimental details: FT-IR spectra, MS and ^1^H, ^13^C nuclear magnetic resonance spectra of all target products (PDF)
