# Supplementary material for: Organotin-Catalyzed Synthesis, Characterization, Anticancer and Antioxidant Activities, and Molecular Modeling Studies of Nimesulide Ureas as Potential MetAP (Type II) Inhibitors
Source: ACS Omega. 2026 Aug 27;11(35):52473–87. doi: 10.1021/acsomega.6c00645 (PMC13564012; doi:10.1021/acsomega.6c00645)
Supplement: Supplementary file 1 [file ao6c00645_si_001.pdf]

Supporting Information

Organotin-Catalyzed Synthesis, Characterization, Anticancer and Antioxidant Activities, and Molecular Modeling Studies of Nimesulide Ureas as Potential MetAP (type II) Inhibitors

Yağmur Biliz[a,b*], Özgür Yılmaz[b,c*], Elif Kuloğlu[b,d], İpek Bedir[e], Egemen Çakırlı[e], Şevval Arslan[b], Dilek Telci[e], Özge Çevik[f], Kemal Yelekçi[g], Gani Koza[d], Ş. Güniz Küçükgüzel[h]

[a]*Department of Chemical Engineering, Faculty of Engineering and Natural Sciences, Istanbul Health and Technology University, Istanbul 34275, Turkiye*

[b]*TUBITAK Marmara Research Center, Gebze, Kocaeli 41470, Turkiye*

[c]*Department of Pharmaceutical Chemistry, Faculty of Pharmacy, Istanbul University, Istanbul, 34116, Turkiye*

[d]*Department of Chemistry, Faculty of Art and Science, Bursa Uludağ University, Bursa, 16059 Turkiye*

[e]*Department of Genetics and Bioengineering, Yeditepe University, Istanbul, 34755, Turkiye*

[f]*Department of Biochemistry, School of Medicine, Aydın Adnan Menderes University, Aydın, 09010, Turkiye*

[g]*Department of Molecular Biology and Genetics, Faculty of Engineering and Natural Sciences, Kadir Has University, Istanbul 34083, Turkiye*

[h]*Department of Pharmaceutical Chemistry, Faculty of Pharmacy, Fenerbahçe University, Atasehir, Istanbul 34758, Turkiye*

*Corresponding authors. yagmur.biliz@istun.edu.tr, yilmaz.ozgur@tubitak.gov.tr

Contents

FTIR, MS and NMR Spectra of synthesized compound (**2**)..................................... S2

FTIR, MS and NMR Spectra of synthesized compound (**3a-j**)................................. S4

Copies of the FTIR, MS, ^{1}H NMR and ^{13}C NMR spectra of synthesized N-(4-Amino-2-phenoxy-phenyl)-methanesulfonamide (2)

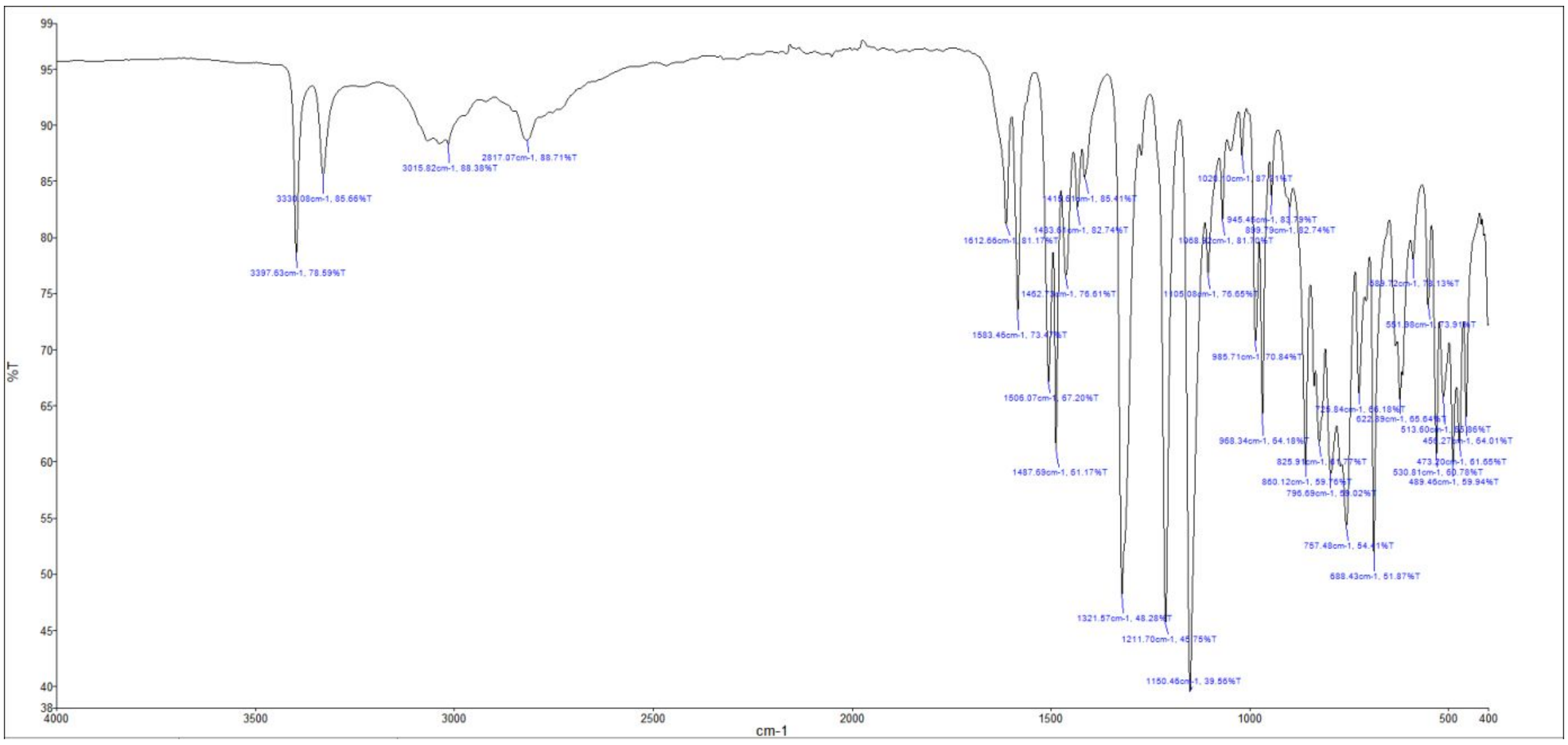

Figure S1. FTIR spectrum of **2**

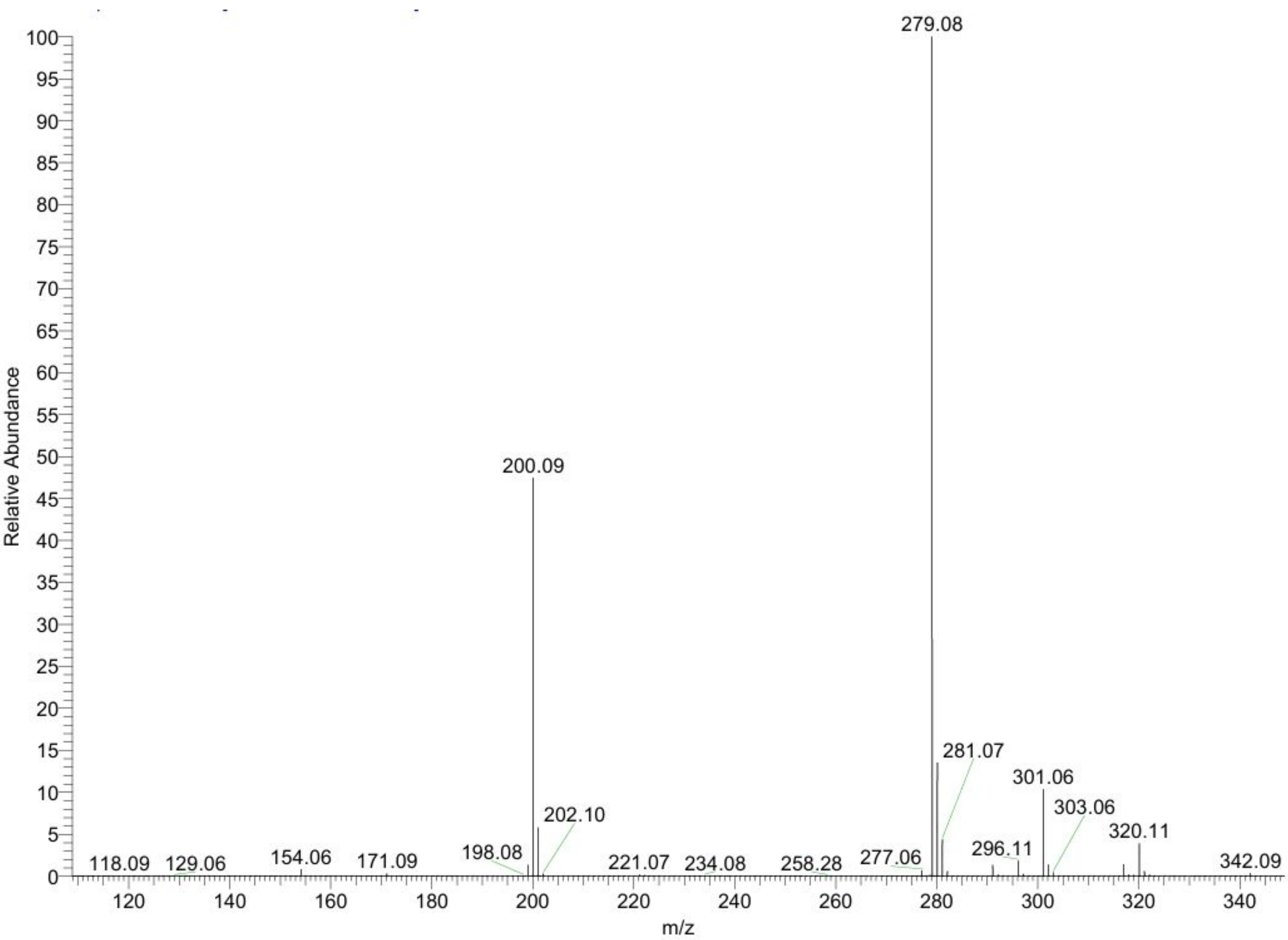

Figure S2. MS spectrum of **2**

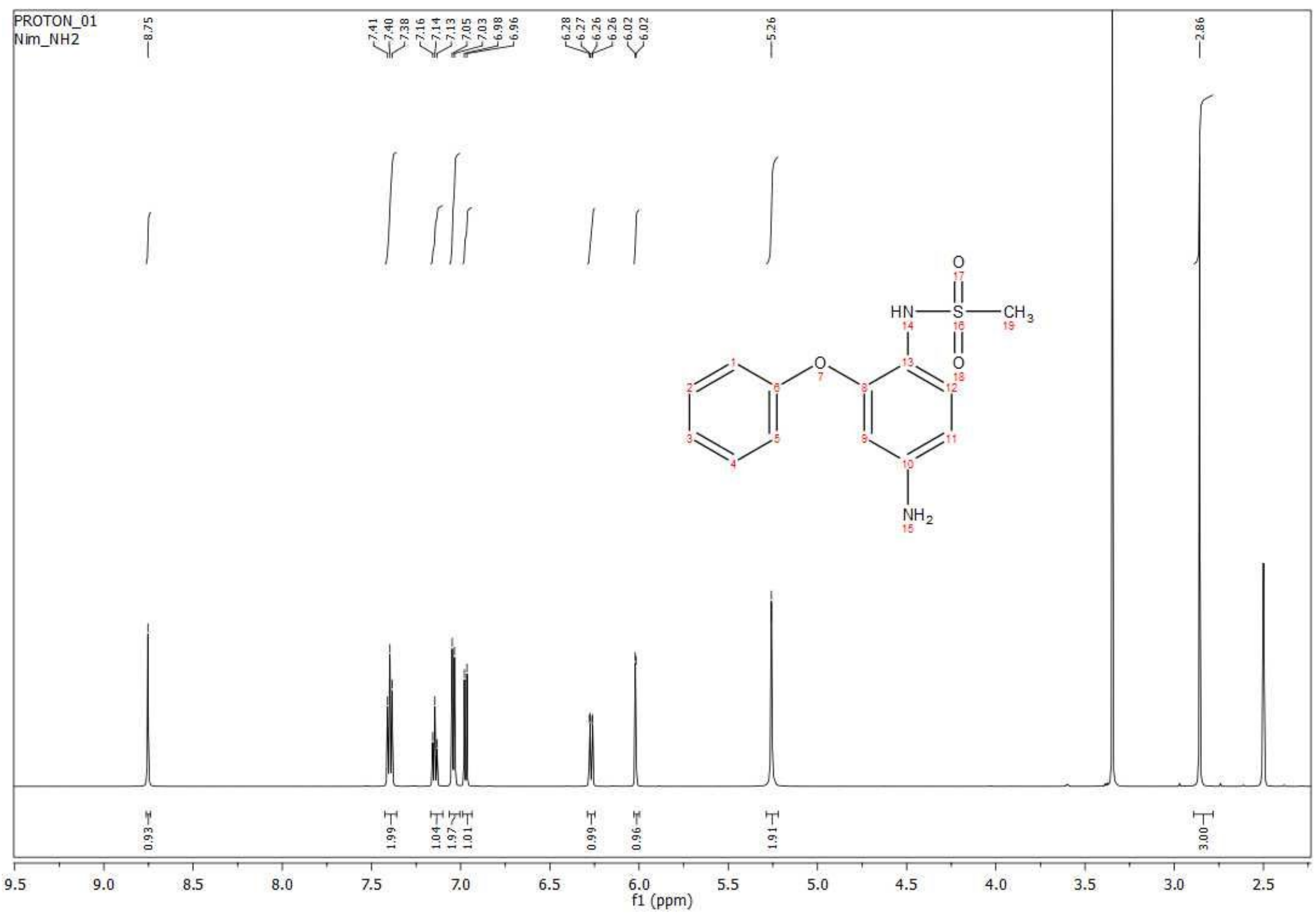

Figure S3. ^{1}H NMR spectrum (600 MHz, DMSO) of **2**

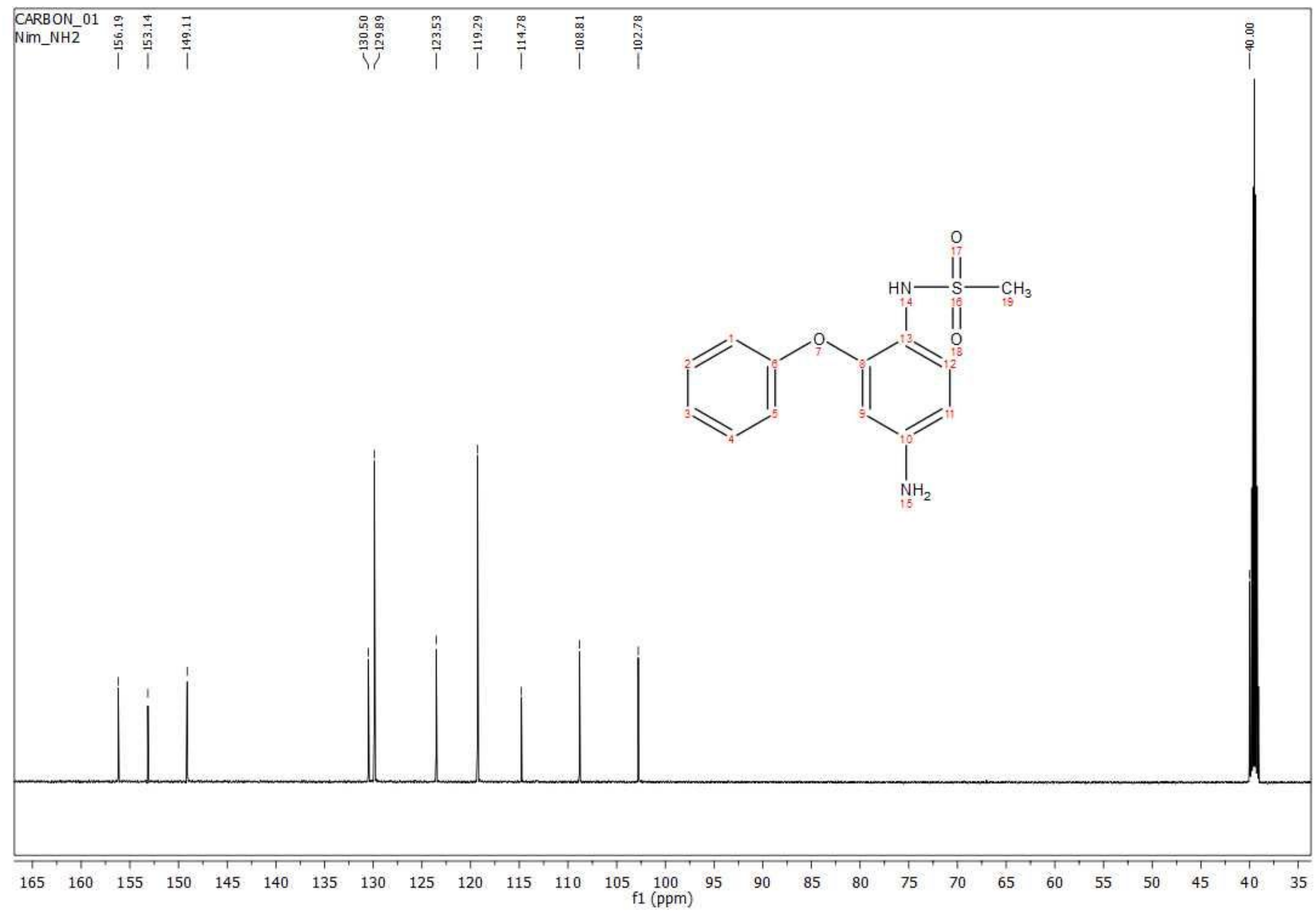

Figure S4. ^{13}C NMR spectrum (150 MHz, DMSO) of **2.**

Copies of the FTIR, MS, ^{1}H NMR and ^{13}C NMR spectra of synthesized N-{4-[3-(2,6-Dichloro-phenyl)-ureido]-2-phenoxy-phenyl}-methanesulfonamide (3a)

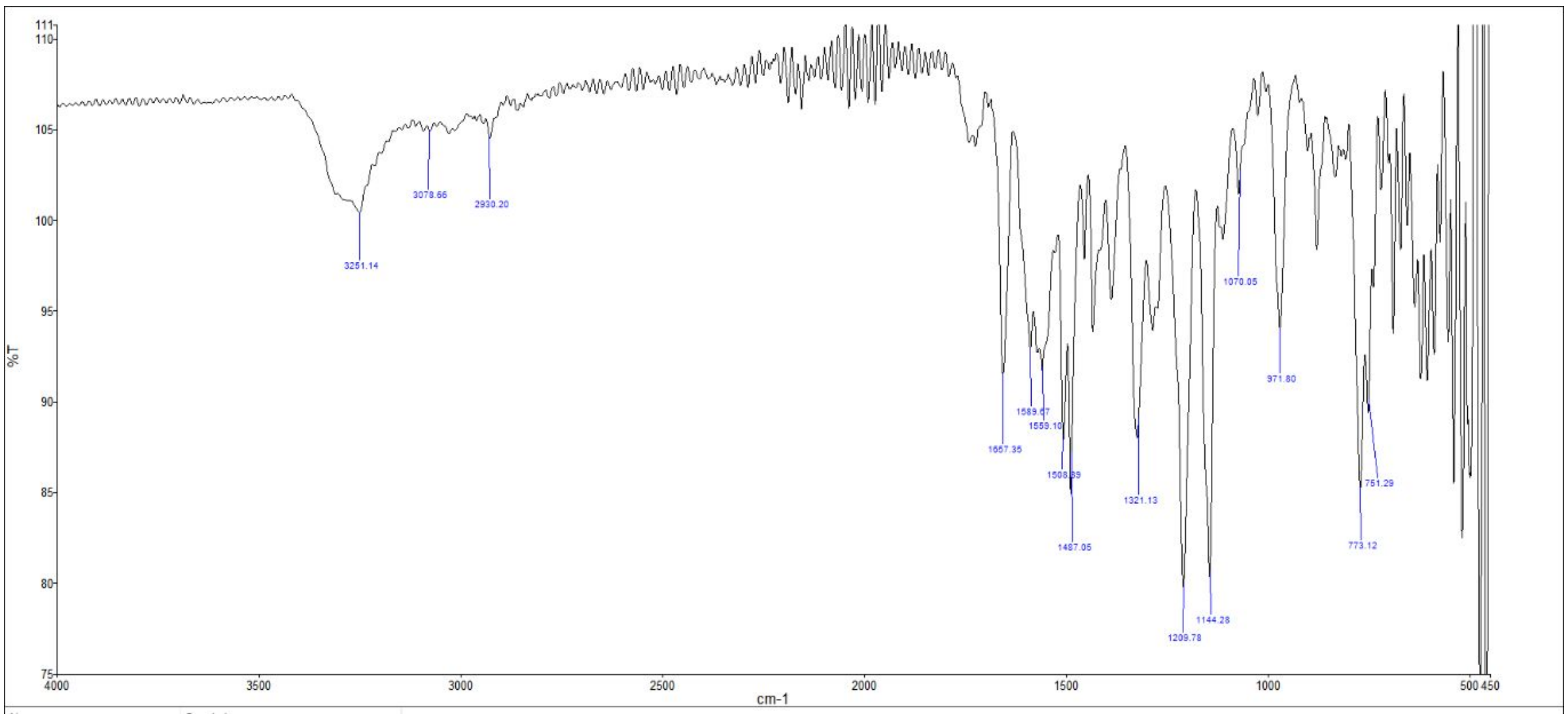

Figure S5. FTIR spectrum of **3a**

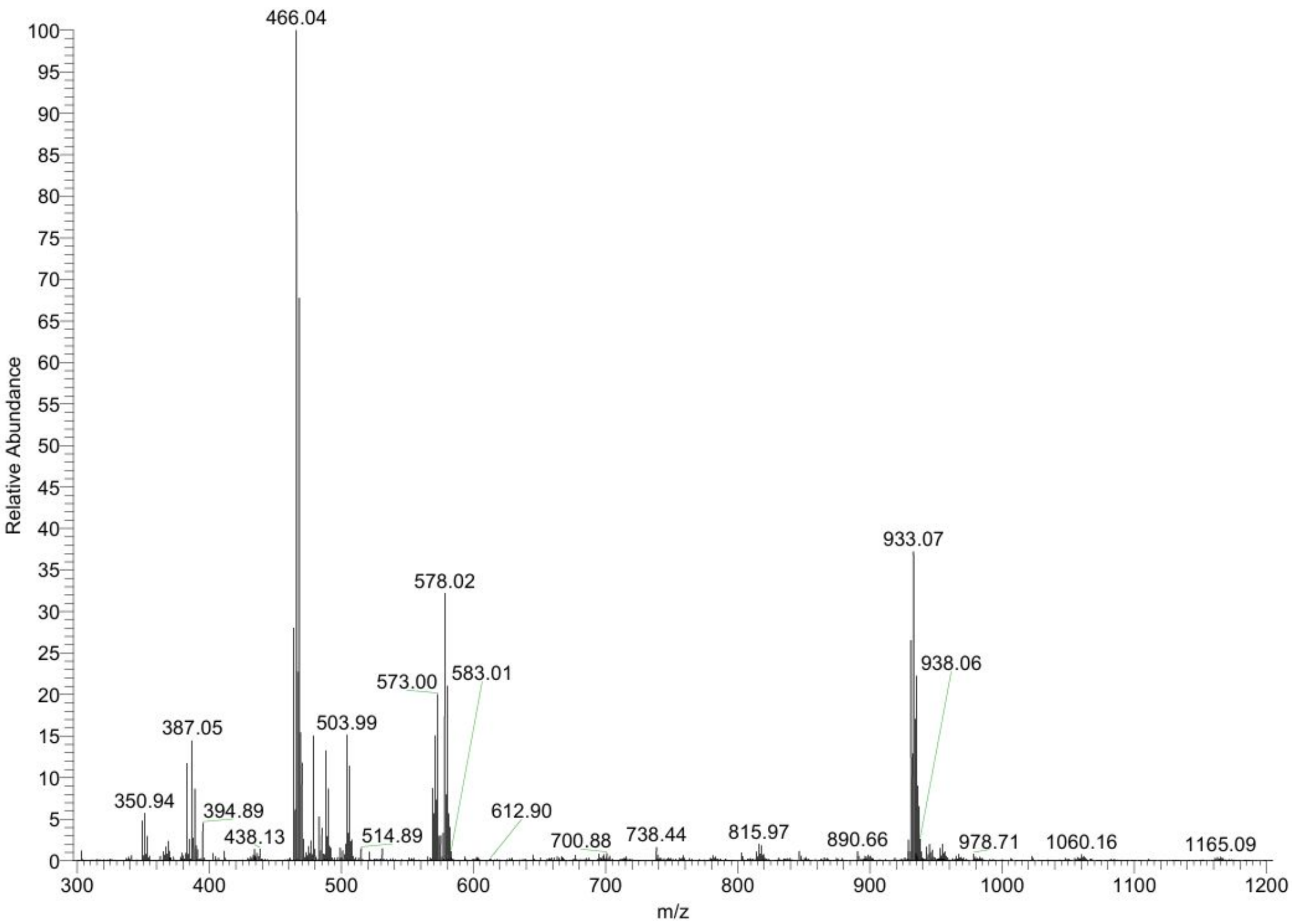

Figure S6. MS spectrum of **3a**

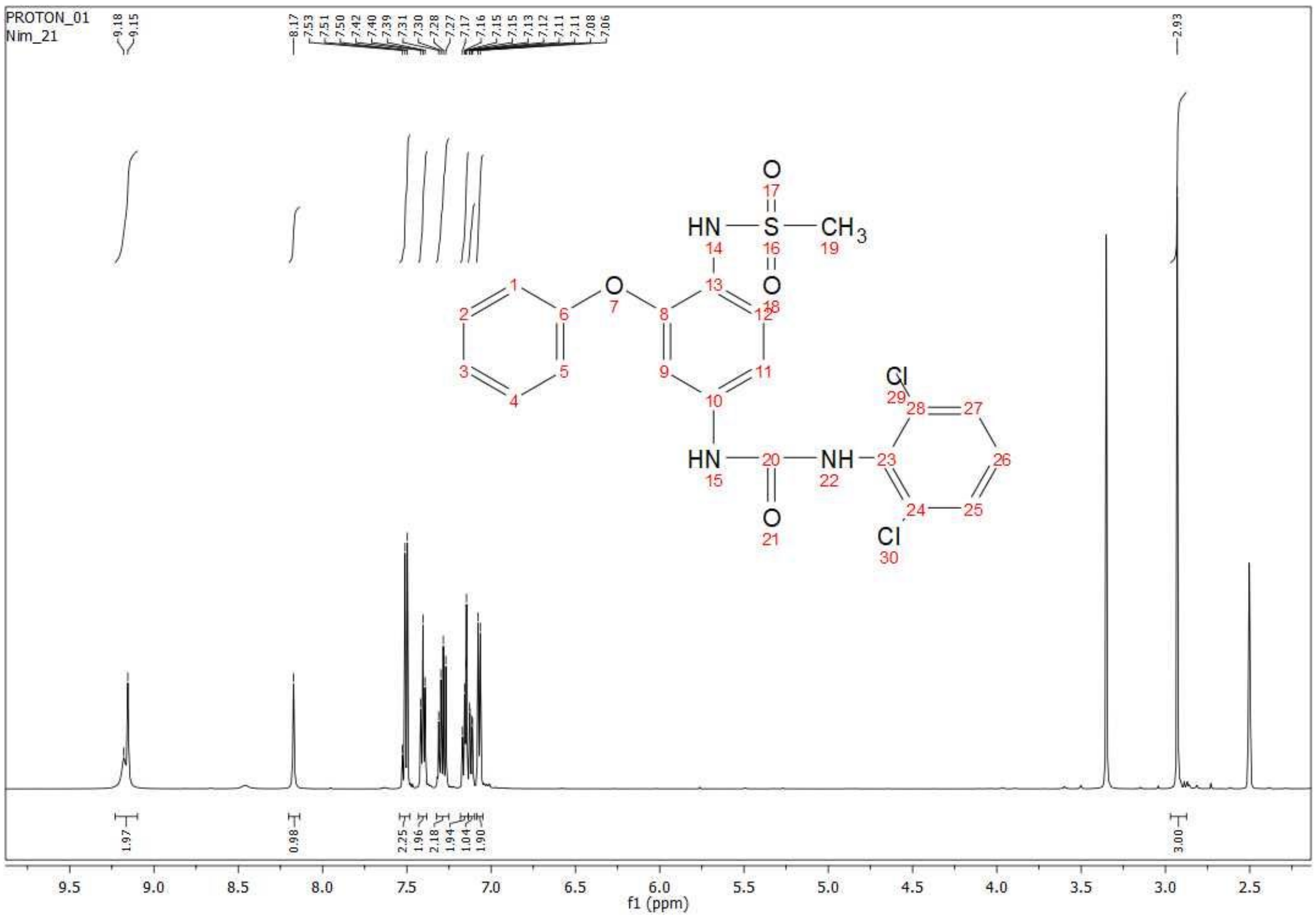

Figure S7. ^{1}H NMR spectrum (600 MHz, DMSO) of **3a**

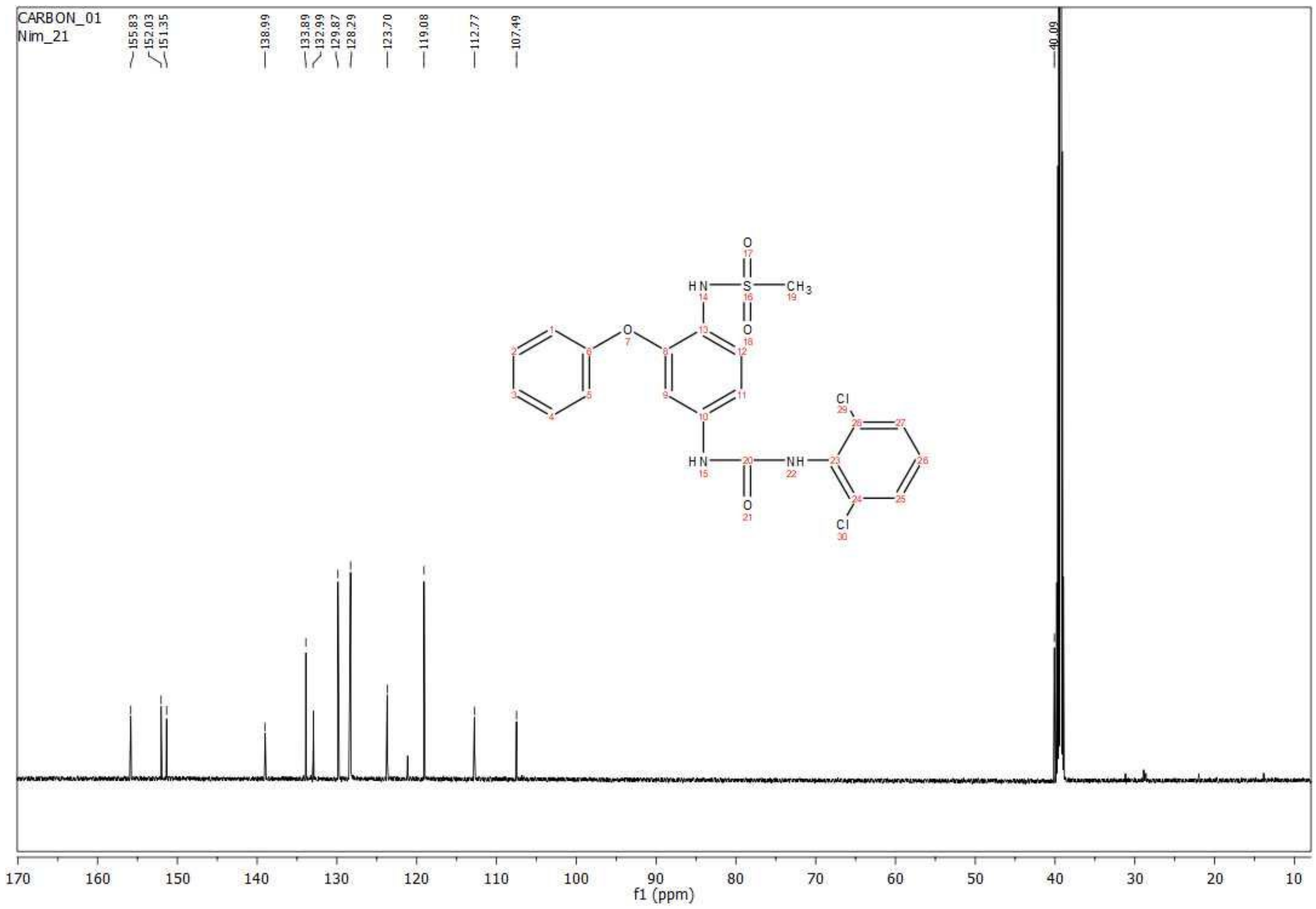

Figure S8. ^{13}C NMR spectrum (150 MHz, DMSO) of **3a.**

Copies of the IR, MS, ^{1}H NMR and ^{13}C NMR spectra of synthesized N-{4-[3-(3-Chloro-phenyl)-ureido]-2-phenoxy-phenyl}-methanesulfonamide (3b)

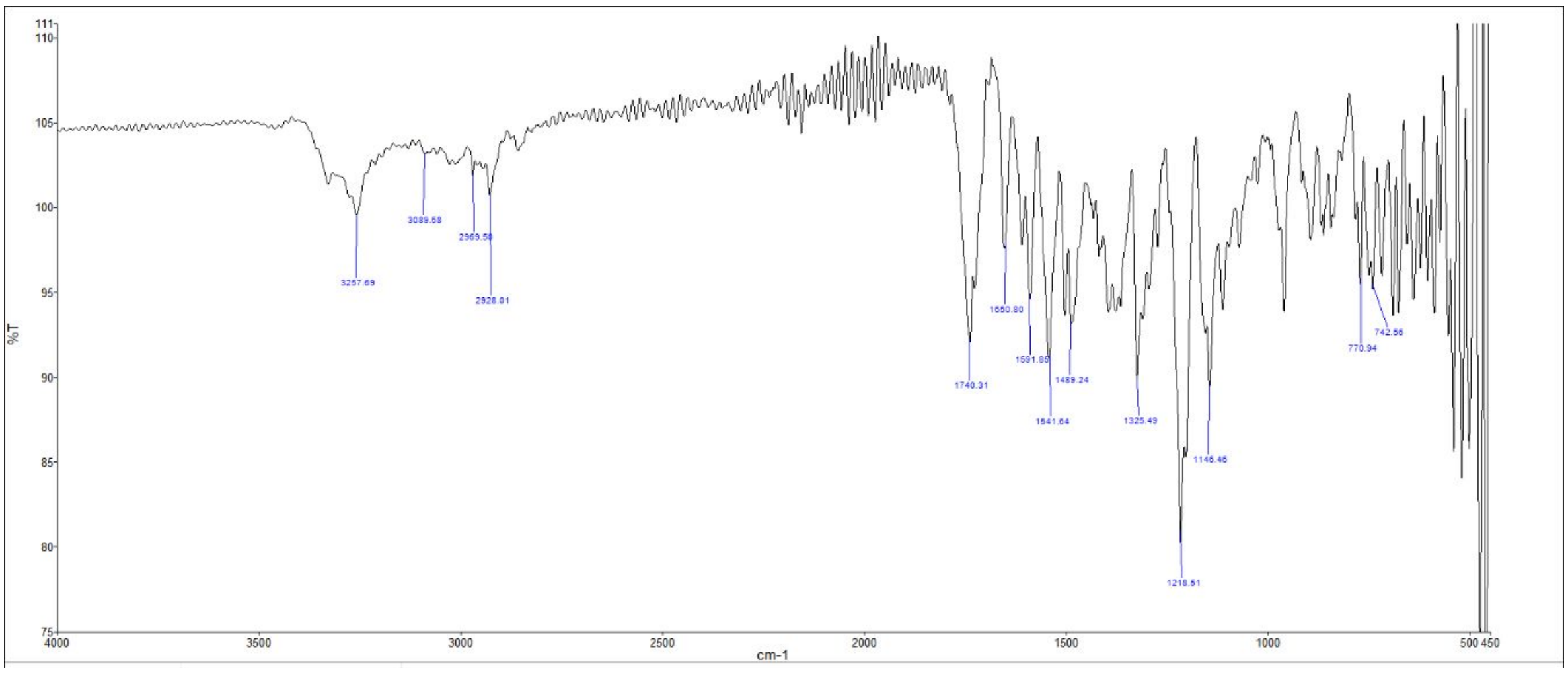

Figure S9. FTIR spectrum of **3b**

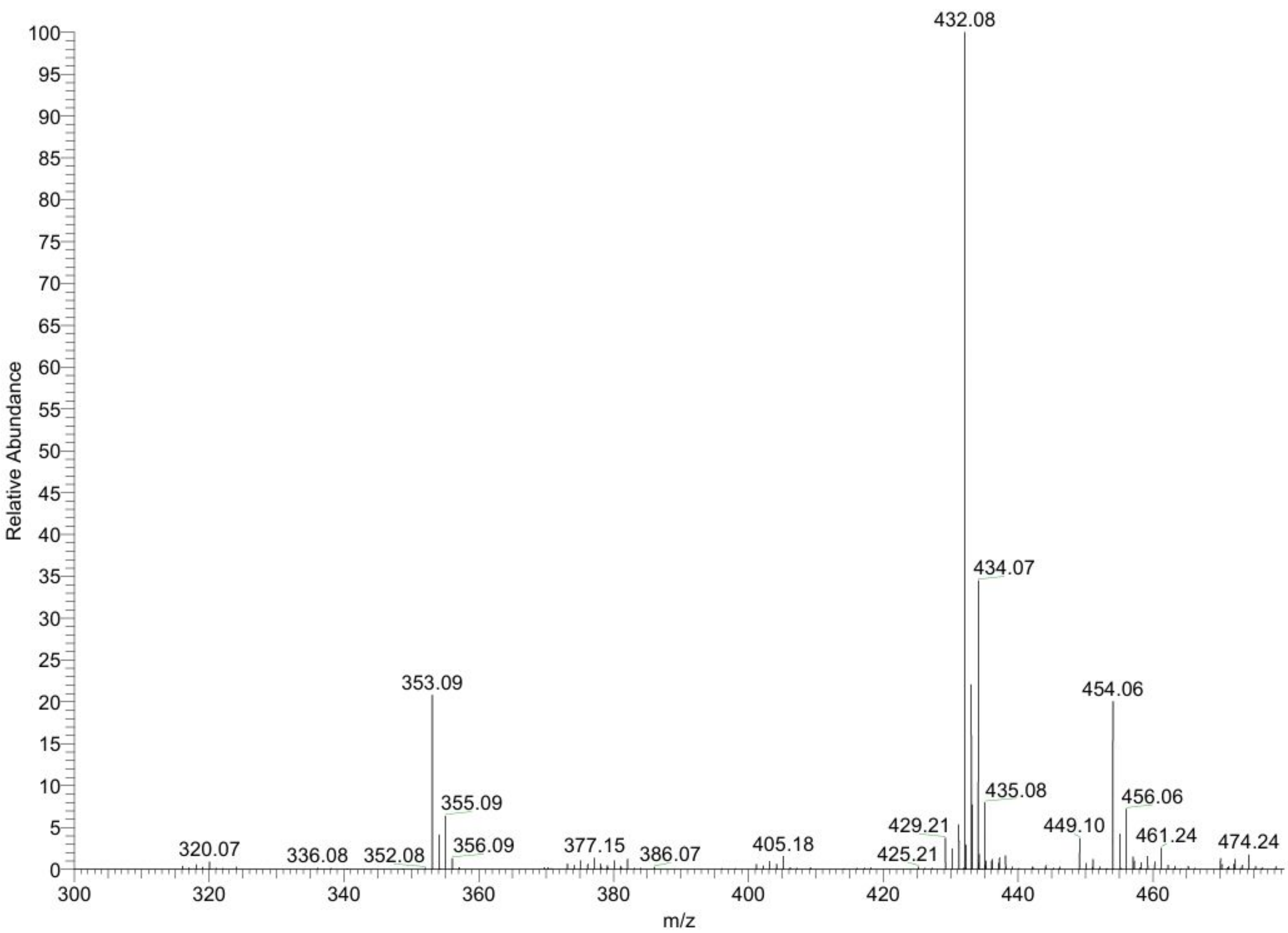

Figure S10. MS spectrum of **3b**

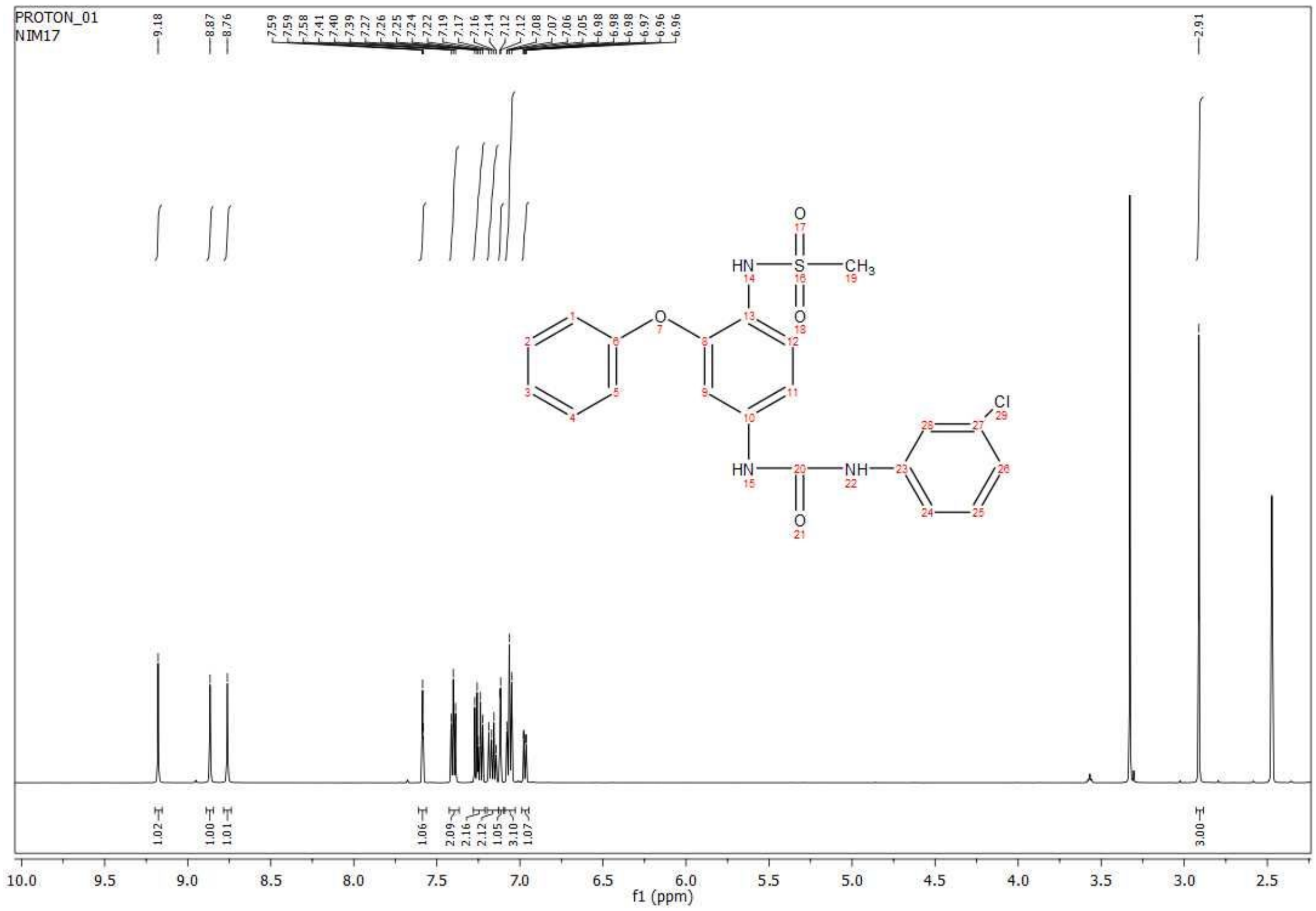

Figure S11. ^{1}H NMR spectrum (600 MHz, DMSO) of **3b.**

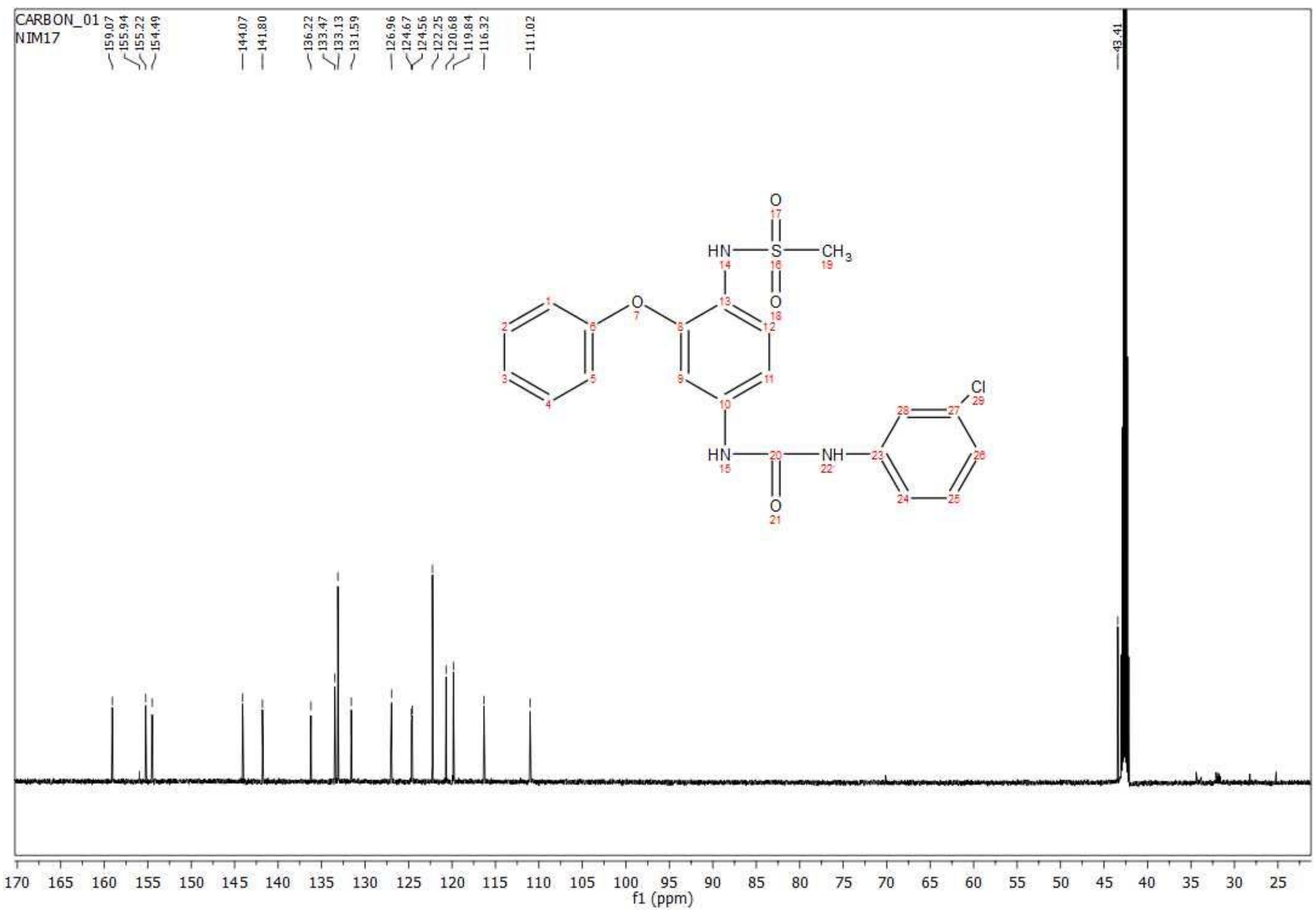

Figure S12. ^{13}C NMR spectrum (150 MHz, DMSO) of **3b.**

Copies of the FTIR, MS, ^{1}H NMR and ^{13}C NMR spectra of synthesized N-{4-[3-(2,4-Dimethoxy-phenyl)-ureido]-2-phenoxy-phenyl}-methanesulfonamide (3c)

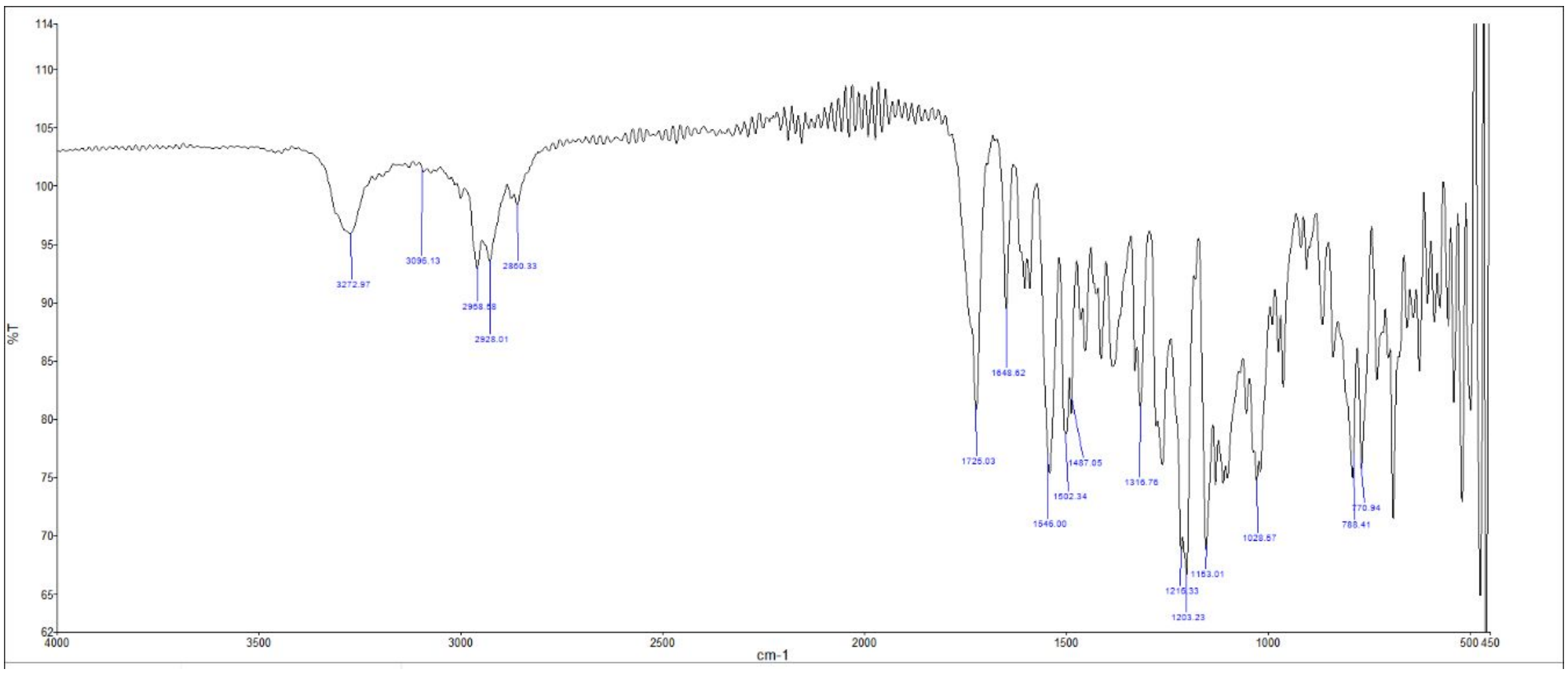

Figure S13. FTIR spectrum of **3c**

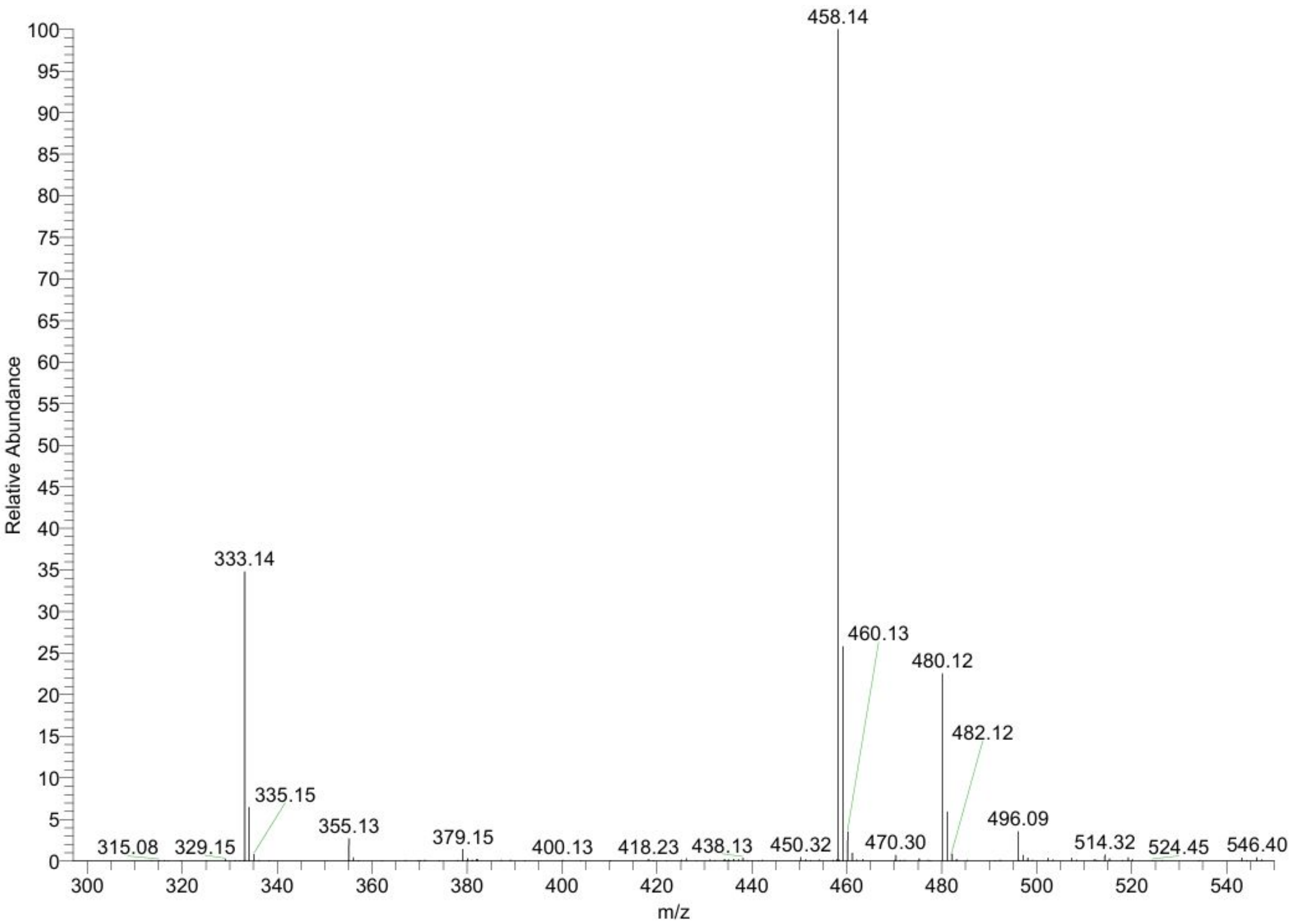

Figure S14. MS spectrum of **3c**

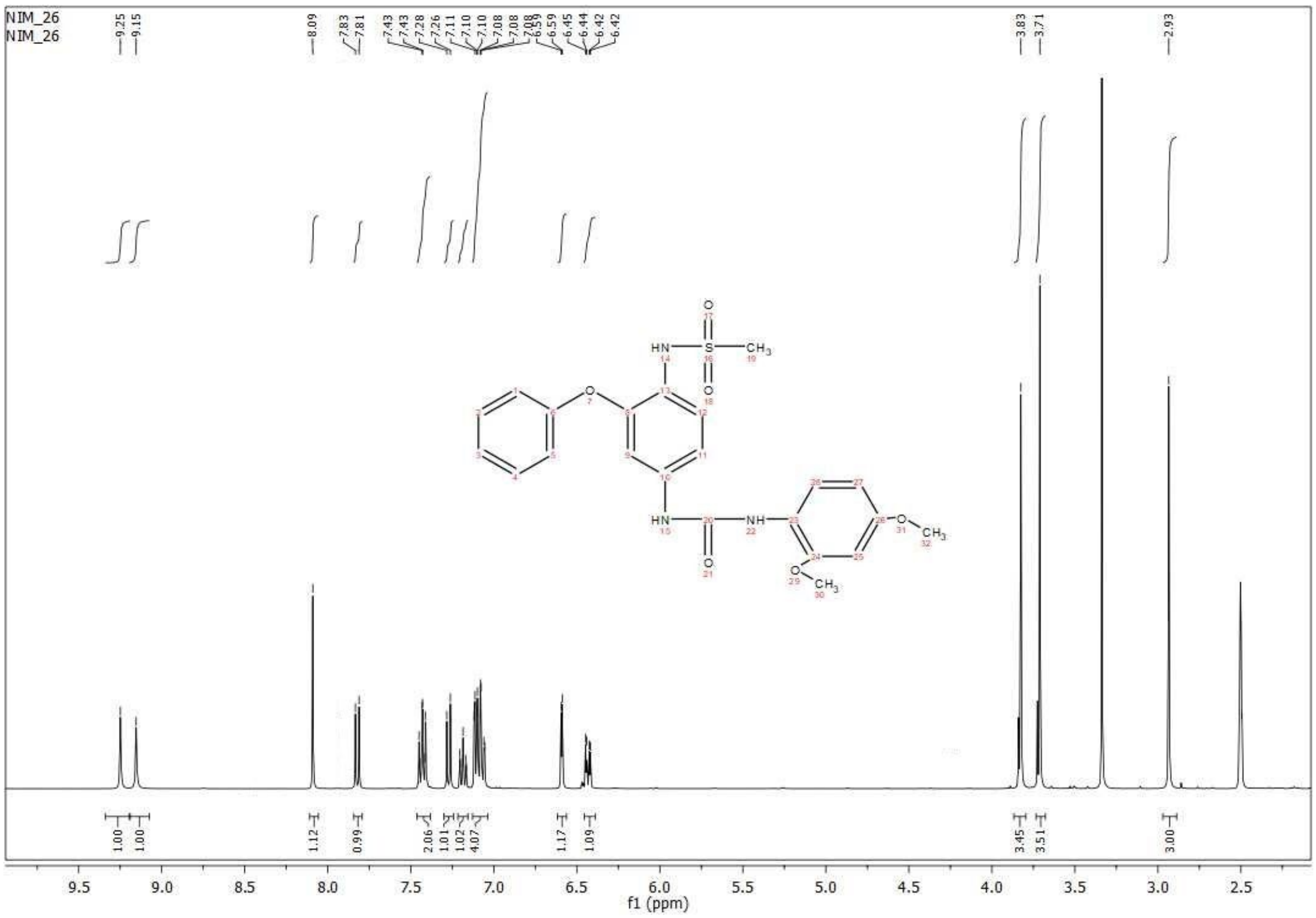

Figure S15. ^{1}H NMR spectrum (600 MHz, DMSO) of **3c.**

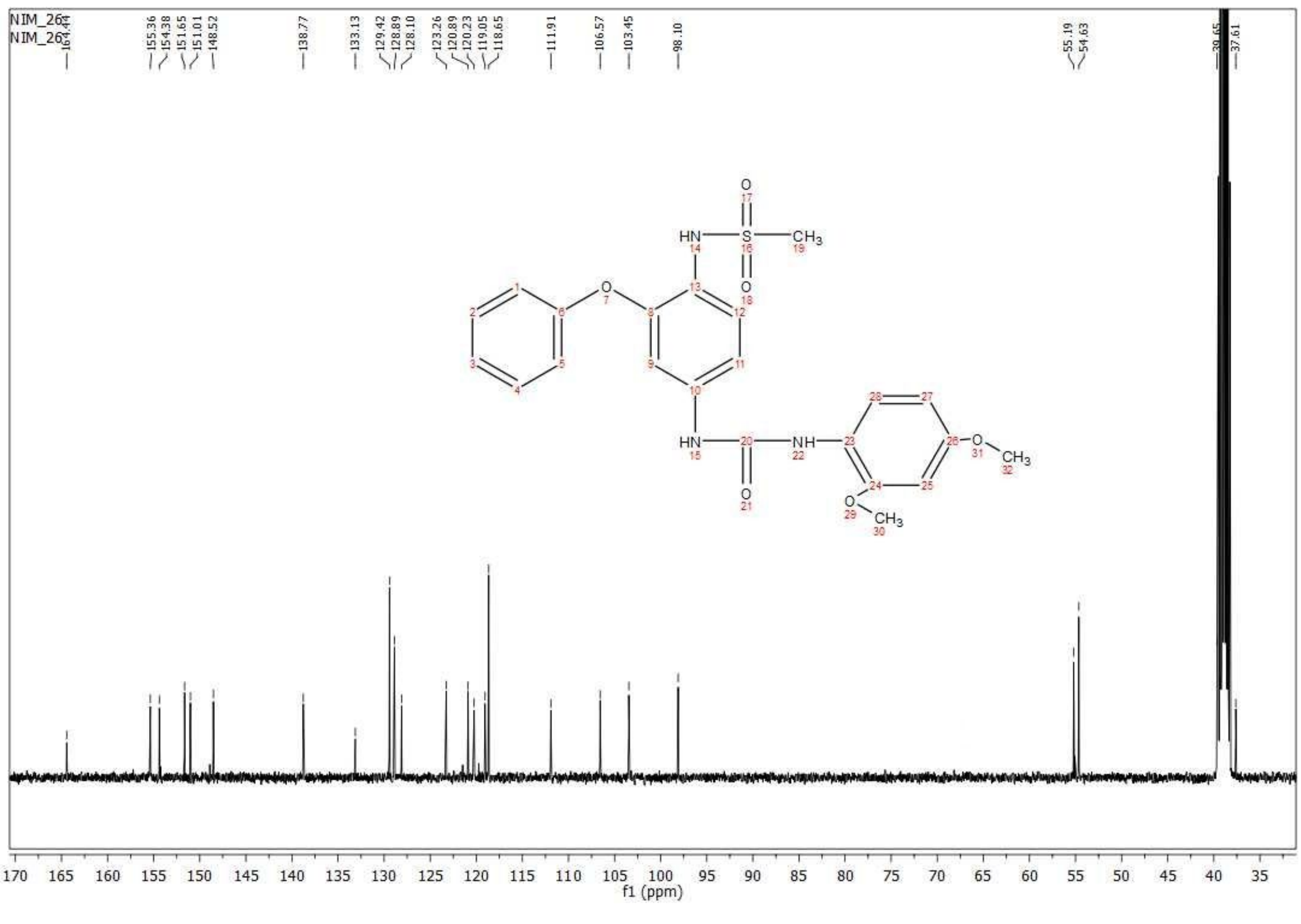

Figure S16. ^{13}C NMR spectrum (150 MHz, DMSO) of **3c.**

Copies of the FTIR, MS, ^{1}H NMR and ^{13}C NMR spectra of synthesized N-{4-[3-(4-Bromo-phenyl)-ureido]-2-phenoxy-phenyl}-methanesulfonamide (3d)

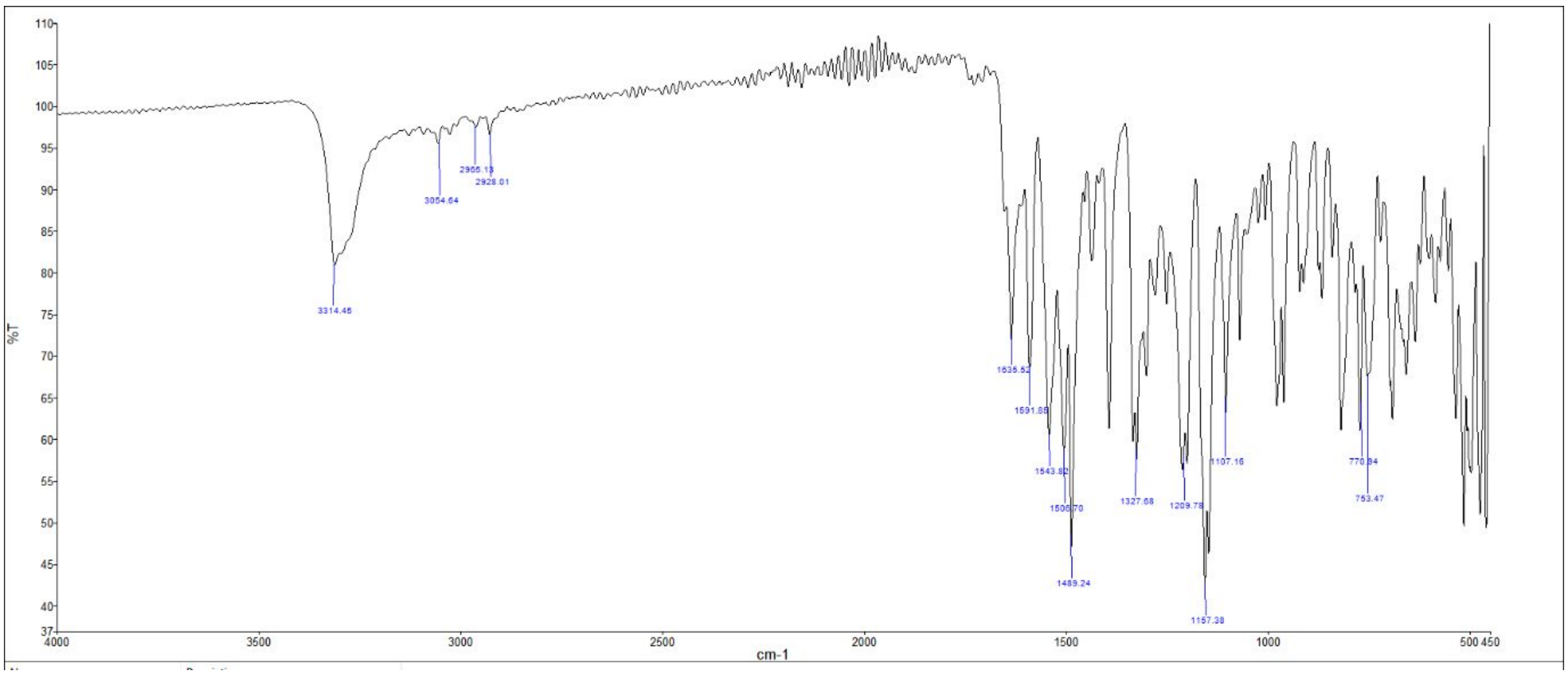

Figure S17. FTIR spectrum of **3d**

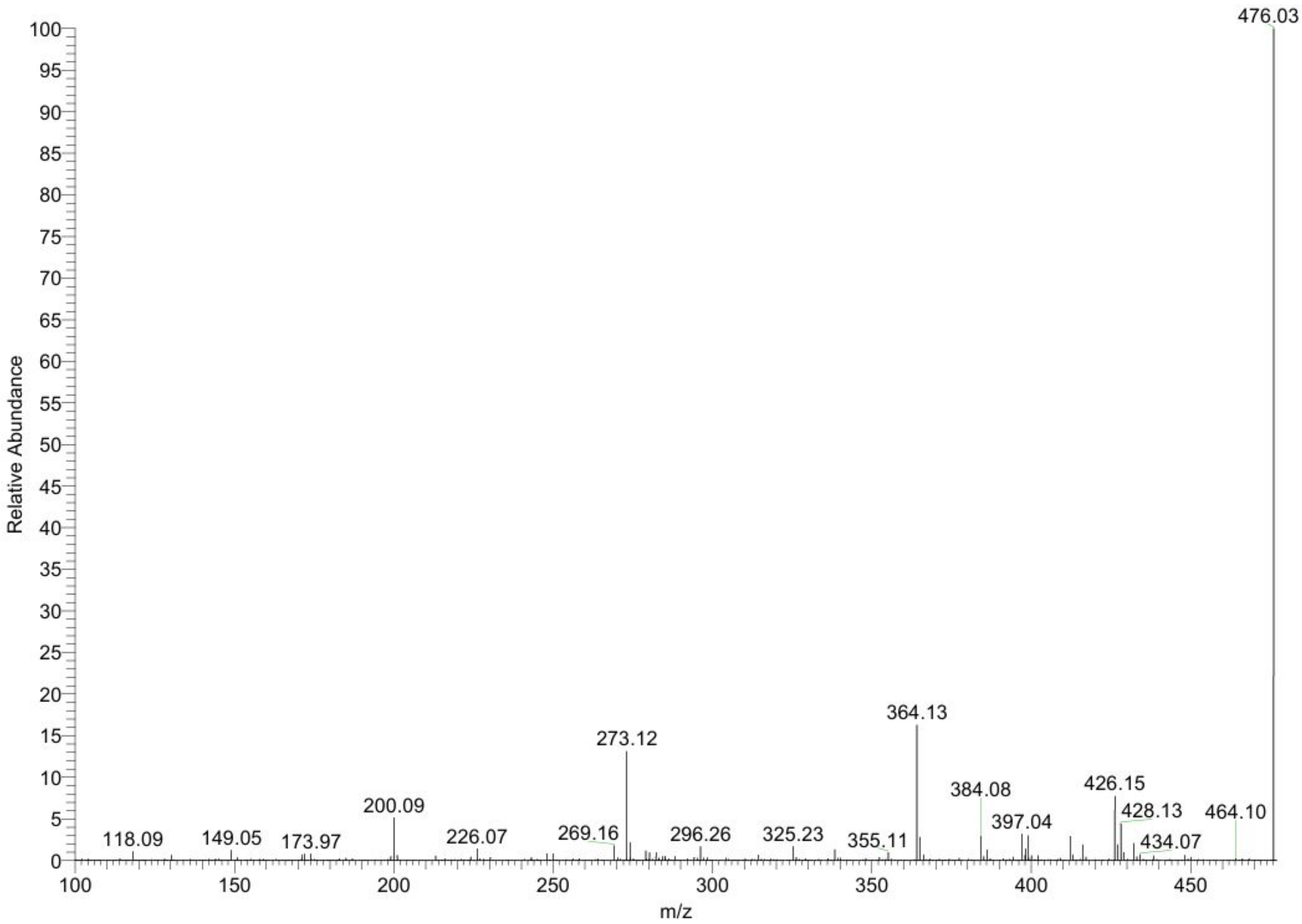

Figure S18. MS spectrum of **3d**

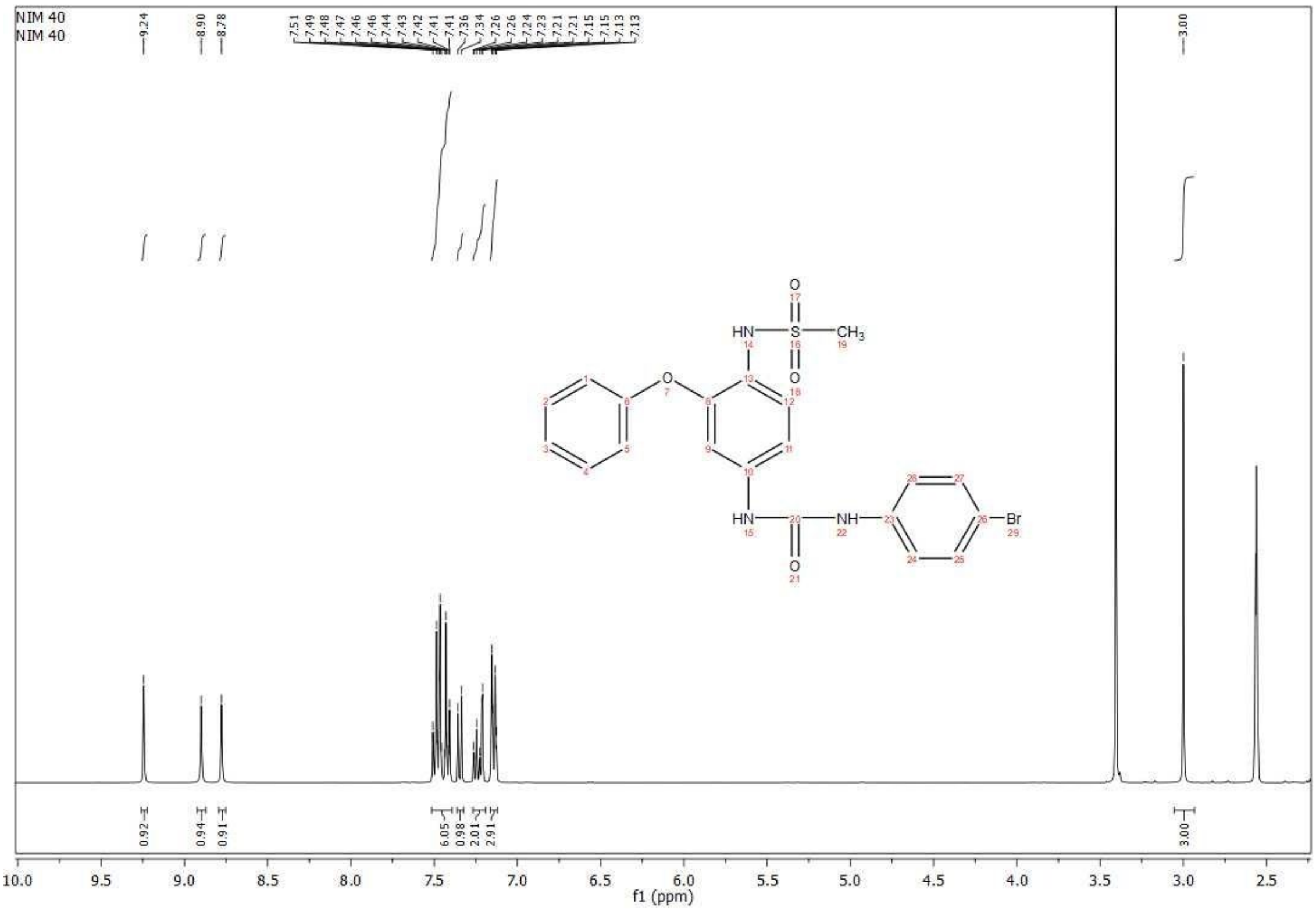

Figure S19. ^{1}H NMR spectrum (600 MHz, DMSO) of **3d**

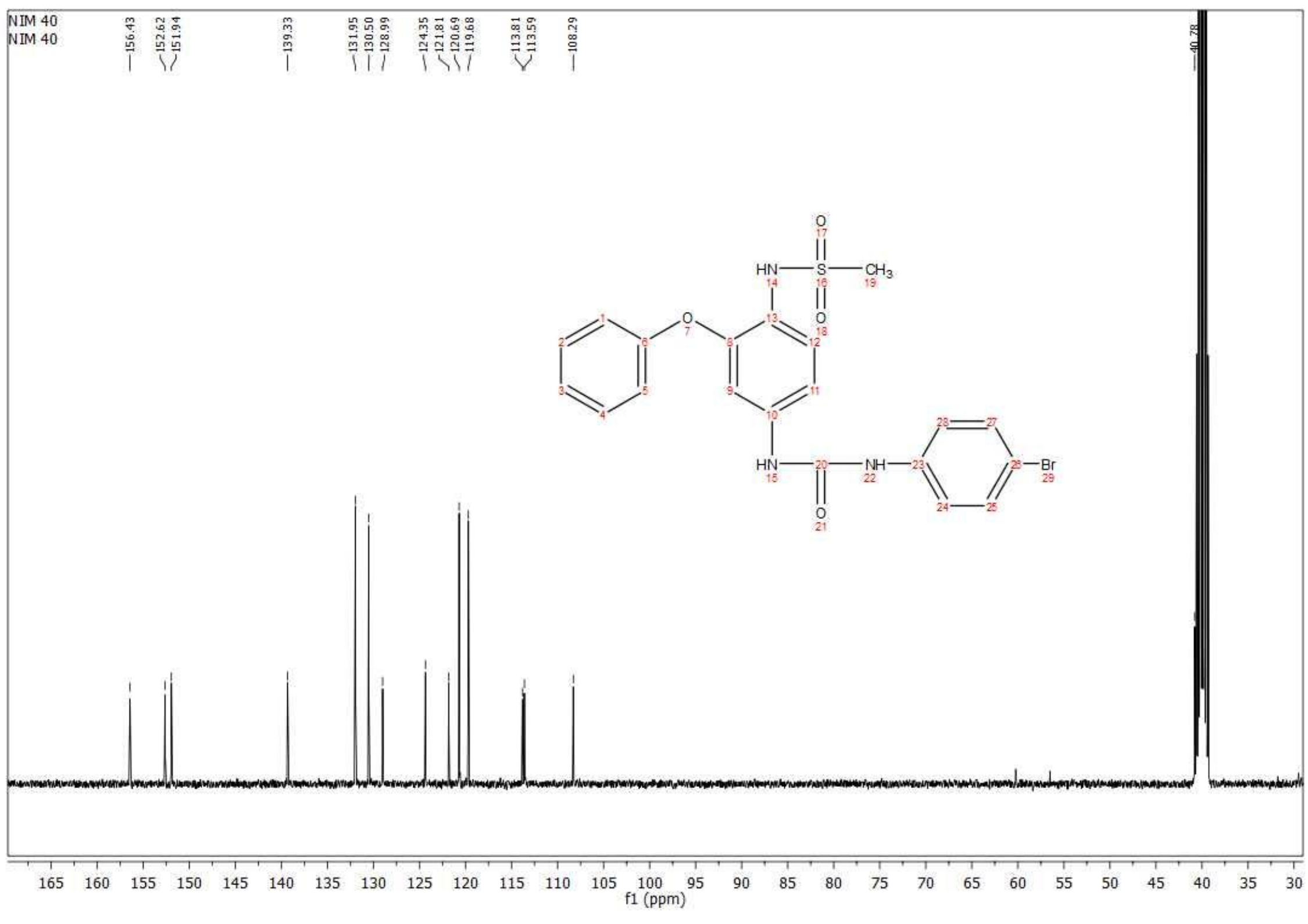

Figure S20. ^{13}C NMR spectrum (150 MHz, DMSO) of **3d.**

Copies of the FTIR, MS, ^{1}H NMR and ^{13}C NMR spectra of synthesized N-{2-Phenoxy-4-[3-(3-trifluoromethyl-phenyl)-ureido]-phenyl}-methanesulfonamide (3e)

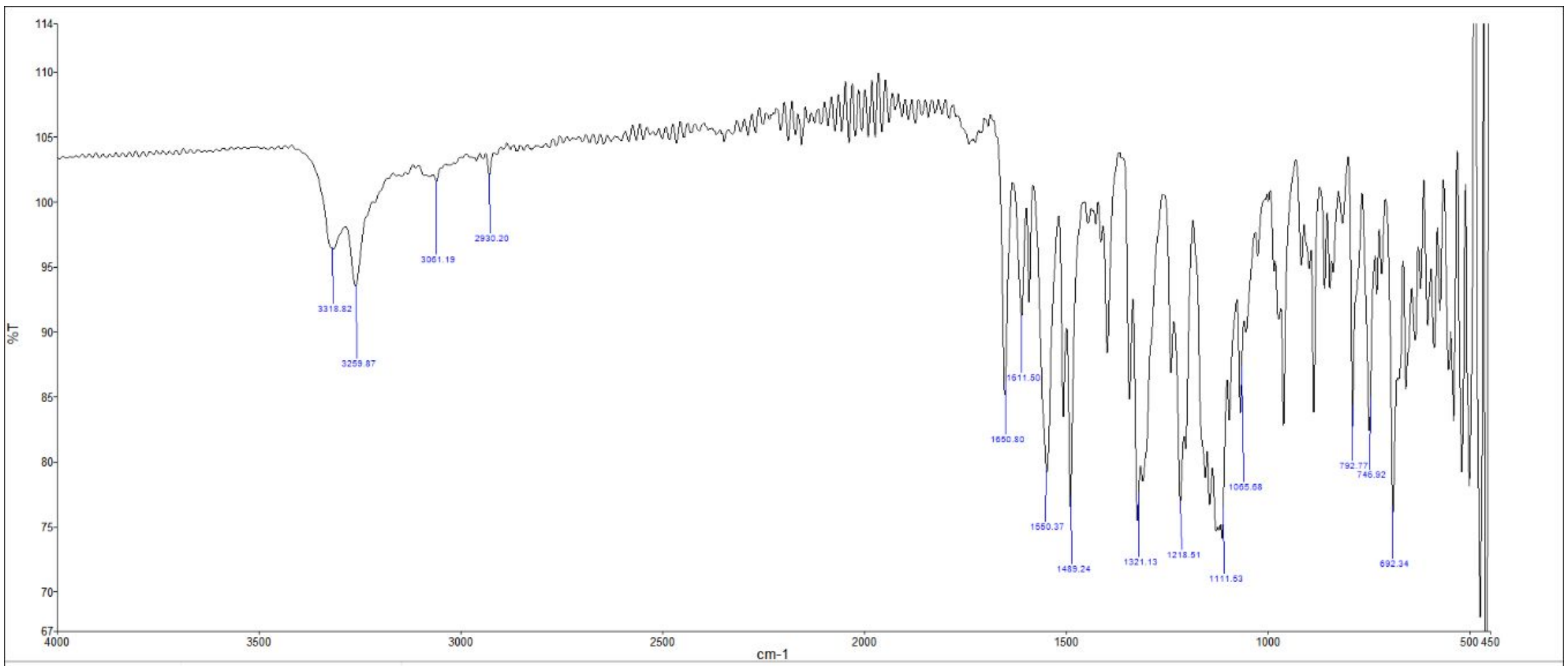

Figure S21. FTIR spectrum of **3e**

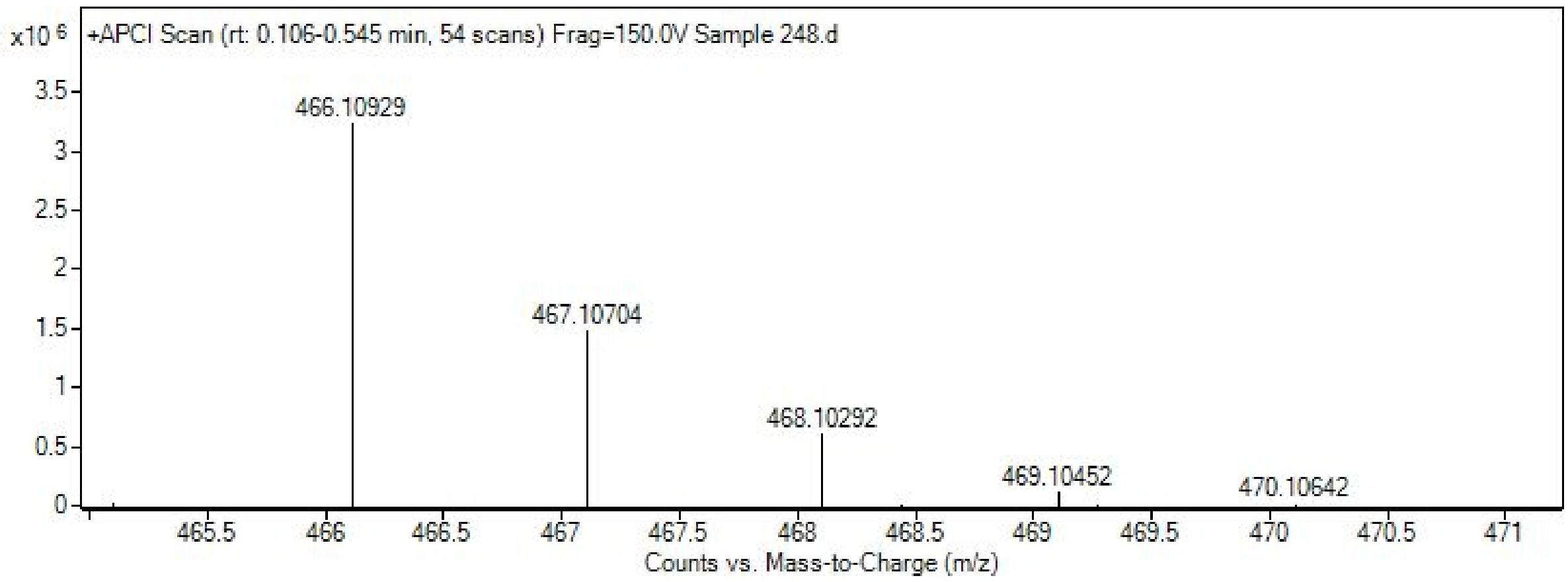

Figure S22. MS spectrum of **3e**

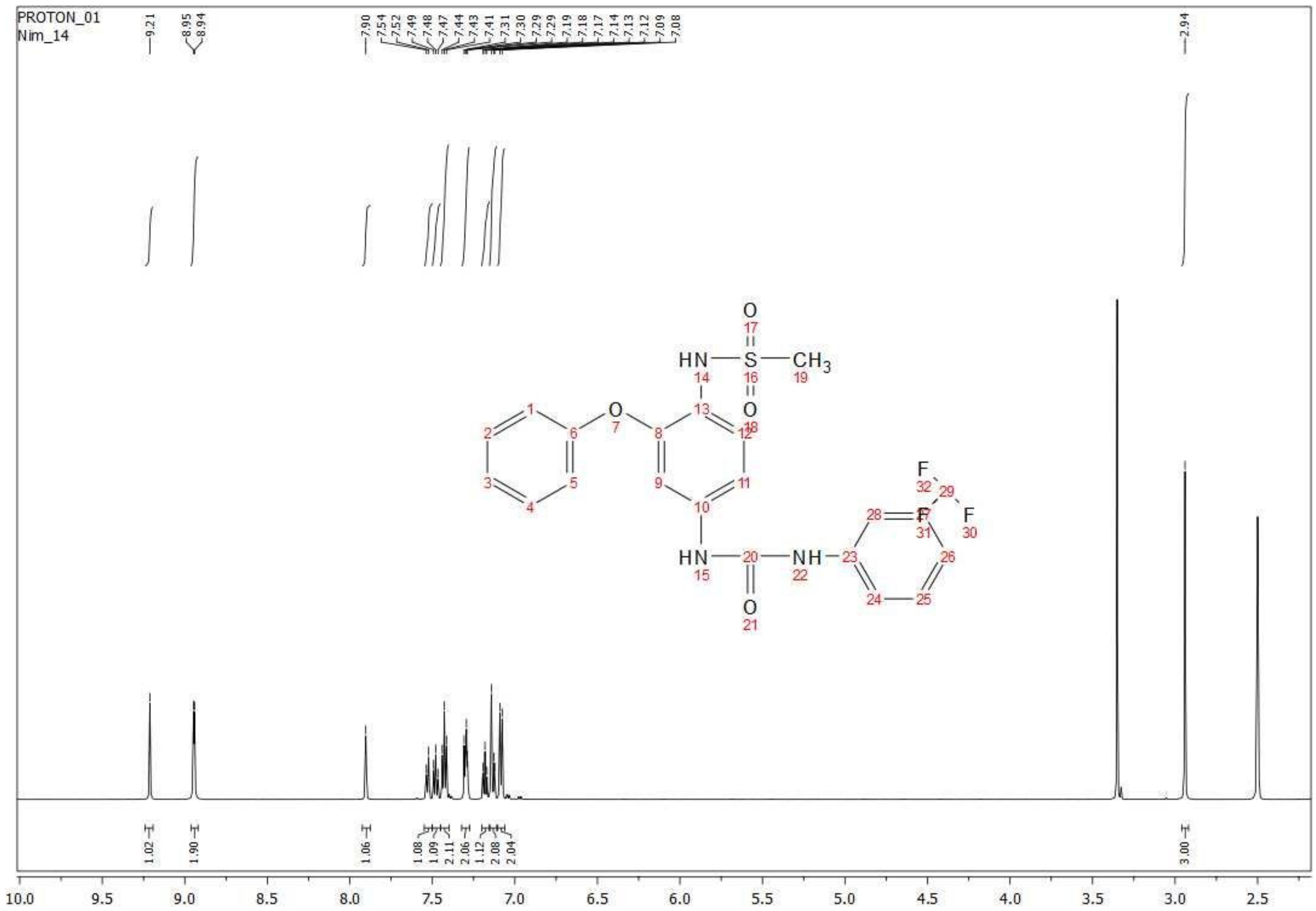

Figure S23. ^{1}H NMR spectrum (600 MHz, DMSO) of **3e**

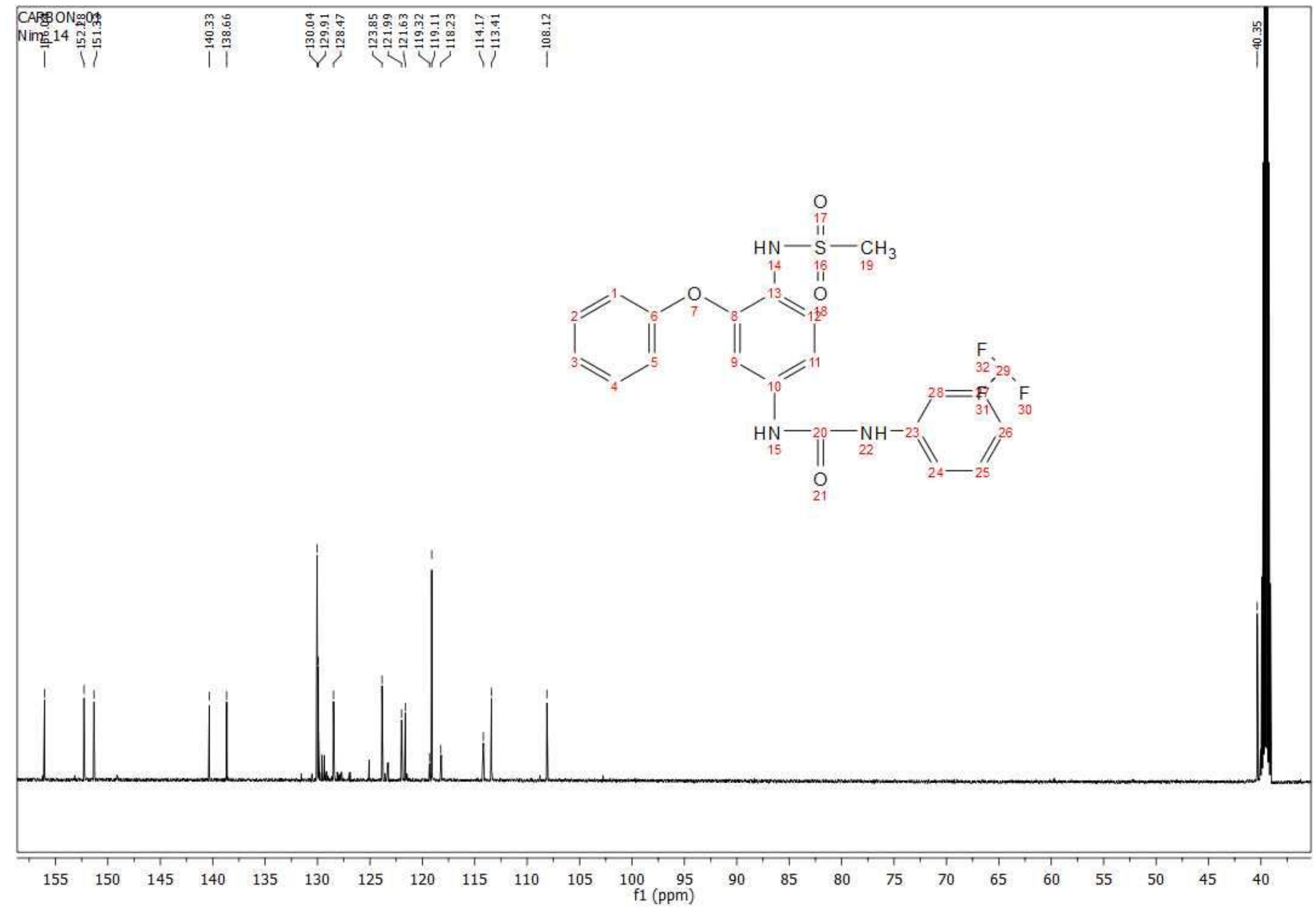

Figure S24. ^{13}C NMR spectrum (150 MHz, DMSO) of **3e.**

Copies of the FTIR, MS, ^{1}H NMR and ^{13}C NMR spectra of synthesized N-{4-[3-(4-Fluoro-benzyl)-ureido]-2-phenoxy-phenyl}-methanesulfonamide (3f)

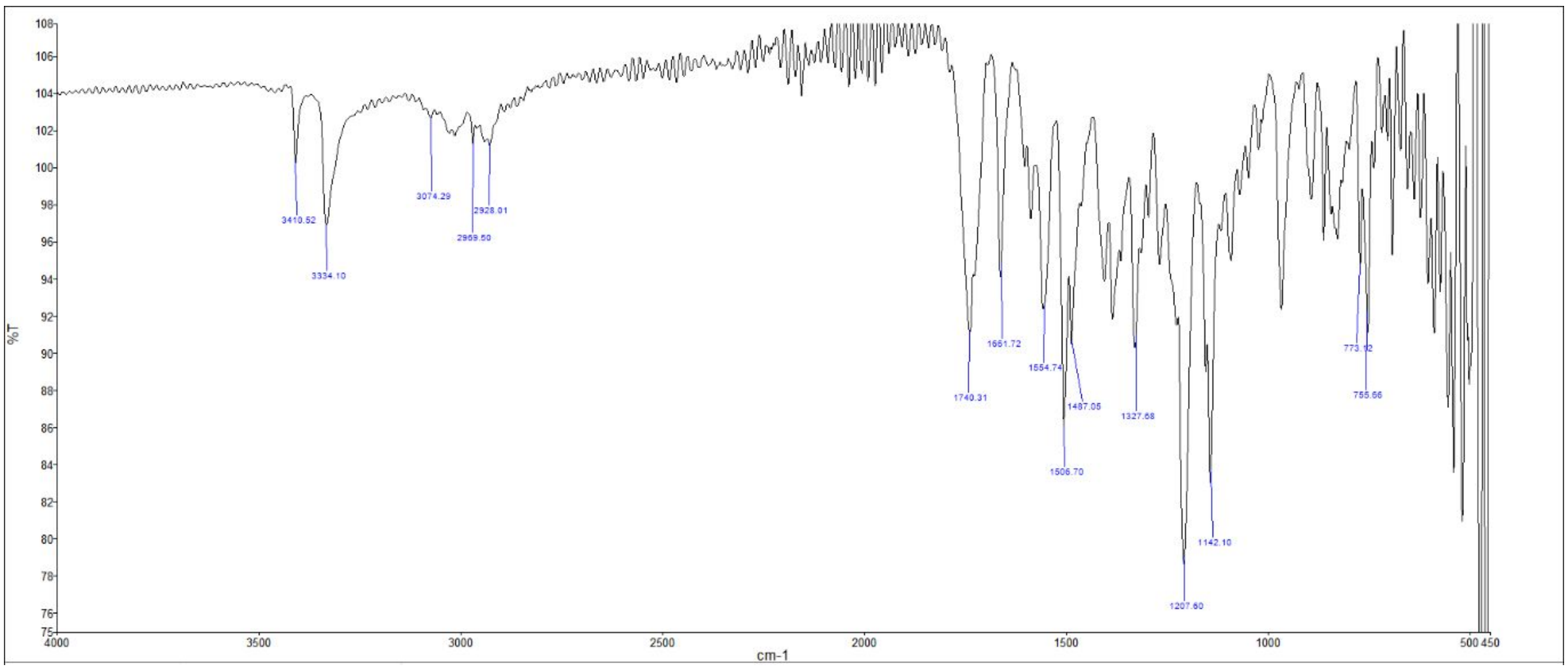

Figure S25. FTIR spectrum of **3f**

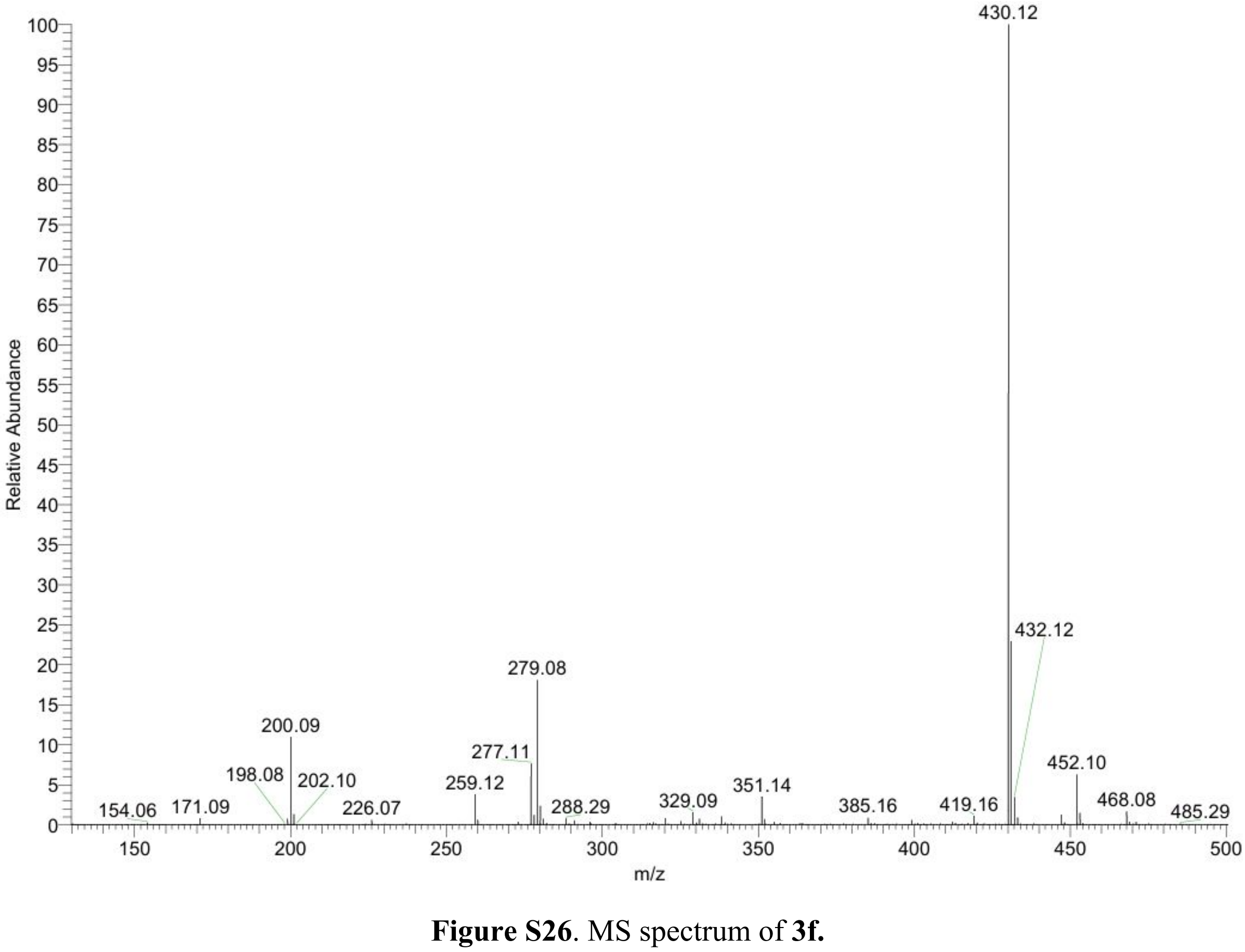

Figure S26. MS spectrum of **3f.**

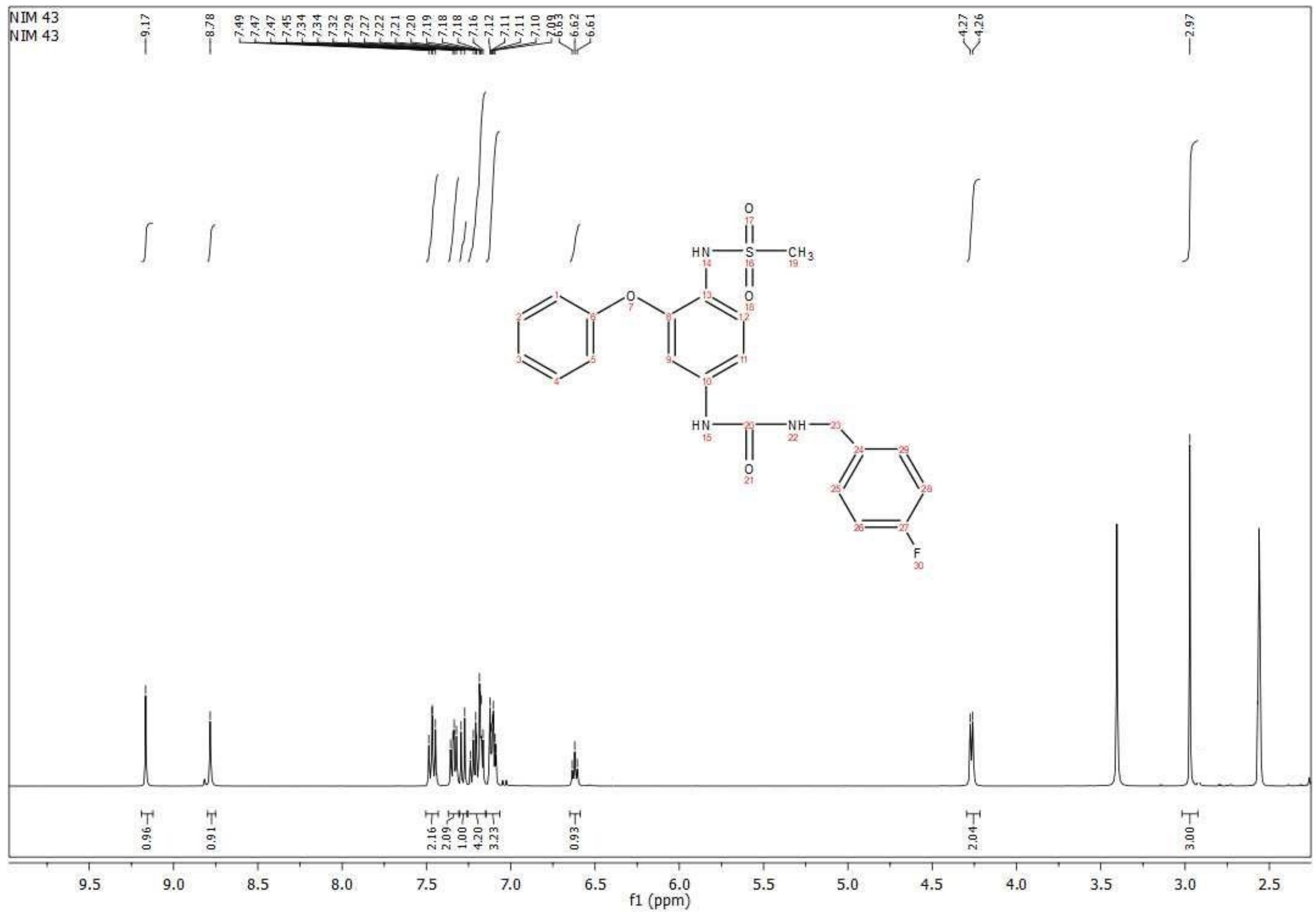

Figure S27. ^{1}H NMR spectrum (600 MHz, DMSO) of **3f.**

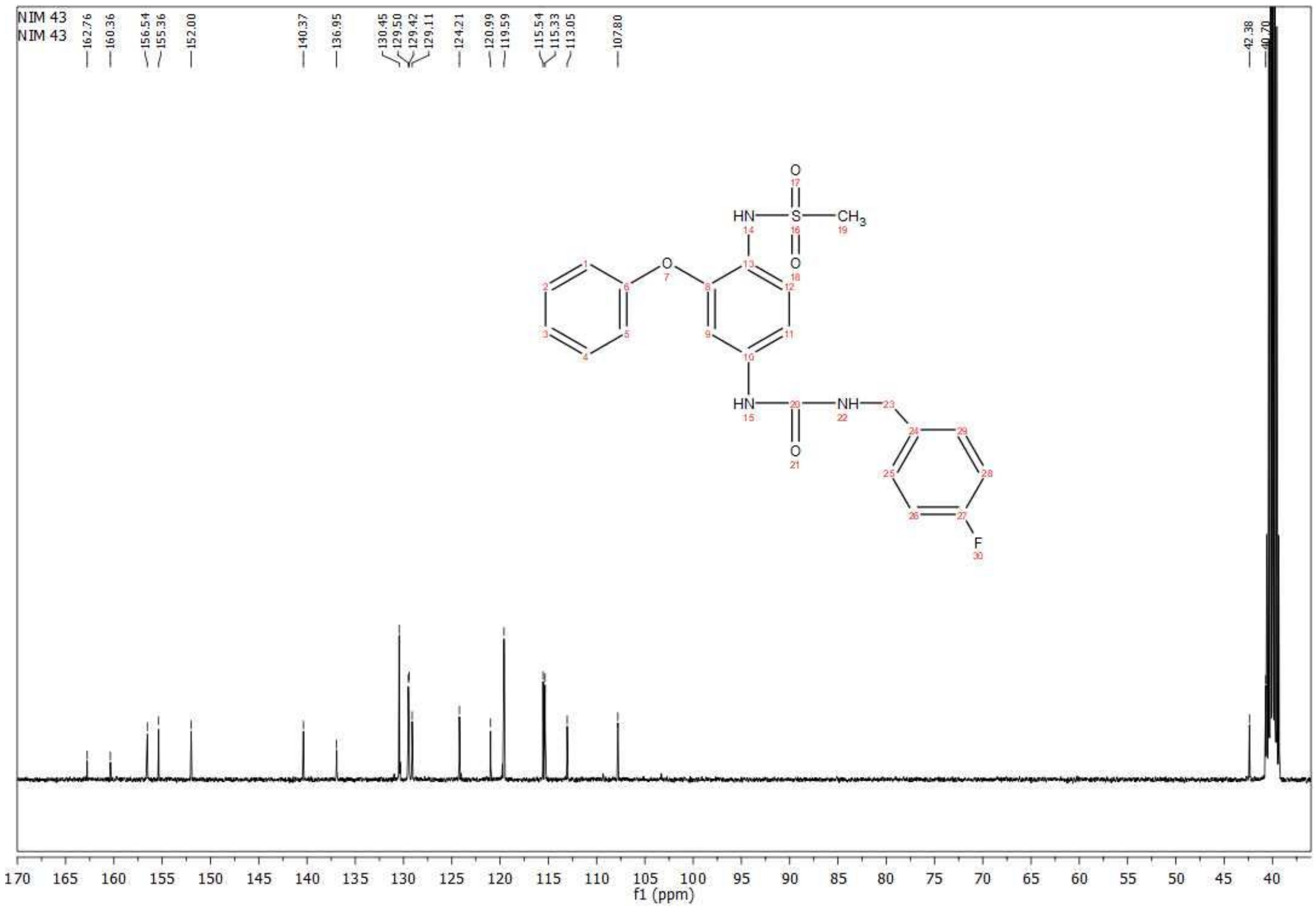

Figure S28. ^{13}C NMR spectrum (150 MHz, DMSO) of **3f.**

Copies of the FTIR, MS, ^{1}H NMR and ^{13}C NMR spectra of synthesized N-{4-[3-(3,5-Dichloro-phenyl)-ureido]-2-phenoxy-phenyl}-methanesulfonamide (3g)

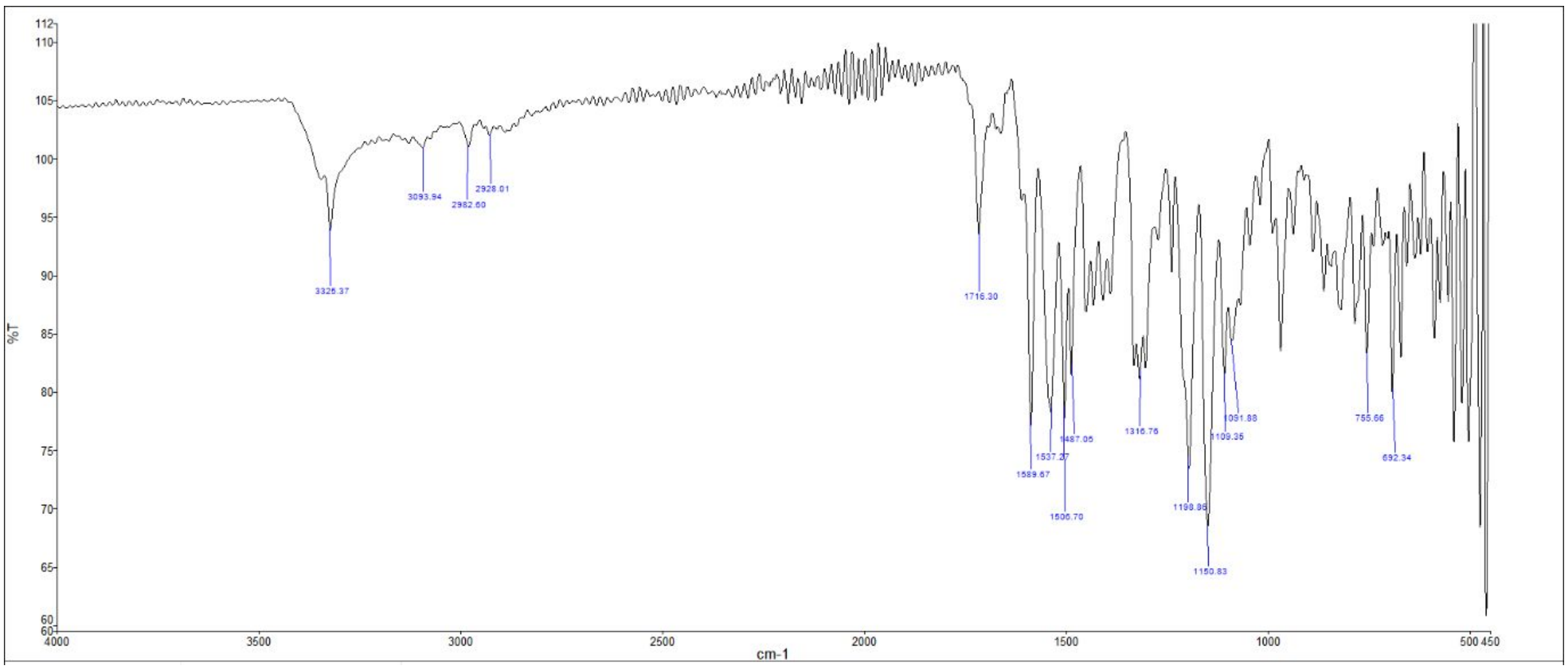

Figure S29. FTIR spectrum of **3g**

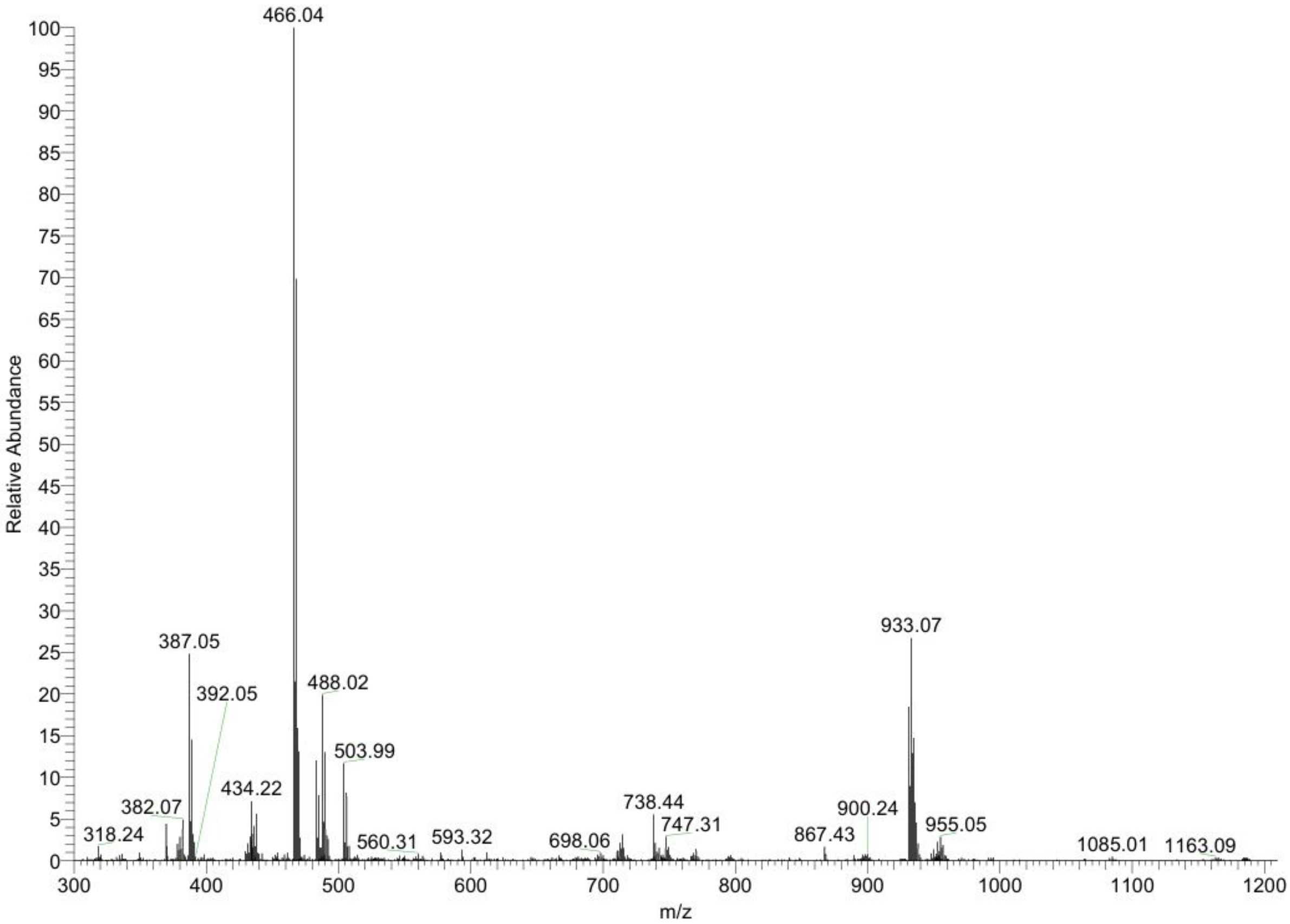

Figure S30. MS spectrum of **3g**

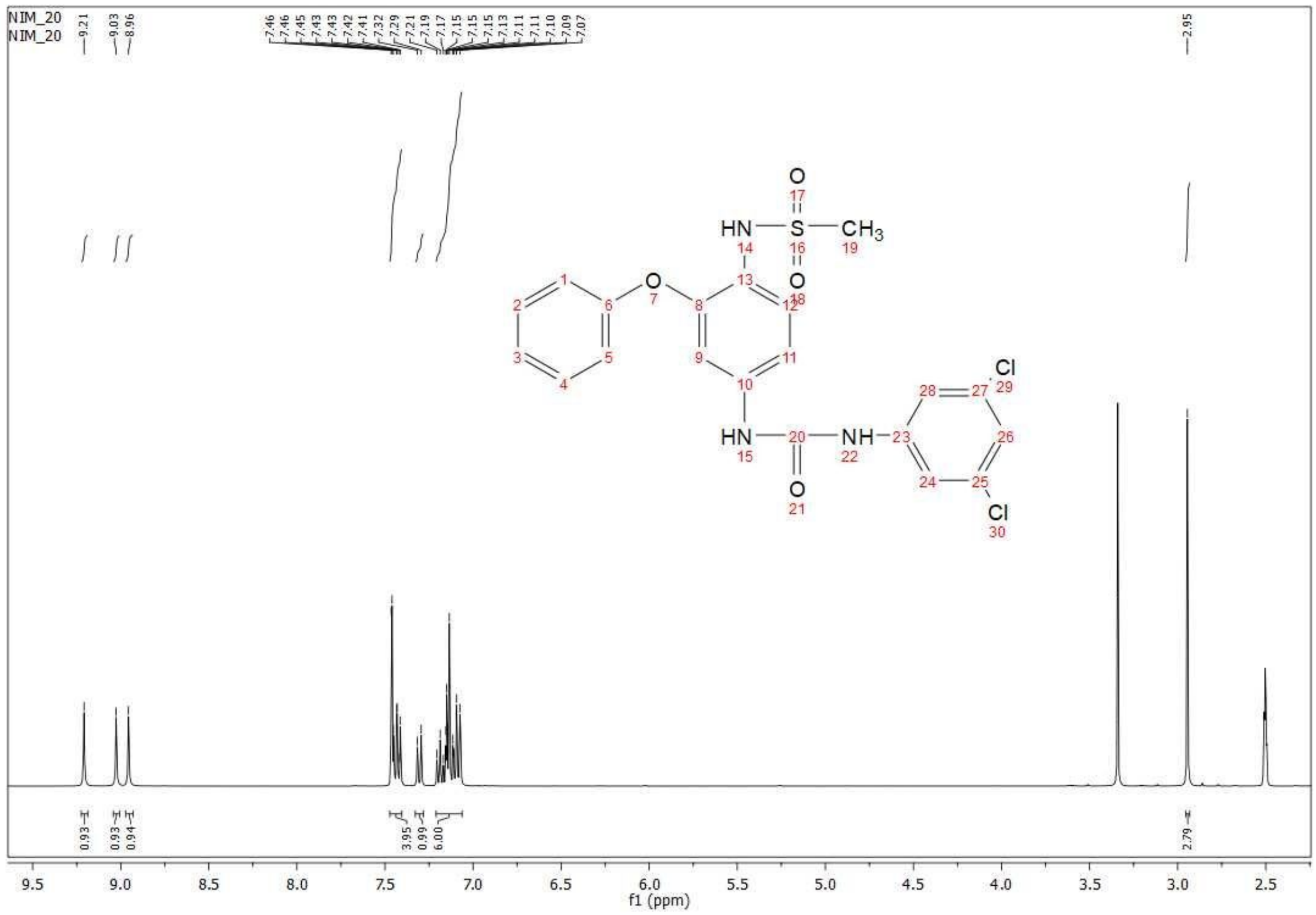

Figure S31. ^{1}H NMR spectrum (600 MHz, DMSO) of **3g.**

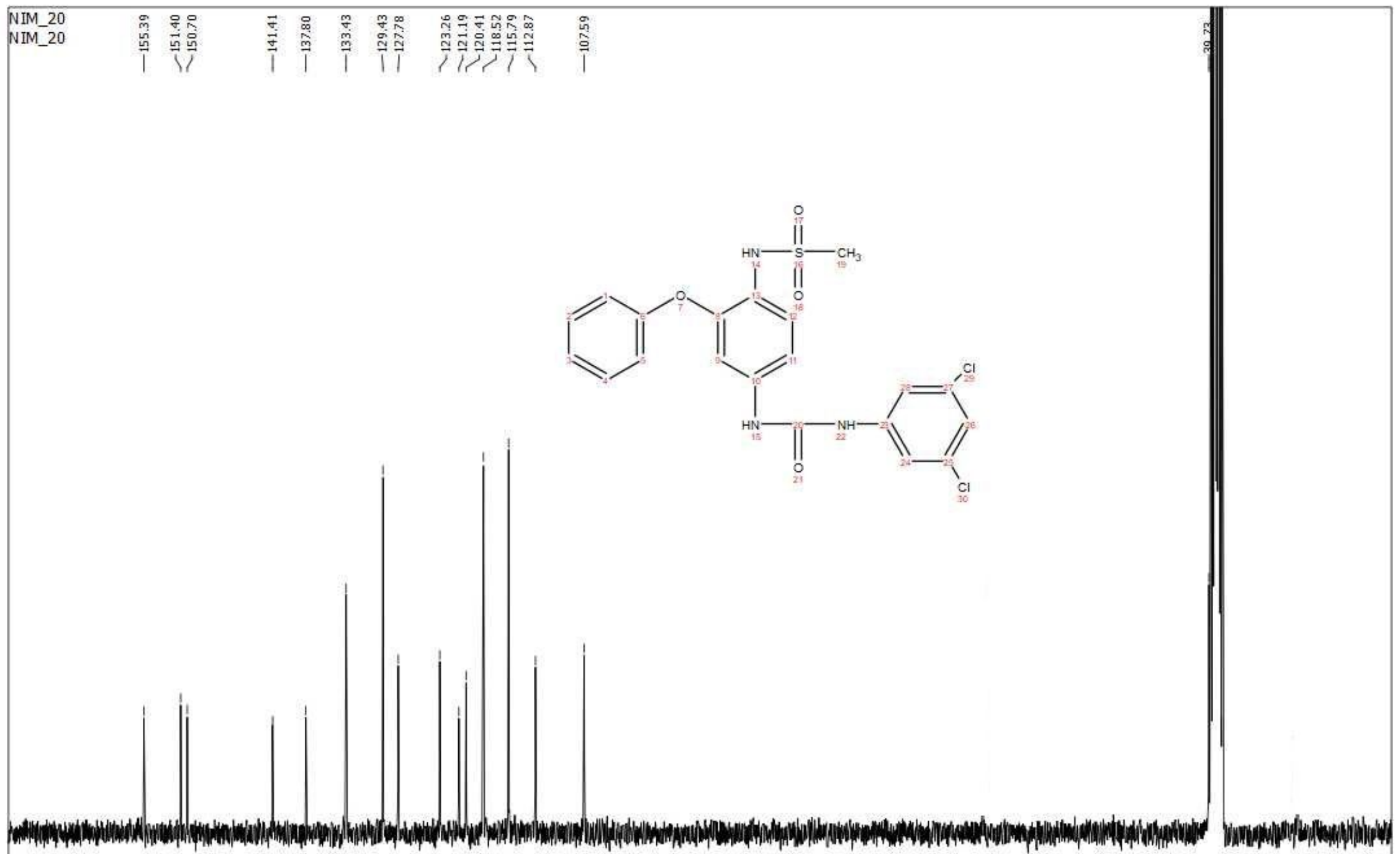

Figure S32. ^{13}C NMR spectrum (150 MHz, DMSO) of **3g.**

Copies of the FTIR, MS, ^{1}H NMR and ^{13}C NMR spectra of synthesized N-{4-[3-(3-Chloro-4-methyl-phenyl)-ureido]-2-phenoxy-phenyl}-methanesulfonamide (3h)

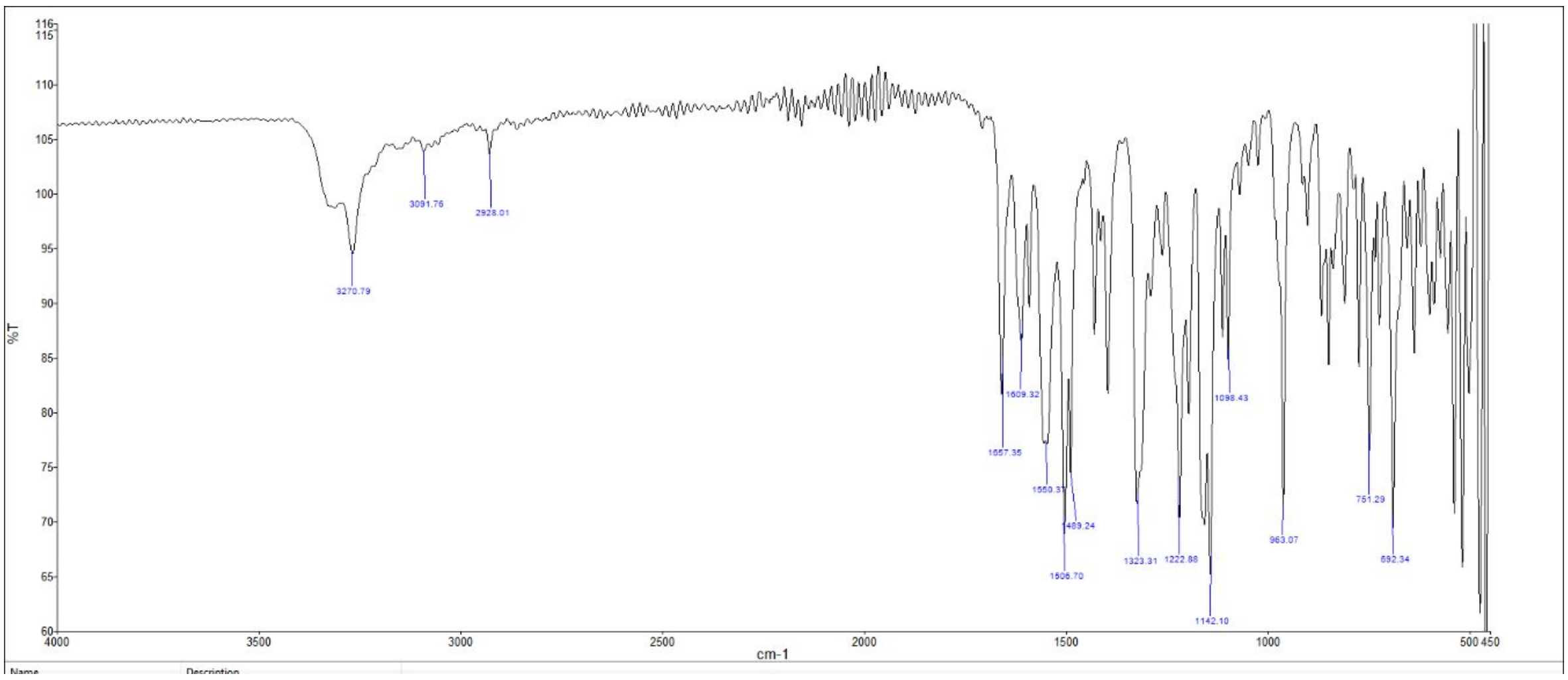

Figure S33. FTIR spectrum of **3h**

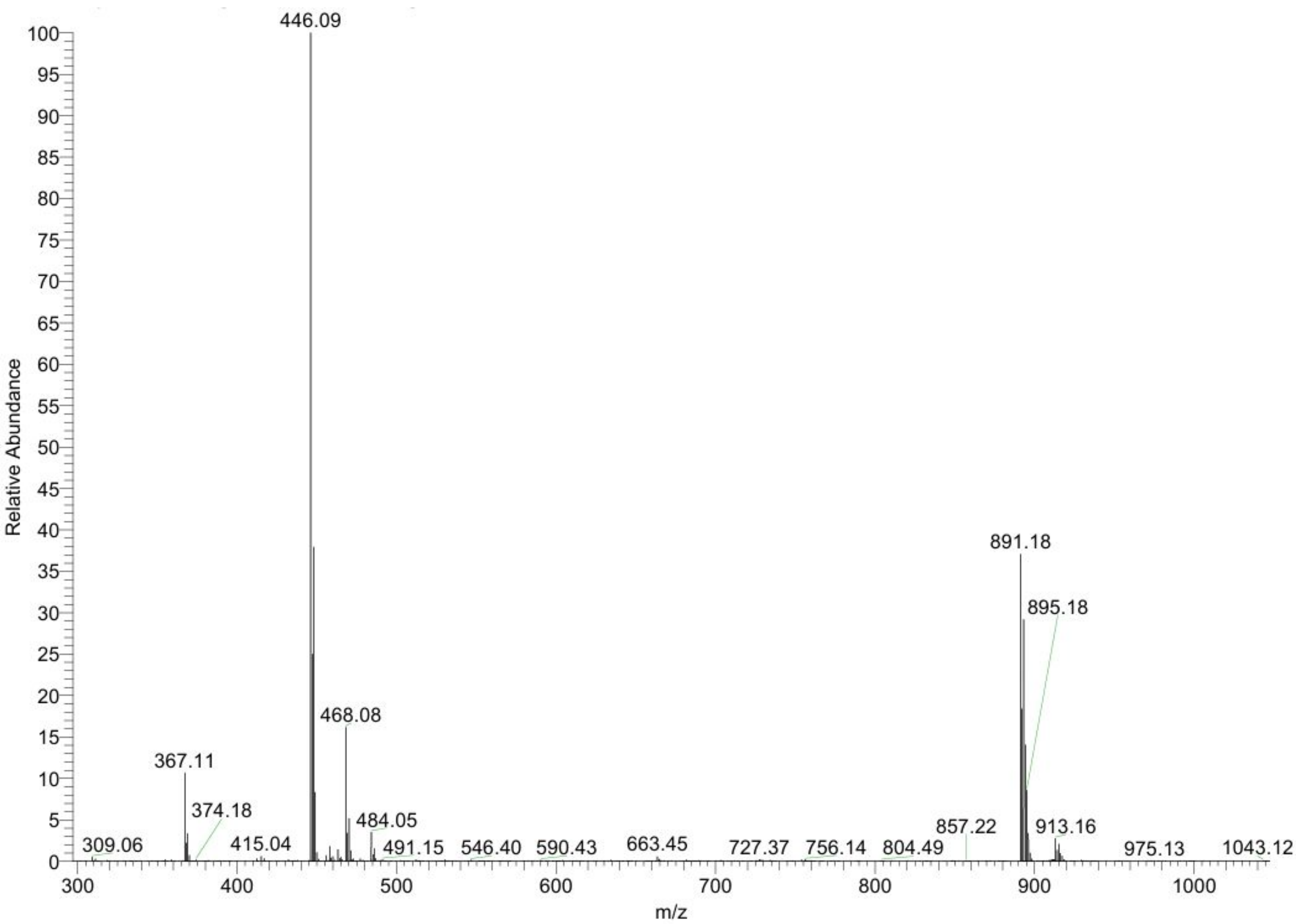

Figure S34. MS spectrum of **3h**

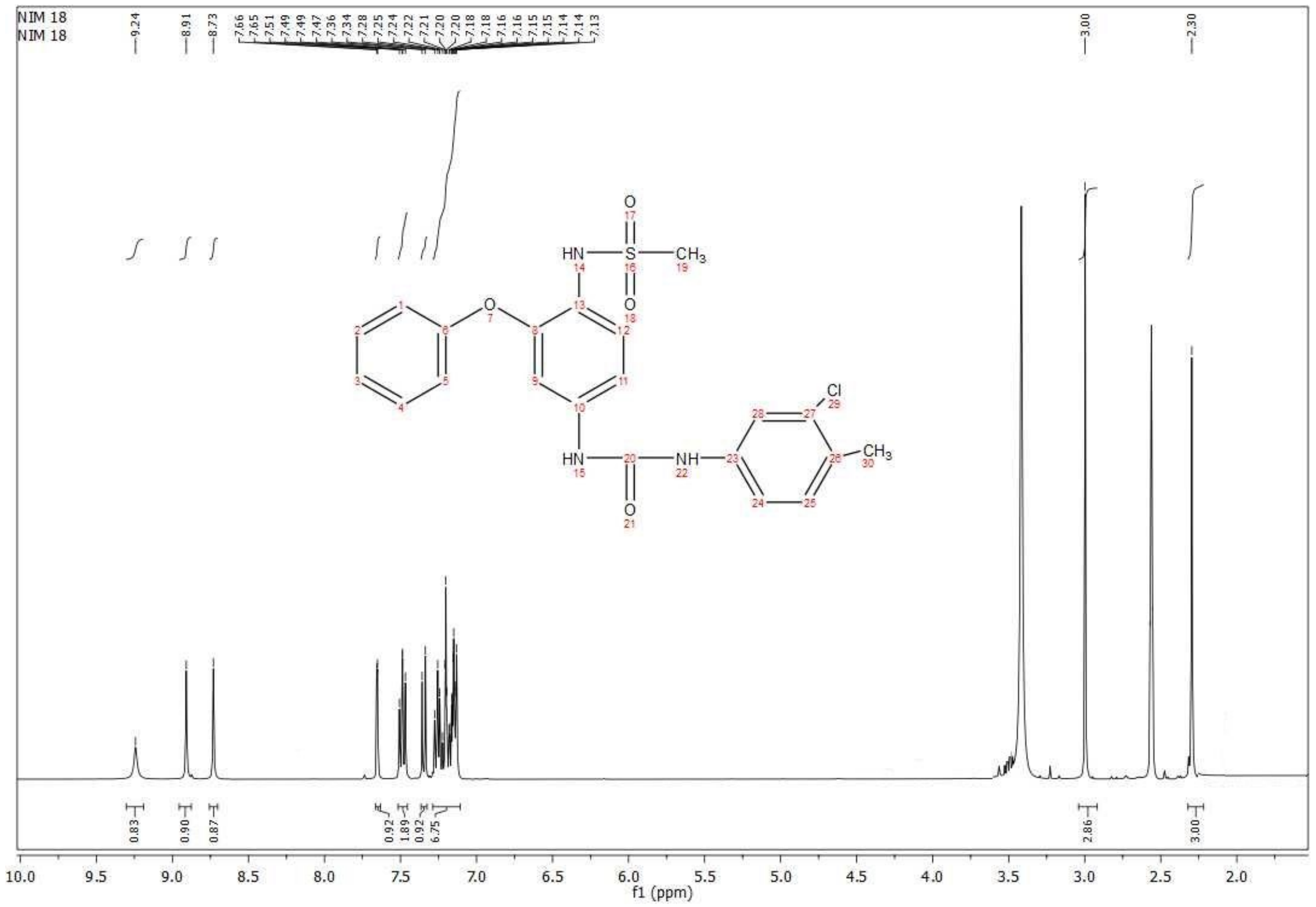

Figure S35. ^{1}H NMR spectrum (600 MHz, DMSO) of **3h.**

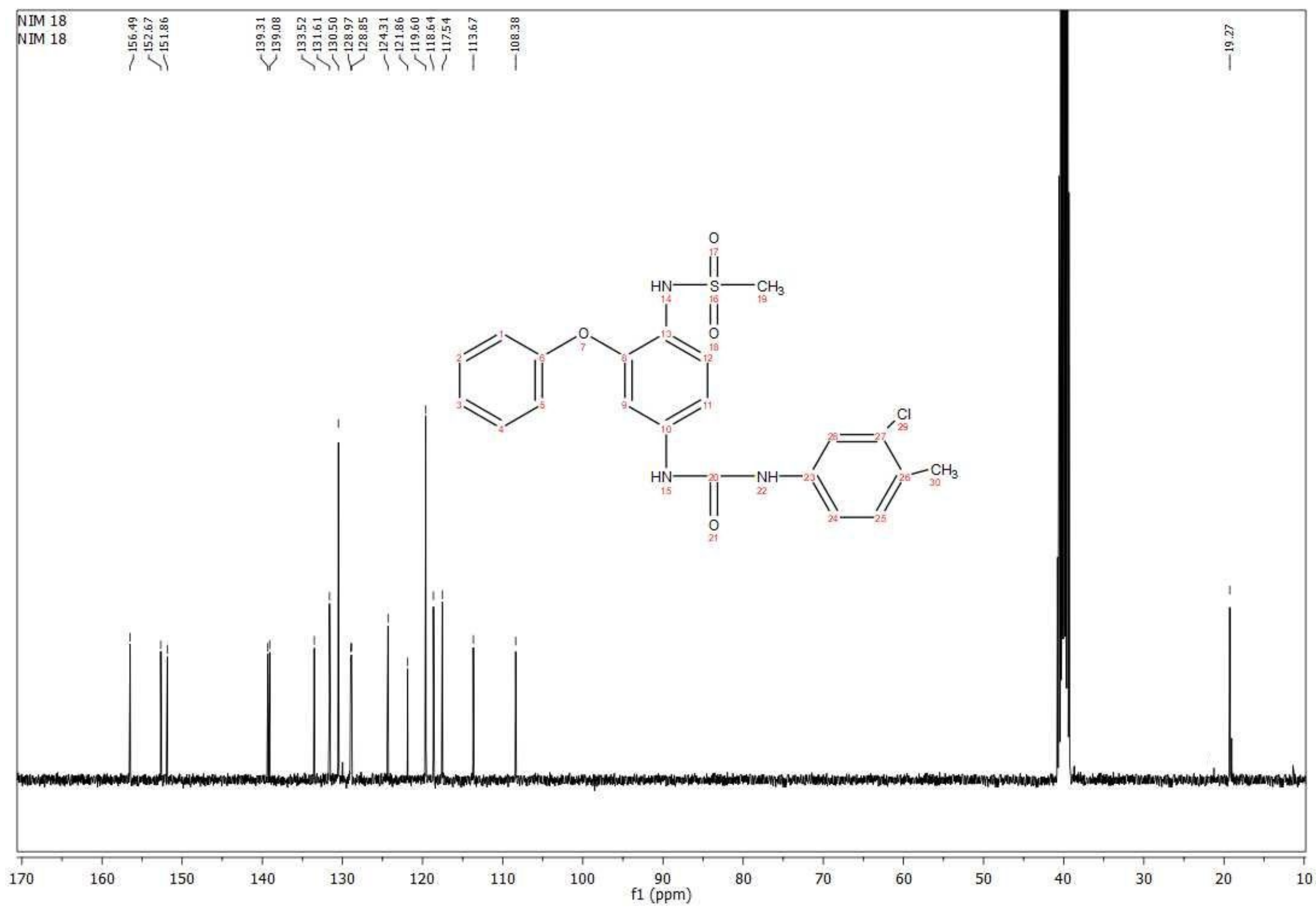

Figure S36. ^{13}C NMR spectrum (150 MHz, DMSO) of **3h.**

Copies of the FTIR, MS, ^{1}H NMR and ^{13}C NMR spectra of synthesized N-{4-[3-(2,4-Difluoro-phenyl)-ureido]-2-phenoxy-phenyl}-methanesulfonamide (3i)

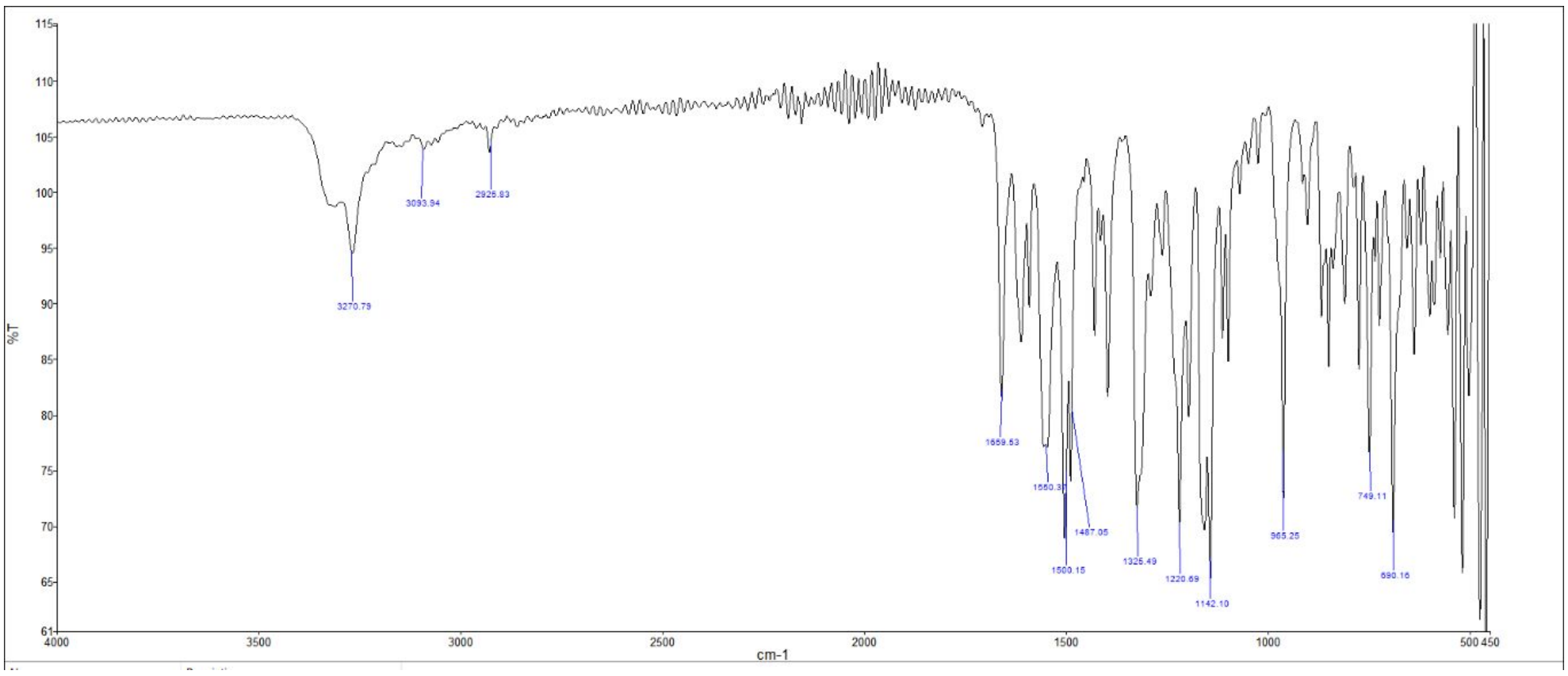

Figure S37. FTIR spectrum of **3i**

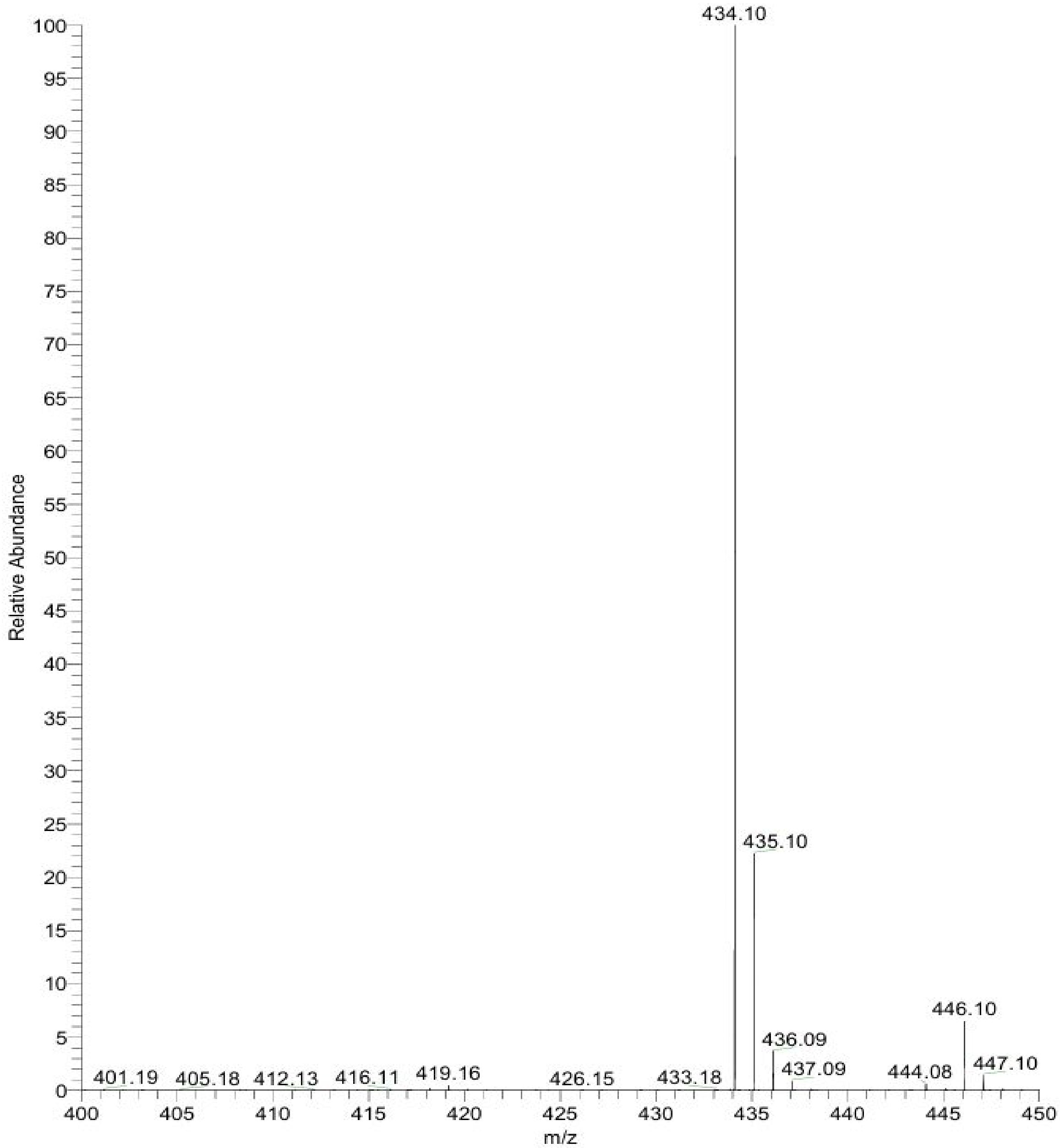

Figure S38. MS spectrum of **3i**

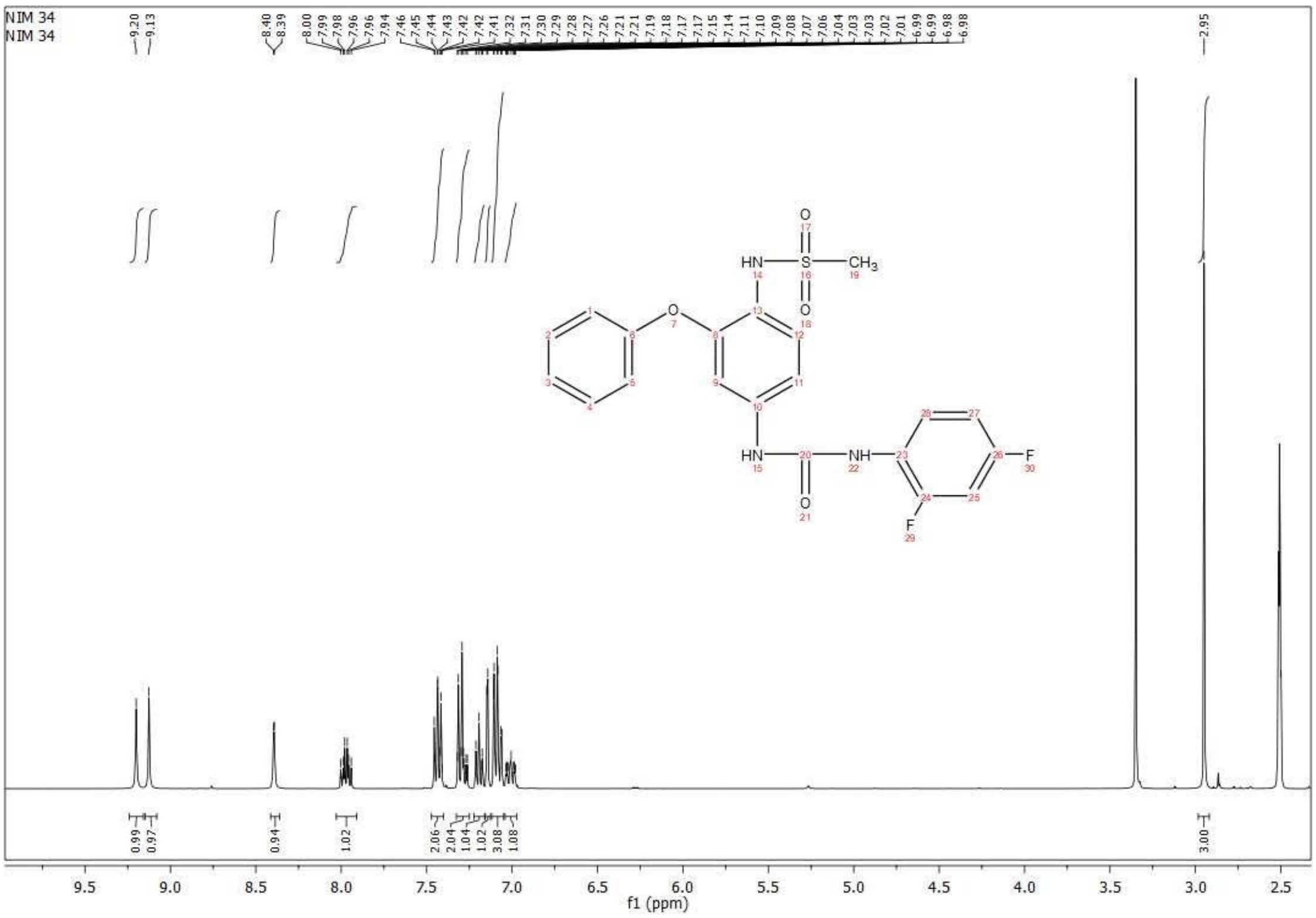

Figure S39. ^{1}H NMR spectrum (600 MHz, DMSO) of **3i.**

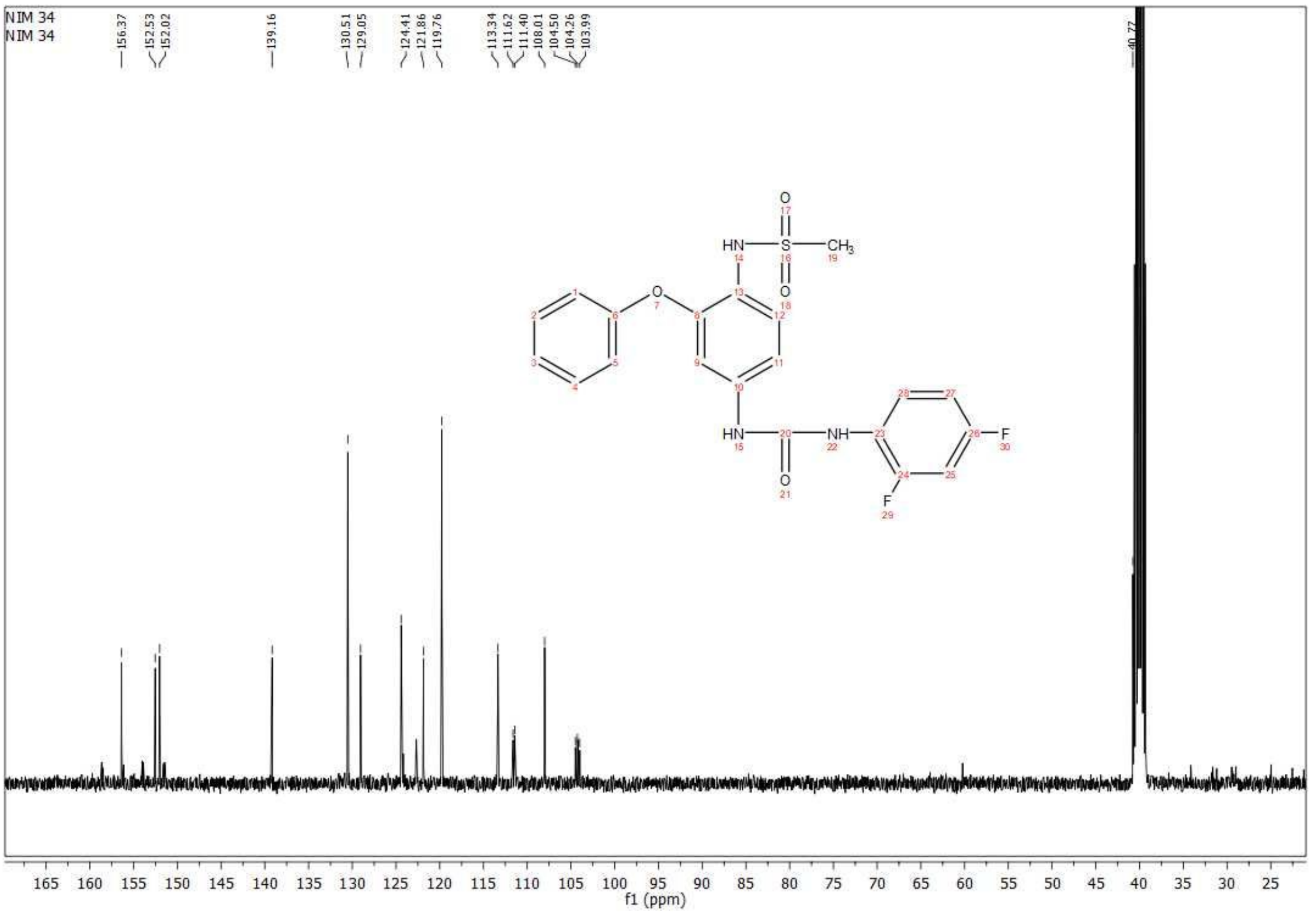

Figure S40. ^{13}C NMR spectrum (150 MHz, DMSO) of **3i.**

Copies of the FTIR, MS, ^{1}H NMR and ^{13}C NMR spectra of synthesized N-{2-Phenoxy-4-[3-(2-trifluoromethyl-phenyl)-ureido]-phenyl}-methanesulfonamide (3j)

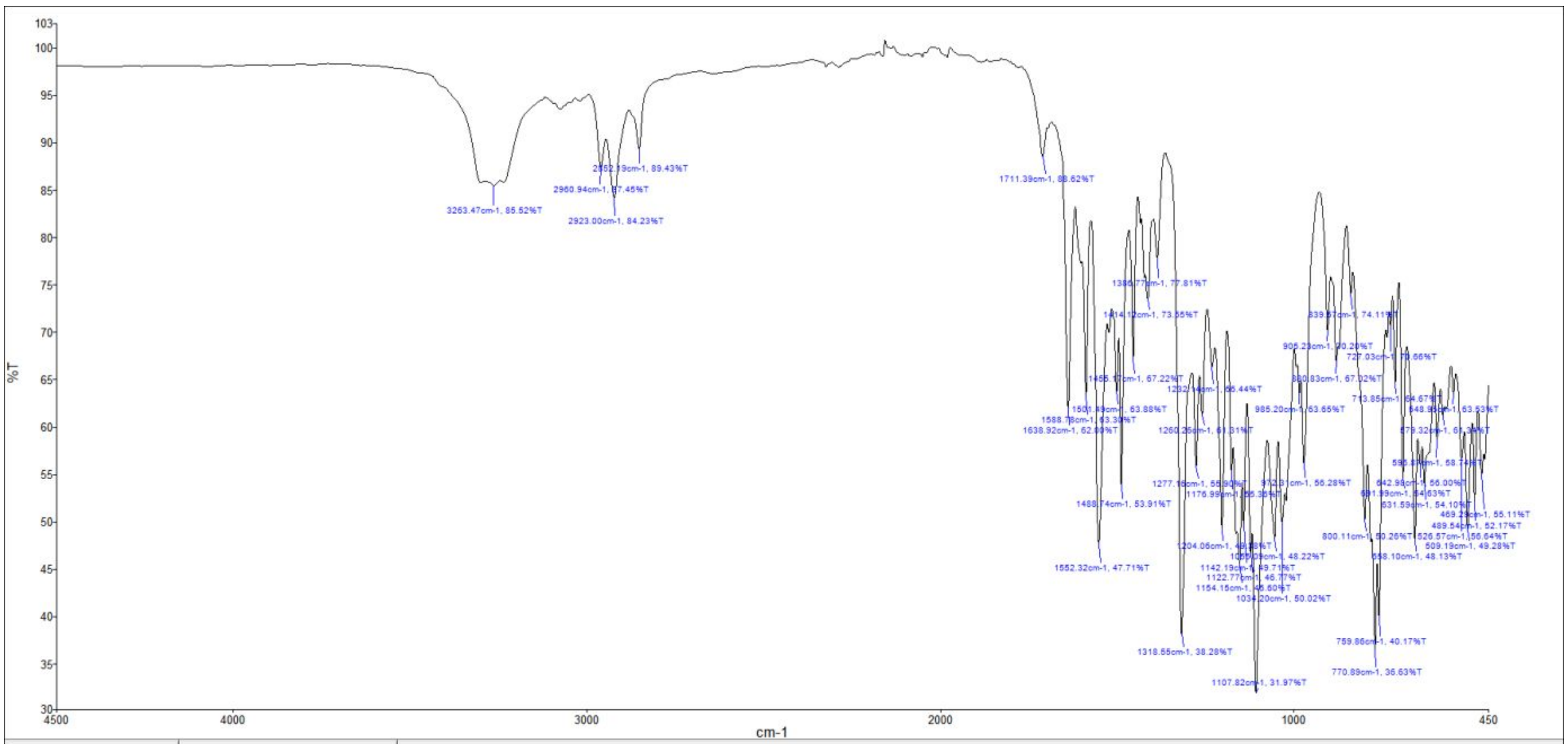

Figure S41. FTIR spectrum of **3j.**

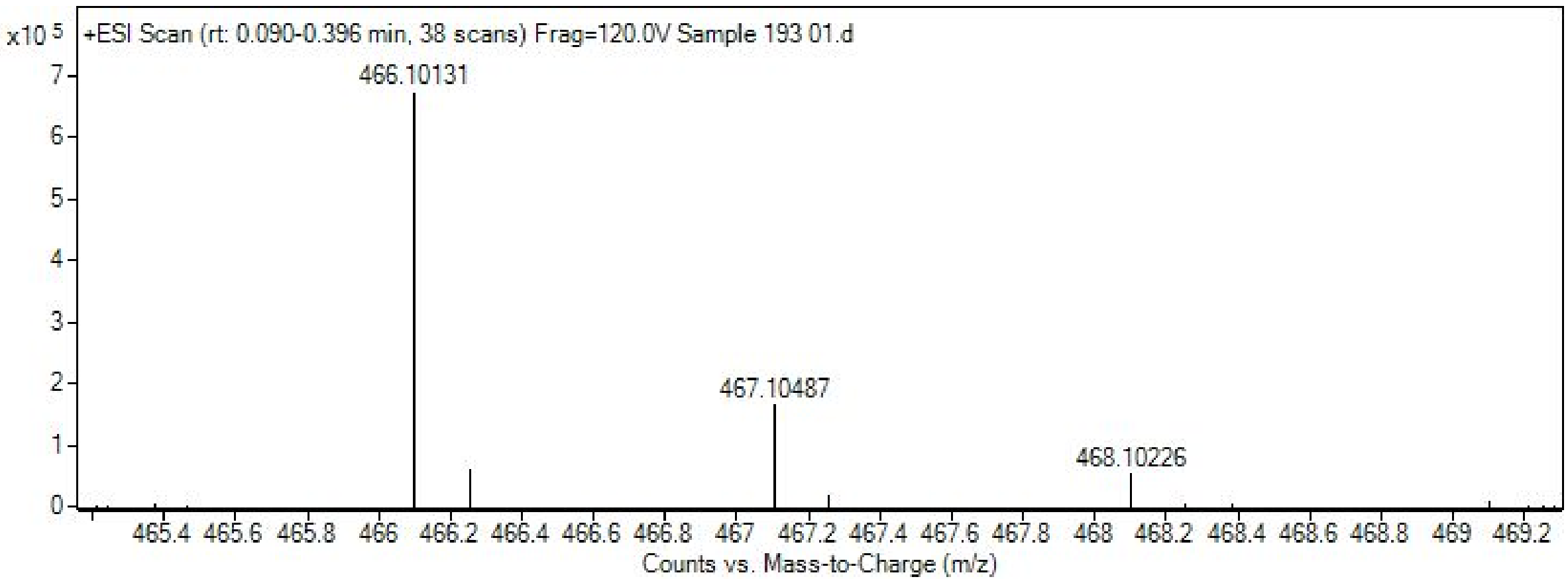

Figure S42. MS spectrum of **3j.**

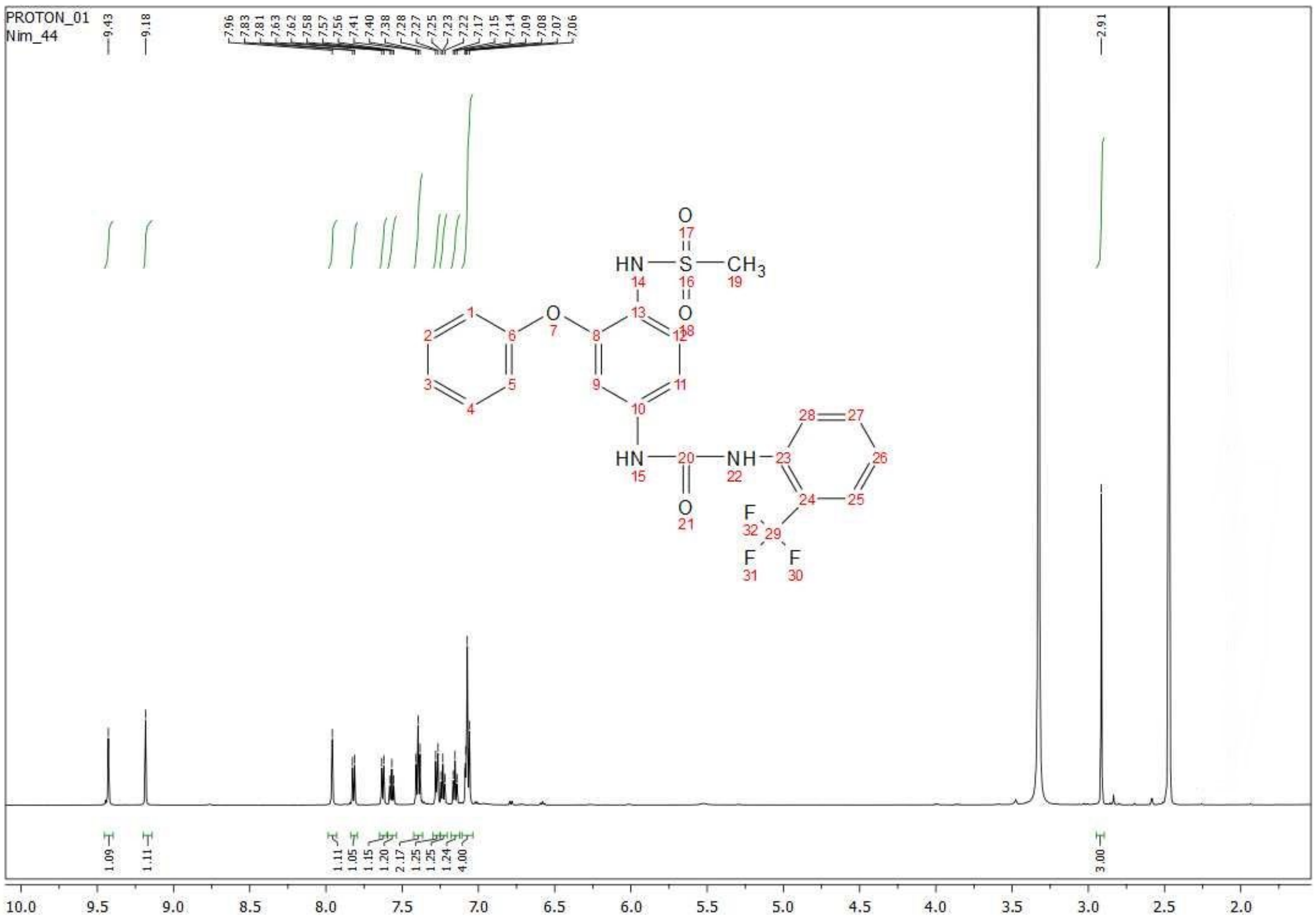

Figure S43. ^{1}H NMR spectrum (600 MHz, DMSO) of **3j.**

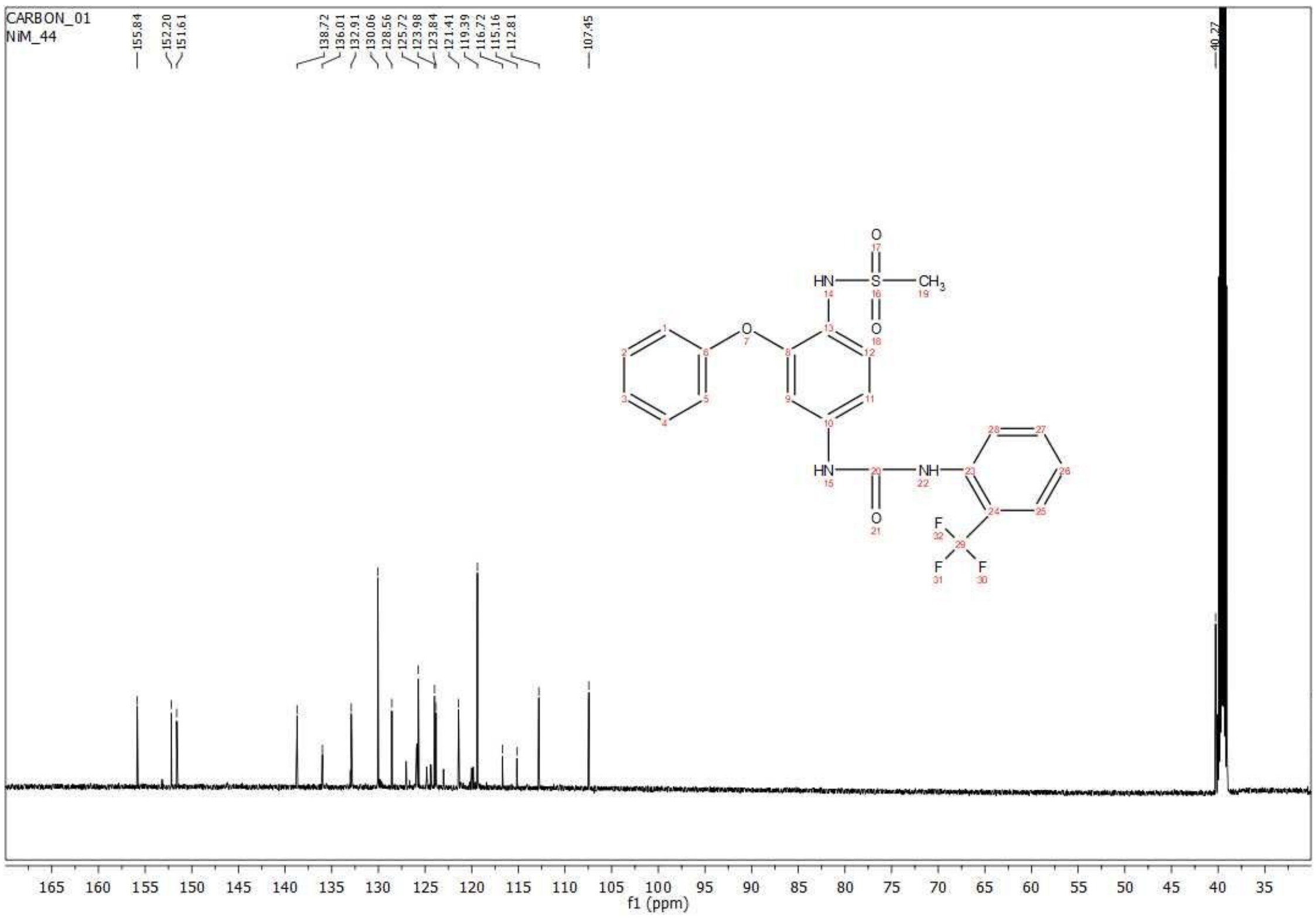

Figure S44. ^{13}C NMR spectrum (150 MHz, DMSO) of **3j.**
